# Synthesis, characterization, and *in vitro* and *in silico* α-glucosidase inhibitory evolution of novel *N*′-(2-cyclopentyl-2-phenylacetyl)cinnamohydrazide derivatives†

**DOI:** 10.1039/d5ra01971k

**Published:** 2025-05-21

**Authors:** Ram Reddy Mudireddy, Rambabu Gundla, Baji Baba Shaik, Anoop Bodapati, Panasa Mahesh, Shiva Sravan Naidu, Damodar Tirumalasetti, Naresh Kumar Katari

**Affiliations:** a Department of Chemistry, GITAM School of Science, GITAM (Deemed to be University) Hyderabad Telangana 502 329 India rgundla@gitam.edu; b B.V.Raju Institute of Technology Vishnupur, Narsapur, Medak Dist Telangana 502313 India; c School of Chemistry & Physics, College of Agriculture, Engineering & Science, University of KwaZulu-Natal Westville Campus, P Bag X 54001 Durban 4000 South Africa KatariN@ukzn.ac.za; d Analytical Research & Development, Hikma Pharmaceuticals USA Inc Columbus Ohio 43228 USA; e R&D, Vertex Pharmaceuticals 316 Northern Avenue Boston MA 02210 USA; f Department of Chemistry, University of Pennsylvania New Town PA 18940 USA

## Abstract

To discover potential α-glucosidase inhibitory agents, a new series of *N*′-(2-cyclopentyl-2-phenylacetyl)cinnamohydrazide derivatives were designed and synthesized as α-glucosidase inhibitors. The newly synthesized compounds were characterized using ^1^H, ^13^C NMR, and mass spectroscopy analysis and evaluated for their *in vitro* α-glucosidase inhibitory effects. All the tested compounds displayed significant α-glucosidase inhibitory activity compared to the standard drug acarbose. Among all, compounds 7b, 7d and 6g exhibited the strongest inhibition with IC_50_ values of 14.48 nmol, 18.88 nmol and 28.51 nmol, respectively. Molecular docking analysis was conducted to identify the important binding interactions responsible for inhibition activity of a-glucosidase. The compounds 7b and 7d exhibit the highest docking energies, with same value of −10.1 kcal mol^−1^ with crucial hydrogen bonding interactions with HIS:280 and ASN:415, respectively. Furthermore, computational drug likeness/ADME/toxicity analysis was conducted on the compounds, which indicated that these compounds exhibit drug-like properties and possess favourable ADME and toxicity profiles.

## Abstract 2

To discover potential α-glucosidase inhibitory agents, a new series of *N*′-(2-cyclopentyl-2-phenylacetyl)cinnamohydrazide derivatives were designed and synthesized as α-glucosidase inhibitors.

## Graphical abstract

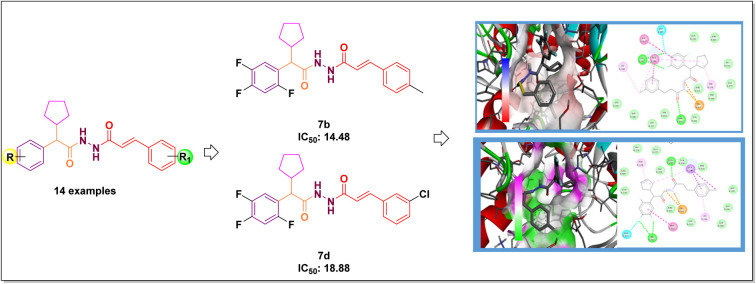

## 1 Introduction

A well-known metabolic disease, diabetes mellitus, has emerged as a major public health concern in the modern era owing to the devastating consequences it can have on an individual's wellness over time.^1,2^ Specifically, type 2 diabetes (T2DM) accounts for the most encountered form of diabetes.^3,4^ Major risk factors for developing diabetes include abnormalities in glucose metabolism.^5^ The hallmark of type 2 diabetes is an irregular rise in blood glucose levels.^6,7^ Acute hyperglycaemia damages blood vessels by acting directly on the vascular endothelium; this damages the patient's quality of life and eventually leads to heart attacks, strokes, retinopathy, and coronary heart disease.^8,9^ Currently, the US FDA has approved five main classes of oral antidiabetic drugs: biguanides, thiazolidinediones, sulfonylureas, meglitinides and α-glucosidase inhibitors.^10–12^ Unfortunately, some patients experience intolerable adverse effects from these drugs, while others see their effectiveness wane with time.^13,14^ Consequently, there has been and will be immense interest in the development for novel anti-diabetic drugs. Among these drugs, oral anti-diabetic drugs such as α-glucosidase inhibitors reduce postprandial hyperglycemia by inhibiting the enzyme a-glucosidase, which is responsible for the carbohydrate's degradation.^15–17^ The Third Asia-Pacific Region Diabetes Treatment Guidelines have recommended α-glucosidase inhibitors as the first line for decreasing postprandial hyperglycemia due to their several advantages.^18^

**Fig. 1:**
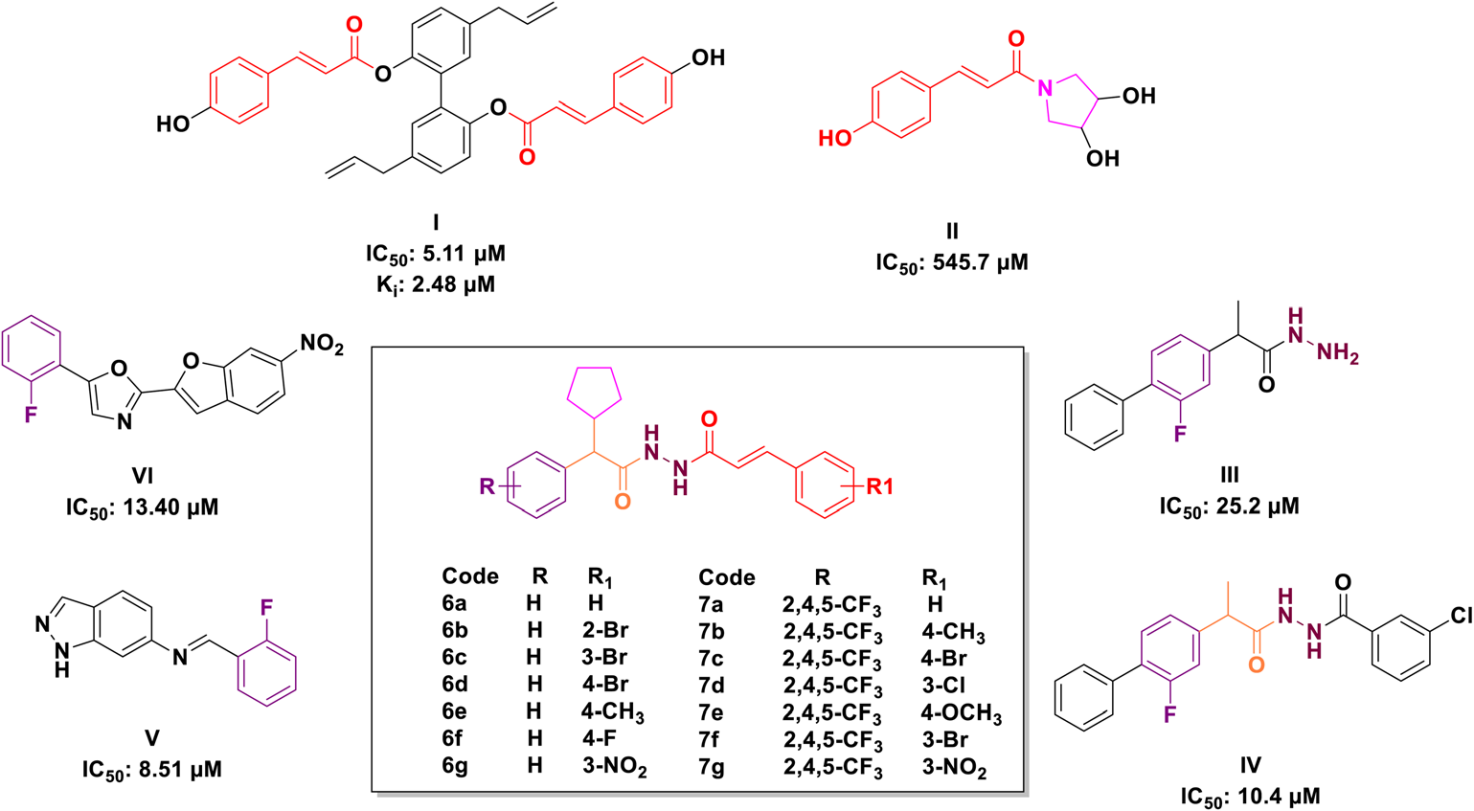
Rationale design of targeted compounds 6a–g and 7a–g.

α-Glucosidase is an essential enzyme in the amylase family, significantly contributing to carbohydrate metabolism in organisms.^19–21^ So, by limiting α-glucosidase activity, intestinal glycolysis can be diminished, leading to a reduction in postprandial hyperglycemia.^22,23^ At now, α-glucosidase inhibitors, namely acarbose, voglibose, and miglitol (Fig. 1A), have been developed in the clinical treatment of T2DM.^24,25^ However, prolonged use of these drugs may result in gastrointestinal adverse effects.^26–29^ Therefore, it is essential to develop novel α-glucosidase inhibitors to tackle diabetes. To date, many research groups are developed novel α-glucosidase inhibitors and some of these compounds are under preclinical evaluation.^30–34^

Cinnamic acid, a significant component of *Cinnamomum cassia Presl*, exhibits several biological activities,^35^ including anticancer,^36–42^ antioxidative,^43^ cardioprotective,^44,45^ antibacterial^46,47^ and antidiabetic^48,49^ properties. The structural alteration of cinnamic acid is garnering increasing attention to generate more potent molecules.^36^ Cinnamic acid derivatives (Fig. 1) and cinnamic acid ester derivatives (Fig. 1) found to have significant inhibitory activity against α-glucosidase. Chun *et al.* reported twenty novel cinnamic acid magnolol derivatives and screened for their anti-hyperglycaemic potency (I, IC_50_: 5.11 μM).^50^ Siva Prasad *et al.* reported novel deacetylsarmentamide derivatives (II) and evaluated for their *in vitro* α-glucosidase inhibitory potency with an excellent result.^51^

On the other hand, hydrazine's (III & IV, Fig. 1) bonds are prevalent in bioactive and drug candidates and emerged as a favourable ligand in drug discovery because of their unique properties, which include polarity, protein binding, and the proton exchange rate.^52^ Similarly, compounds with phenyl rings were evaluated for their α-glucosidase inhibitory properties and identified as potential lead agents for type II diabetes mellitus (V & VI, Fig. 1).^50,53^ Taha *et al.* described several compounds containing fluorine atoms linked to the benzene ring, demonstrating significant α-glucosidase inhibitory potential, and demonstrated that the fluorine group influences the potency of the compounds.^54^

The molecular hybridisation strategy involves the combination of two or more pharmacophores into a single molecule to enhance pharmacological activity.^55,56^ Following the analysis of marketed drugs and prior research in the synthesis of α-glucosidase inhibitors derived from cinnamic acid derivatives, hydrazone and fluorophenyls, we fused these compounds into a single molecule to develop a series of novel α-glucosidase inhibitors (Fig. 1).

## 2 Results and discussion

### 2.1. Chemistry

**Scheme 1:**
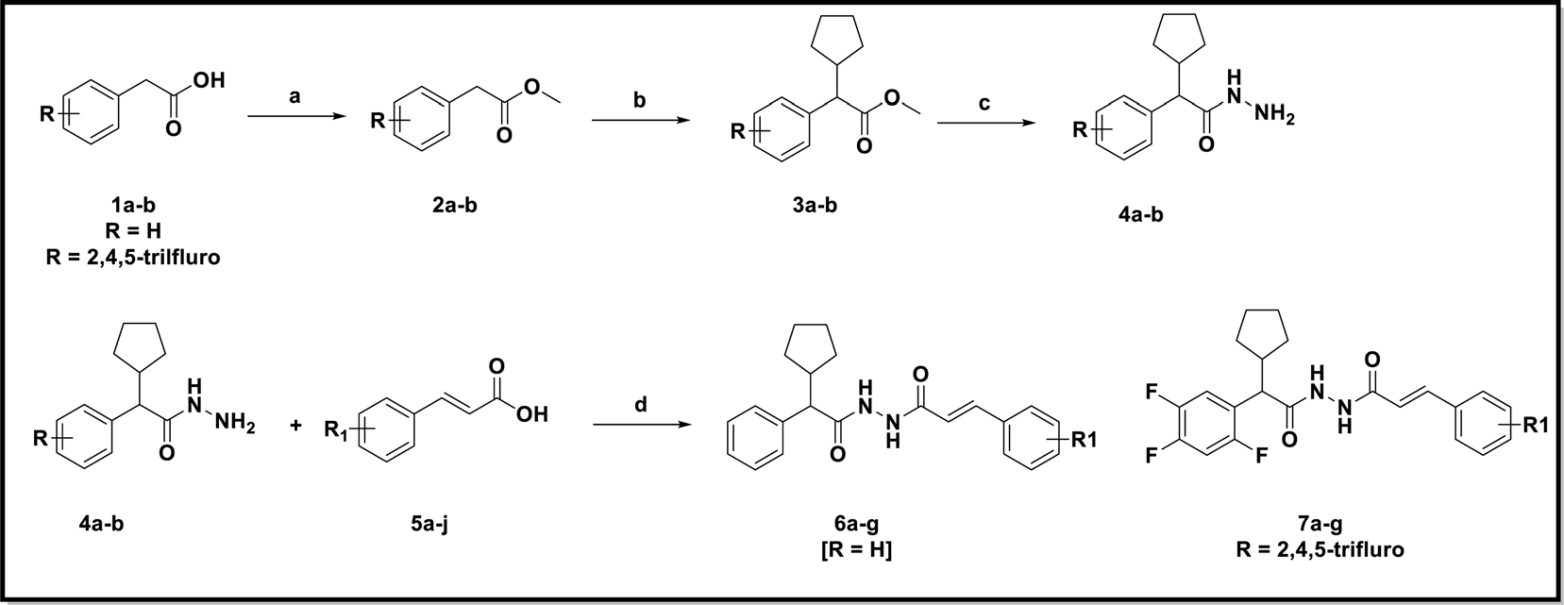
Reagents and conditions: (a) sulfuric acid, methanol, 60–70 °C, yield 95%; (b) potassium *tert*-butoxide, bromo cyclopentane, DMF, 20–30 °C, yield 86%; (c) 98% hydrazine hydride, ethanol, reflux 70 °C, yield 90%; (d) HATU, DIPERA, DMF, rt, 16 h.

The final derivatives 6a–g and 7a–g were prepared by using conventional synthesis route as depicted in Scheme 1. The esterification of commercially available substituted phenyl acetic acid in methanol and catalytic amount of sulfuric acid at 60–70 °C to yielded corresponding methyl ester (2a & 2b). Compound 2a & 2b was alkylated with cyclopentyl bromide in presence of potassium *tert*-butoxide in DMF solvent offered substituted methyl 2-cyclopentyl-2-phenylacetate (3a & 3b). Methyl ester was converted to hydrazide with 98% hydrazine hydrate and ethanol at reflux temperature to give substituted 2-cyclopentyl-2-phenylacetohydrazide (4a & 4b) with appreciable yield. The key intermediates (4a & 4b) was treated with different substituted cinnamic acids to yielded the *N*′-(2-cyclopentyl-2-phenylacetyl)cinnamohydrazide derivatives. The structures of these derivatives were characterized by using IR, ^1^H, ^13^C NMR and mass spectroscopy analysis.

### 2.2. α-Glucosidase inhibitory assay

All the final compound 6a–g and 7a–g were evaluated for their *in vitro* α-glucosidase inhibitory activity. The results were displayed in Table 1. The results displayed that fluoro substitution of phenyl ring A (7a–g) exhibited potential activity compared to unsubstituted phenyl ring A (6a–g). The structure–activity relationship (SAR) was investigated based on the nature and locations of substitutions on the phenyl rings A and B of the *N*′-(2-cyclopentyl-2-phenylacetyl)cinnamohydrazide system. The α-glucosidase activities of all synthesized *N*′-(2-cyclopentyl-2-phenylacetyl)cinnamohydrazide-based candidates, specifically compounds 6a–g and 7a–g, were superior (IC_50_ = 14.48 to 232.72 nmol) in comparison to the standard acarbose (IC_50_ = 35.91 nmol). The unsubstituted phenyl group (6a and 7a) shows the moderate inhibition with an IC_50_ of 79.75 and 85.16 nmol, suggesting that the unsubstituted phenyl ring does not have much effective in key interactions within the enzyme's active site. Among all (7a–g), The most potent compound 7b (IC_50_ = 14.48 nmol) containing electron-withdrawing groups (2,4,5-trifluro) on ring A and electron donating (para-methyl) on ring B found to be increased inhibition and most promising α-glucosidase inhibitor among all the synthetic derivatives due to better interaction with the active pockets of enzymes. Furthermore, compound 7d (IC_50_ = 18.88 nmol) with *m*-Cl substitution on ring B and a 2,4,5-trifluro substituent on aromatic ring A showed better inhibitory activity as compared to the standard acarbose (IC_50_ = 35.91 nmol). Halogenated substituent 4-bromophenyl (7c) and 3-bromophenyl (7f) displayed moderate activity (IC_50_: 49.34 and 33.22 nmol), indicating that bromo substitutions do not enhance binding affinity for α-glucosidase inhibition. A considerable a-glucosidase inhibition was observed by 7g, with an IC_50_ of 54.78 nmol. However, simple aromatic ring substrate (7a) exhibited the moderate inhibition. Compound 7e, introducing a methoxy substituent at *para* position have no effect on inhibition.

**Table 1:** α-Glucosidase inhibition of final derivatives 6a–g and 7a–g

S. no	R	R_1_	Compound code	IC_50_ (nmol)
1	H	H	6a	79.75
2	H	2-Br	6b	104.44
3	H	3-Br	6c	**46.80**
4	H	4-Br	6d	64.80
5	H	4-CH_3_	6e	232.72
6	H	4-F	6f	No inhibition
7	H	3-NO_2_	6g	**28.51**
8	2,4,5-Trifluro	H	7a	85.16
9	2,4,5-Trifluro	4-CH_3_	7b	**14.48**
10	2,4,5-Trifluro	4-Br	7c	49.34
11	2,4,5-Trifluro	3-Cl	7d	**18.88**
12	2,4,5-Trifluro	4-OCH_3_	7e	No inhibition
13	2,4,5-Trifluro	3-Br	7f	33.22
14	2,4,5-Trifluro	3-NO_2_	7g	54.78
15			Acarbose	35.91

**Fig. 2:**
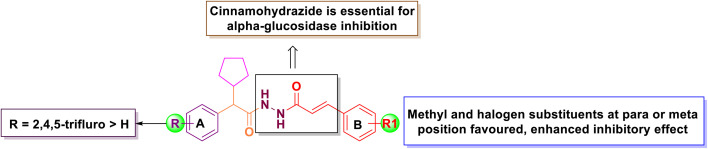
SAR of the synthesized target compounds 6a–g and 7a–g.

In the series of 6a–g, Compound 6c and 6d had significant inhibition against α-Glucosidase with an IC_50_ of 46.80 and 64.80 nmol, respectively. This indicates that bromo substitution had considerable effect in both cases (6 and 7). Compound 6a, which is simple aromatic group exhibited an IC_50_ value of 79.75 nmol. Further, substituting the R_1_ position to a nitro group at *meta* position in 6g enhanced the inhibition (IC_50_ = 28.51 nmol). Compound 6b and 6e showed moderate inhibition. However, 6f and 7e had no inhibition effect. From the results, it could be concluded that the introduction of methyl and halogen in the *para* or *ortho* position of phenyl ring leads to an improved increase of the inhibitory activity. The SAR analysis of final derivatives was shown in Fig. 2.

### 2.3. Docking studies

**Fig. 3:**
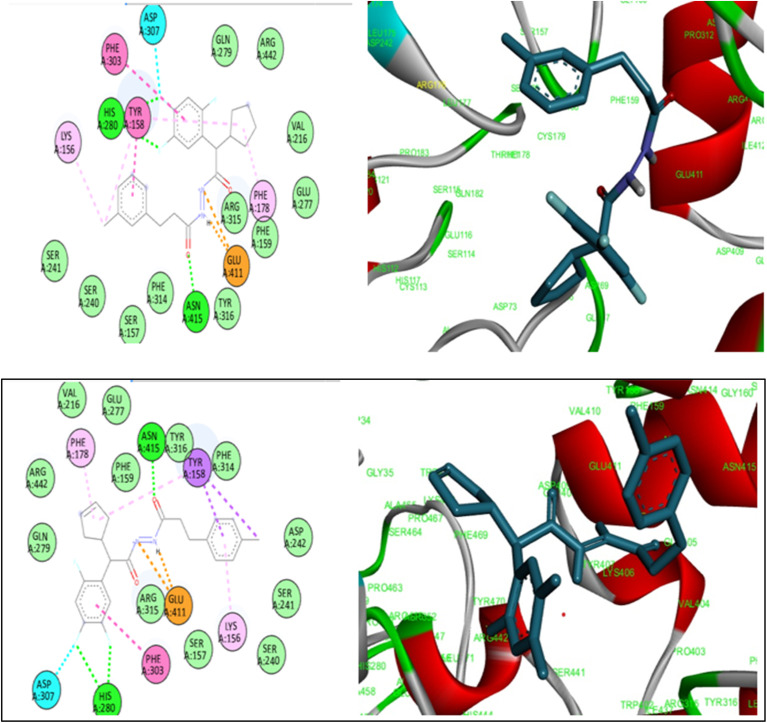
3D & 2D Ligand interaction diagrams for docking compounds A: 7b; B: 7d (PDB ID: 3WY1).

Molecular docking is an effective technique for elucidating the interaction mechanism between a ligand and its receptor, as well as forecasting the potential binding site of the ligand.^57,58^ To further improve and confirm the inhibitory profile synthesized compounds, a thorough screening procedure was carried out using AutoDock Vina (RSC PDD:3WY1) (Fig. 3). Among the compounds that were docked, 7b, and7d had the highest docking energy, whereas 6c had the lowest docking energy. Hybrid 7b and 7d sticks an efficient conformation in the α-glucosidase binding pocket. Compound 7b had a docking energy of −10.1 kcal mol^−1^. Whereas, compounds 7d and 7a exhibited docking energies of −10.1 kcal mol^−1^ and −9.9 kcal mol^−1^, respectively. The 7b formed a conventional hydrogen bonding with HIS:280 and ASN:415. The 7d compound forms conventional hydrogen bonds with HIS:280 and ASN:415. The compound 6c exhibited the lowest docking energy, with −8.9 kcal mol^−1^. In addition, the presence of a conventional hydrogen bond with PRO:312 and ARG:315 were found. These findings were supported to the *in vitro* assay.

### 2.4. Evaluation of physico-chemical properties

**Fig. 4:**
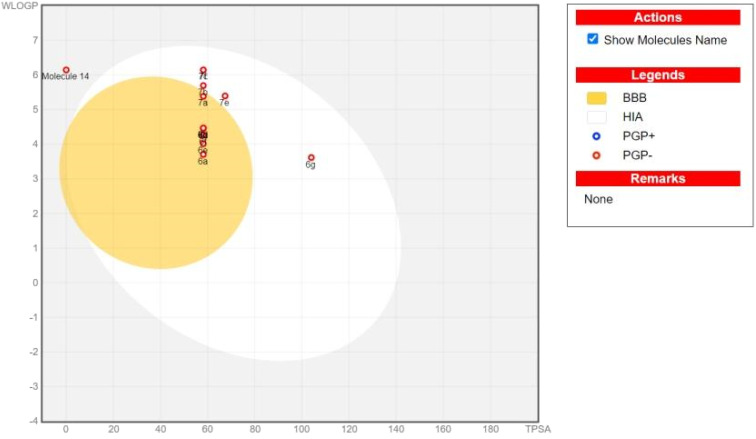
Boiled egg of *N*′-(2-cyclopentyl-2-phenylacetyl)cinnamohydrazide derivatives.

**Fig. 5:**
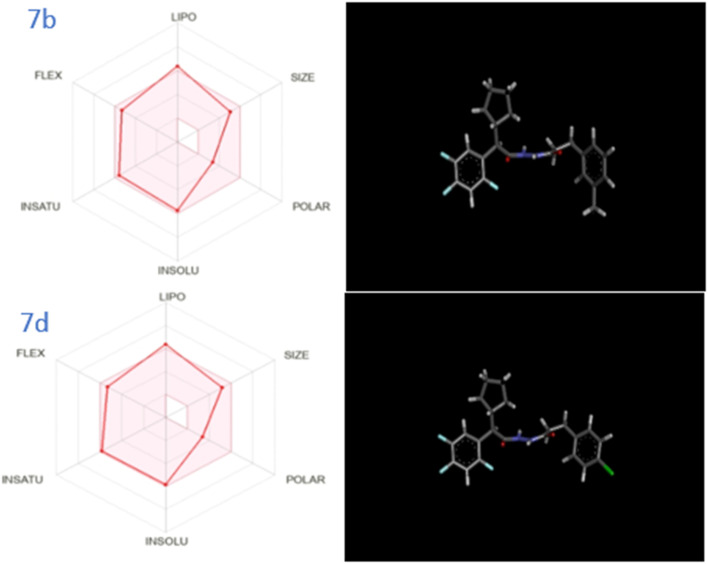
3D representation and web representation of physico-chemical properties of molecule 7b & 7d.

Literature review indicated that drugs with lower molecular weights and more lipophilicity can be absorbed and excreted *via* para cellular and trans cellular routes with greater ease. Possible side effects include mild toxicity and increase renal excretion. A molecule with drug-like qualities (DLM) can be precisely characterized using the rule of five (RO5).^59^ The pharmacokinetic characteristics of *N*′-(2-cyclopentyl-2-phenylacetyl)cinnamohydrazide derivatives 6a–g and 7a–g, such as permeability of the blood–brain barrier (BBB) and gastrointestinal absorption (GIA) have also calculated (Table 2). As illustrated in Fig. 4 and 5, the intestinal or brain estimated permeation technique (BOILED Egg) was computed using the lipophilicity, indicated by the WLOGP, and the topological polar surface area (TPSA). All molecules are expected to be inside the white ellipse and grey region. This implies that certain substances may exhibit poorer BBB properties but superior GIA. All drugs whose ability to transport out of the Central Nervous System (CNS) is dependent on *P*-glycoprotein (PGP).^60,61^ Compounds 7b and 7d have the highest docking energy with −10.1 kcal mol^−1^ and −10.1 kcal mol^−1^.

**Table 2:** Physico-chemical and pharmacokinetic properties of *N*′-(2-cyclopentyl-2-phenylacetyl)cinnamohydrazide derivatives 6a–g and 7a–g

Compound	Physico-chemical properties										Pharmacokinetic properties		
	MW (g mol^−1^)	HA	AHA	RBs	HBA	HBD	MR	TPSA	iLOGP	Violation	GI absorption	BBB permeant	PgP substrate
6a	348.44	26	12	8	2	2	103.37	58.20	3.13	0	High	Yes	No
6b	427.33	27	12	8	2	2	111.07	58.20	3.15	1	High	Yes	No
6c	427.33	27	12	8	2	2	111.07	58.20	2.88	1	High	Yes	No
6d	427.33	27	12	8	2	2	11.07	58.20	3.15	1	High	Yes	No
6e	362.46	27	12	8	2	2	108.33	58.20	2.97	0	High	Yes	No
6f	366.43	27	12	8	3	2	103.32	58.20	3.18	0	High	Yes	No
6g	393.44	29	12	9	4	2	112.19	104.02	2.77	0	High	No	No
7a	402.41	29	12	8	5	2	103.24	58.20	3.24	1	High	Yes	No
7b	416.44	30	12	8	5	2	108.21	58.20	3.81	1	High	No	No
7c	481.31	30	12	8	5	2	110.94	58.20	3.37	1	High	No	No
7d	427.33	27	12	8	2	2	111.07	58.20	3.15	1	High	Yes	No
7e	432.44	31	12	9	6	2	109.43	67.43	3.65	1	High	No	No
7f	481.31	30	12	8	5	2	110.94	58.20	3.76	1	High	No	No
7g	447.41	32	12	9	7	2	112.06	104.02	3.03	1	High	No	No

## 3 Conclusion

In conclusion, we have synthesized a new series of *N*′-(2-cyclopentyl-2-phenylacetyl)cinnamohydrazide derivatives in moderate to excellent yields and characterized by using FT-IT, ^1^H NMR, ^13^C NMR and HRMS spectral analysis. Thereafter, the target compounds 6a–g and 7a–g were assessed for *in vitro* activity in inhibiting α-glucosidase. Most of the synthesized compounds exhibited α-glucosidase inhibitory potency with IC_50_ values ranging from IC_50_ of 14.48 to 232.72 nmol as compared to standard acarbose (IC_50_ = 35.91 nmol). Compounds 7b and 7d showed the highest potency with IC_50_ values of 14.48 and 18.88 μM, respectively, as compared to standard acarbose, followed by 6g and 7f with IC_50_ values of 28.51 and 33.22 nmol, respectively. SAR of synthesized target hybrids were established. SAR indicated that compounds containing halogen and methyl substituents on the phenyl ring of cinnamohydrazide exhibited promising α-glucosidase as compared to those with electron-withdrawing groups. Molecular docking results agreed with *in vitro* biological assay data by forming key conventional hydrogen bonding interactions with HIS:280 and ASN:415 within the α-glucosidase binding pocket. Further, all the novel compounds are under druglike and conform the rules of ADME and toxicity profile. In summary, this study has identified a new family of *N*′-(2-cyclopentyl-2-phenylacetyl)cinnamohydrazide derivatives that could serve as lead compounds for the development of novel α-glucosidase inhibitors.

## 4 Experimental section

### 4.1. General information

#### 4.1.1 Materials

Bromo cyclopentane, potassium *tert*-butoxide (Avra Synthesis/Afa-Aesar, India), substituted phenylacetic acid (Sigma-Aldrich, India), various substituted cinnamic acid derivatives (TCI, BLD-Pharma, India), sulfuric acid (Finar, India), HPLC-grade DMF, methanol and ethanol (Sigma-Aldrich, India), HATU (Sigma-Aldrich, India).

#### 4.1.2 Instrumentation

FT-IR spectra obtained on Bruker instrument by using spectra manager software. Sample was prepared by taking 3–5 mg of compound on 250–300 mg of KBr salt. Advance – III HD Bruker 300 & 500 MHz NMR spectrometer (Bruker Corporation, Billerica, Massachusetts, USA) was used to record ^1^H & ^13^C NMR spectra with CDCl_3_ and DMSO-*d*_6_ as solvents. Chemical shifts are presented in parts per million, downfield from Tetramethyl silane as internal standard. High resolution mass spectrometry spectra (HRMS) were recorded by using the ESI technique, positive mode, capillary 4500, 0.4 bar, dry gas 4.0 L min^−1^. Solvents and reagents were used directly from the manufacturer or purified when required by standard procedures.

### 4.2. Chemistry

#### 4.2.1 Synthesis of substituted phenylacetic acid methyl ester (2a & 2b)

Substituted phenylacetic acid methyl ester was synthesized by reacting phenylacetic acid (40 g, 1.0 eq.) with sulfuric acid (0.1 vol.) in methanol (10 vol.) medium at 60–70 °C to give substituted phenylacetic acid methyl ester.

##### 4.2.1.1 Methyl 2-(2,4,5-trifluorophenyl)acetate (2b)

Liquid mass: yield 25 g, 94%, ^1^H NMR (300 MHz, CDCl_3_): *δ* (ppm): 7.16–7.08 (m, 1H), 6.97–6.89 (m, 1H), 3.72 (s, 3H), 3.61 (s, 2H). HRMS (ESI) *m*/*z* calcd. For [C_9_H_7_F_3_O_2_]^+^: 204.15 [M + H]^+^, found 203.

#### 4.2.2 Synthesis of substituted methyl-2-cyclopentyl-2-phenylacetate (3a & 3b)

To a solution of phenylacetic acid methyl ester (20 g, 1.0 eq.) in DMF (5 vol.) at 20–30 °C, potassium *tert*-butoxide (26.9 g, 1.8 eq.) was added in small portions under nitrogen atmosphere over a period of 20–30 min at 20–30 °C. After completion of addition the reaction mass was allowed to be stirred at 20–30 °C for 60 min. There after bromo cyclopentane (23.85 g, 1.2 eq.) in 3 vol of DMF was added to the reaction mass at 20–30 °C over a period of 30–40 min under nitrogen atmosphere. After completion of addition, the reaction mixture was allowed to stir at 20–30 °C for 16 h. The completion of reaction was monitored by using TLC, after completion of the reaction, cold water was added followed by ethyl acetate, and the organic layer was separated. The aqueous layer was again extracted with ethyl acetate (3 × 20 mL). The combined organic layers were washed with water and dried over sodium sulphate and evaporated under vacuum at temperature below 50 °C to obtain the substitutes methyl-2-cyclopentyl-2-phenylacetate (3a and 3b).

##### 4.2.2.1 Methyl 2-cyclopentyl-2-phenylacetate (3a)

Liquid mass: yield 25 g, 86%. ^1^H NMR (300 MHz, DMSO-d_6_): *δ* (ppm): 7.32–7.23 (m, 5H), 3.57 (s, 3H), 3.38–3.35 (d, *J* = 11.1 Hz 1H), 2.50–2.42 (m, 1H), 1.83–1.74 (m, 1H), 1.65–1.13 (m, 6H), 1.03–0.94 (m, 1H). HRMS (ESI) *m*/*z* calcd. For [C_14_H_18_O_2_]^+^: 218.3 [M + H]^+^, found 219.7.

##### 4.2.2.2 Methyl 2-cyclopentyl-2-(2,4,5-trifluorophenyl)acetate (3b)

Viscous liquid: yield 30.5 g, 84%. ^1^H NMR (300 MHz, CDCl_3_): *δ* (ppm): 7.39–7.31 (s, 1H), 6.97–6.58 (s, 1H), 3.72 (s, 1H), 3.68 (s, 3H), 2.45–2.42 (s, 1H), 1.90–1.86 (m, 1H), 1.66–1.45 (m, 6H), 1.30–1.23 (m, 1H). HRMS (ESI) *m*/*z* calcd. For [C_14_H_15_F_3_O_2_]^+^: 272.27 [M + H]^+^, found 272.27.

#### 4.2.3 Synthesis of substitutes 2-cyclopentyl-2-phenylacetohydrazide (4a & 4b)

To a solution of substituted methyl 2-cyclopentyl-2-phenylacetate (3a or 3b) (20 g, 1.0 eq.) in 100 mL of ethanol was added 98% hydrazine hydride (18.3 g, 4.0 eq.) at 20–30 °C and the reaction mixture was stirred at 80 °C for 24 h. After completion, the reaction mixture concentrated under reduced pressure, obtained solid washed with pentane to offered the substitutes 2-cyclopentyl-2-phenylacetohydrazides 4a and 4b, yield: 18 g, 90%.

##### 4.2.3.1 2-Cyclopentyl-2-phenylacetohydrazide (4a)

Viscous liquid: yield 22.3 g, 89%, ^1^H NMR (300 MHz, DMSO-d_6_): *δ* (ppm): 9.18 (s, 1H), 7.34–7.16 (m, 5H), 4.22 (b, 2H), 3.06–3.02 (d, *J* = 11.1 Hz, 1H), 2.57–2.54 (m, 1H), 1.72–1.27 (m, 7H), 0.93–0.86 (m, 1H). HRMS (ESI) *m*/*z* calcd. For [C_13_H_18_N_2_O]^+^: 218.3 [M + H]^+^, found 219.8.

##### 4.2.3.2 2-Cyclopentyl-2-(2,4,5-trifluorophenyl)acetohydrazide (4b)

Viscous liquid: yield 22 g, 88%, ^1^H NMR (300 MHz, CDCl_3_): *δ* (ppm): 5.78 (s, 1H), 5.29 (s, 1H), 3.69 (s, 3H), 2.21 (t, *J* = 2.4 Hz, 6H). HRMS (ESI) *m*/*z* calcd. For [C_13_H_15_F_3_N_2_O]^+^: 272.27 [M + H]^+^, found 273.1.

#### 4.2.4 Synthesis of substituted *N*′-(2-cyclopentyl-2-phenylacetyl)cinnamohydrazide derivatives (6a–g & 7a–g)

A mixture of cinnamic acid derivative (1.0 eq.), HATU (1.5 eq.) and DIPEA (2.5 eq.) in DMF (10 vol.) was stirred at 20–30 °C for 1 hour. Then substitutes 2-cyclopentyl-2-phenylacetohydrazide (4a/4b, 1 eq.) was added, and the resulting mixture was stirred at 20–30 °C overnight. After completion of reaction, the reaction mixture poured into ice cold water, followed by ethyl acetate (10 vol.). The organic layer was separated, washed with 10% NaCl solution, dried over sodium sulphate and evaporated under vacuum to obtain crude product. These crude products were purified by column-chromatography (EtOAc/Hexane) to afford pure targeted compounds 6a–g and 7a–g.

##### 4.2.4.1 *N*′-(2-Cyclopentyl-2-phenylacetyl)cinnamohydrazide (6a)

Off-white solid, 113 mg, 71% yield, IR (KBr, cm^−1^): 3200, 3058, 3025, 2945, 2862, 1640, 1590, 1494, 1462, 1329, 1184, 1157, 1072, 1030, 967, 855, 756, 735, 695, 656, 562, 531. ^1^H NMR (300 MHz, DMSO-*d*_6_): *δ* (ppm): 10.3–10.14 (b, 1H), 7.58–7.12 (m, 10H), 6.66–6.61 (d, *J* = 15.9 Hz, 1H), 2.58–2.2 (m, 1H), 1.98–1.28 (m, 7H), 0.99–0.92 (m, 1H). ^13^C NMR spectrum, *δ*_c_, ppm, DMSO-*d*_6_: 171.26, 163.45, 140.06, 140.01, 134.57, 129.76, 128.98, 128.11, 127.64, 126.68, 119.40, 55.05, 42.41, 30.76, 30.23, 24.65, 24.46. HRMS (ESI) *m*/*z* calcd. For [C_22_H_24_N_2_O_2_]^+^: 348.45 [M + H]^+^, found 349.5.

##### 4.2.4.2 3-(2-Bromophenyl)-*N*′-(2-cyclopentyl-2-phenylacetyl)acrylohydrazide (6b)

Off-white solid, 137 mg, 70% yield, IR (KBr, cm^−1^): 3201, 2947, 2859, 1639, 1589, 1472, 1324, 1268, 1200, 1183, 1023, 964, 853, 796, 755, 743, 723, 700, 658, 582, 543. ^1^H NMR (300 MHz, DMSO-*d*_6_): *δ* (ppm): 10.29–10.25 (d, *J* = 9.9 Hz, 2H), 7.77–7.67 (m, 3H), 7.47–7.19 (m, 8H), 6.66–6.61 (d, *J* = 15.6 Hz, 1H), 2.56–2.55 (m, 1H), 1.81–1.31 (m, 8H), 0.99–0.92 (m, 1H). ^13^C NMR spectrum, *δ*_c_, ppm, DMSO-*d*_6_: 171.27, 162.89, 140, 137.89, 134.09, 133.23, 131.42, 128.4, 128.12, 127.73, 126.7, 124.26, 122.57, 55.06, 42.38, 30.77, 30.23, 24.64, 24.45. HRMS (ESI) *m*/*z* calcd. For [C_22_H_23_BrN_2_O_2_]^+^: 427.34 [M + H]^+^, found 429.6.

##### 4.2.4.3 3-(3-Bromophenyl)-*N*′-(2-cyclopentyl-2-phenylacetyl)acrylohydrazide (6c)

Off-white solid, 156 mg, 80% yield, IR (KBr, cm^−1^): 3180, 3030, 2948, 2858, 1661, 1640, 1588, 1464, 1368, 1270, 1200, 1180, 1071, 1032, 966, 854, 784, 719, 698, 663, 552. ^1^H NMR (300 MHz, DMSO-*d*_6_): *δ* (ppm): 10.29 (s, 1H), 10.16 (s, 1H), 7.78 (s, 1H), 7.6–7.55 (dd, *J* = 8.1 Hz, 2H), 7.49–7.09 (m, 7H), 6.7–6.65 (d, *J* = 15.9 Hz, 1H), 2.56–2.5 (m, 1H), 1.76–1.3 (m, 8H), 0.96–0.94 (m, 1H). ^13^C NMR spectrum, *δ*_c_, ppm, DMSO-*d*_6_: 171.19, 168.93, 163.02, 156.08, 140.55, 140.03, 138.38, 137.14, 132.26, 131.06, 130.25, 128.4, 128.11, 128.03, 127.96, 126.68, 126.54, 126.42, 122.26, 121.08, 55.15, 55.02, 51.94, 42.50, 42.41, 30.75, 30.28, 30.23, 30.15, 24.87, 24.64, 24.45. HRMS (ESI) *m*/*z* calcd. For [C_22_H_23_BrN_2_O_2_]^+^: 427.34 [M + H]^+^, found 425.1.

##### 4.2.4.4 3-(4-Bromophenyl)-*N*′-(2-cyclopentyl-2-phenylacetyl)acrylohydrazide (6d)

Off-white solid, 147 mg, 75% yield, IR (KBr, cm^−1^): 3192, 3025, 2946, 2864, 1637, 1591, 1488, 1461, 1322, 1202, 1181, 1073, 1011, 972, 859, 815, 743, 697, 645, 558, 527. ^1^H NMR (300 MHz, DMSO-*d*_6_): *δ* (ppm): 10.26–10.16 (d, *J* = 29.7 Hz, 2H), 7.63–7.05 (m, 10H), 6.67–6.62 (d, *J* = 15.6 Hz, 1H), 2.58–2.49 (m, 1H), 1.81–1.30 (m, 8H), 1.01–0.92 (m, 1H). ^13^C NMR spectrum, *δ*_c_, ppm, DMSO-*d*_6_: 171.24, 163.23, 140.04, 138.75, 133.86, 131.94, 129.6, 128.11, 126.68, 122.95, 120.25, 55.03, 42.4, 30.75, 30.23, 24.65, 24.45. HRMS (ESI) *m*/*z* calcd. For [C_22_H_23_BrN_2_O_2_]^+^: 427.34 [M + H]^+^, found 429.6.

##### 4.2.4.5 *N*′-(2-Cyclopentyl-2-phenylacetyl)-3-(*p*-tolyl)acrylohydrazide (6e)

Off-white solid, 118 mg, 71% yield, IR (KBr, cm^−1^): 3193, 3024, 2945, 2864, 2819, 1693, 1635, 1590, 1512, 1456, 1386, 1203, 1180, 1144, 1110, 1034, 973, 861, 810, 771, 725, 697, 653, 561, 530.^1^H NMR (300 MHz, DMSO-*d*_6_): *δ* (ppm): 10.24 (b, 1H), 10.09 (b, 1H), 9.01 (m, 1H), 7.57–7.21 (m, 9H), 6.6–6.54 (d, *J* = 15.9 Hz, 1H), 3.32–3.2 (m, 1H), 2.33 (s, 3H), 1.82–1.31 (m, 8H), 0.98–0.96 (m, 1H). ^13^C NMR spectrum, *δ*_c_, ppm, DMSO-*d*_6_: 171.28, 163.64, 140.07, 139.97, 139.58, 131.83, 129.57, 128.1, 127.62, 126.66, 118.32, 55.05, 45.14, 44.78, 42.41, 30.76, 30.23, 24.64, 24.45, 20.95. HRMS (ESI) *m*/*z* calcd. For [C_23_H_26_N_2_O_2_]^+^: 362.47 [M + H]^+^, found 321.

##### 4.2.4.6 3-(4-Fluoro)-*N*′-(2-cyclopentyl-2-phenylacetyl)acrylohydrazide (6f)

Off-white solid, 139 mg, 83% yield, IR (KBr, cm^−1^): 3747, 3196, 3026, 2951, 2864, 1639, 1589, 1507, 1457, 1327, 1229, 1199, 1179, 1157, 1096, 1032, 967, 871, 827, 779, 726, 696, 638, 530.^1^H NMR (500 MHz, DMSO-*d*_6_): *δ* (ppm): 10.26 (b, 1H), 10.13 (b, 1H), 7.66–7.65 (m, 2H), 7.39–7.41 (dd, 2H), 7.53–7.50 (d, *J* = 15 Hz, 1H), 7.16–7.32 (m, 5H), 6.61–6.55 (d, *J* = 15.9 Hz, 1H), 3.31–3.33 (d, *J* = 10 Hz, 1H), 2.5–2.51 (m, 1H), 1.33–1.82 (m, 7H), 0.95–0.99 (m, 1H). ^13^C NMR spectrum, *δ*_c_, ppm, DMSO-*d*_6_: 171.73, 162.31–164.3, 163.89, 140.54, 139.29, 131.71, 130.36, 128.6, 128.57, 127.14, 116.51, 119.78, 55.53, 42.89, 31.24, 30.71, 25.12, 24.93. HRMS (ESI) *m*/*z* calcd. For [C_23_H_23_FN_2_O_2_]^+^: 366.44 [M + H]^+^, found 367.2.

##### 4.2.4.7 3-(3-Nitrophenyl)-*N*′-(2-cyclopentyl-2-phenylacetyl)acrylohydrazide (6g)

Pale yellow solid, 140 mg, 78% yield, IR (KBr, cm^−1^): 3747, 3167, 3028, 2952, 2866, 1642, 1590, 1530, 1468, 1346, 1267, 1206, 1184, 1074, 1031, 973, 902, 861, 834, 805, 751, 732, 712, 654, 562, 545, 517. ^1^H NMR (500 MHz, DMSO-*d*_6_): *δ* (ppm): 10.33 (S, 1H), 10.24 (s, 1H), 8.4 (s, 1H), 8.23–8.21 (d, *J* = 8 Hz, 1H), 8.04–8.02 (d, *J* = 8.5 Hz, 1H), 7.73–7.72 (t, *J* = 8 Hz, 1H), 7.65–7.62 (d, *J* = 16 Hz, 1H), 7.38–7.21 (m, 5H), 6.84–6.81 (d, *J* = 15.5 Hz, 1H), 2.57–2.53 (m, 1H), 1.80–1.3 (m, 8H), 0.99–0.94 (m, 1H). ^13^C NMR spectrum, *δ*_c_, ppm, DMSO-*d*_6_: 171.16, 162.77, 148.3, 140.02, 137.73, 136.45, 133.82, 130.52, 128.12, 126.7, 124.01, 122.32, 121.91, 55.02, 42.43, 30.76, 30.23, 24.64, 24.45. HRMS (ESI) *m*/*z* calcd. For [C_22_H_23_N_3_O_4_]^+^: 393.44 [M + H]^+^, found 394.4.

##### 4.2.4.8 *N*′-(2-cyclopentyl-2-(2,4,5-trifluorophenyl)acetyl)cinnamohydrazide (7a)

Off-white solid, 96 mg, 65% yield, IR (KBr, cm^−1^): 3747, 3184, 2953, 2869, 1682, 1642, 1610, 1513, 1423, 1335, 1257, 1207, 1185, 1148, 976, 878, 857, 761, 708, 615, 518. ^1^H NMR (500 MHz, DMSO-*d*_6_): *δ* (ppm): 10.36 (s, 1H), 10.13 (s, 1H), 7.67–7.62 (m, 1H), 7.59–7.49 (m, 4H), 7.44–7.40 (m, 3H), 6.65–6.62 (d, *J* = 15.5 Hz, 1H), 3.68–3.66 (d, *J* = 11 Hz, 1H), 2.44–2.42 (d, *J* = 10.5 Hz, 1H), 1.83–1.79 (m, 1H), 1.67–1.65 (m 1H), 1.58–1.49 (m, 6H), 0.99–0.91 (m, 1H). ^19^F NMR (500 MHz, DMSO-*d*_6_): *δ* (ppm): −118.79, −136.45, −142.94. ^13^C NMR spectrum, *δ*_c_, ppm, DMSO-*d*_6_: 170.04, 163.68, 140.26, 134.51, 129.82, 128.99, 127.68, 119.25, 117.15, 105.7, 45.47, 43, 30.52, 29.99, 24.48, 24.27. HRMS (ESI) *m*/*z* calcd. For [C_22_H_21_F_3_N_2_O_2_]^+^: 402.2 [M + H]^+^, found 403.1.

##### 4.2.4.9 *N*′-(2-Cyclopentyl-2-(2,4,5-trifluorophenyl)acetyl)-3-(*p*-tolyl)acrylohydrazide (7b)

Off-white solid, 104 mg, 68% yield, IR (KBr, cm^−1^): 3186, 3029, 2948, 2867, 1666, 1632, 1587, 1513, 1461, 1424, 1330, 1255, 1197, 1184, 1156, 973, 884, 862, 807, 758, 709, 646, 524, 514. ^1^H NMR (500 MHz, DMSO-*d*_6_): *δ* (ppm): 10.34 (s, 1H), 10.07 (s, 1H), 7.67–7.61 (m, 1H), 7.55–7.45 (m, 4H), 7.24–7.22 (d, *J* = 7.5 Hz, 1H), 6.58–6.55 (dd, *J* = 15.5 Hz, 1H), 3.67–3.65 (d, *J* = 2 Hz, 11H), 2.44–2.42 (m, 1H), 2.32 (s, 3H), 1.81–1.78 (m, 1H), 1.67–1.65 (m, 1H), 1.58–1.49 (m, 6H), 0.99–0.91 (m, 1H). ^19^F NMR (500 MHz, DMSO-*d*_6_): *δ* (ppm): 118.79, 136.46, 142.95.^13^C NMR spectrum, *δ*_c_, ppm, DMSO-*d*_6_: 170.04, 163.5, 156.04, 140.2, 139.56, 131.78, 129.56, 127.65, 123.72, 118.16, 114.75, 105.67, 45.45, 43.02, 30.52, 24.47, 22.2. HRMS (ESI) *m*/*z* calcd. For [C_23_H_23_ F_3_N_2_O_2_]^+^: 416.44 [M + H]^+^, found 417.2.

##### 4.2.4.10 3-(4-Bromophenyl)-*N*′-(2-cyclopentyl-2-(2,4,5-trifluorophenyl)acetyl)acrylohydrazide (7c)

Off-white solid, 131 mg, 74% yield, IR (KBr, cm^−1^): 3747, 3176, 3030, 2945, 2866, 1633, 1587, 1514, 1488, 1463, 1424, 1330, 1256, 1200, 1157, 1074, 1012, 971, 884, 863, 814, 771, 709, 695, 646, 518. ^1^H NMR (500 MHz, DMSO-*d*_6_): *δ* (ppm): 10.38 (s, 1H), 10.16 (s, 1H), 7.63–7.61 (m, 3H), 7.54–7.53 (m, 4H), 6.66–6.63 (d, *J* = 16 Hz, 1H), 3.68–3.65 (d, *J* = 10.5 Hz, 1H), 2.49–2.1 (m, 1H), 1.9–1.78 (m, 1H), 1.68–1.64 (m, 1H), 1.58–1.45 (m, 6H), 0.99–0.91 (m, 1H). ^19^F NMR (500 MHz, DMSO-*d*_6_): *δ* (ppm): 118.78, 136.42, 142.93. ^13^C NMR spectrum, *δ*_c_, ppm, DMSO-*d*_6_: 170.02, 163.46, 139.01, 133.81, 131.94, 129.64, 123.02, 120.09, 117.11, 105.46, 45.47, 43, 30.52, 29.98, 24.48, 24.27. HRMS (ESI) *m*/*z* calcd. For [C_22_H_20_Br F_3_N_2_O_2_]^+^: 481.31 [M + H]^+^, found 478.9.

##### 4.2.4.11 3-(3-Chlorophenyl)-*N*′-(2-cyclopentyl-2-(2,4,5-trifluorophenyl)acetyl)acrylohydrazide (7d)

Off-white solid, 128 mg, 80% yield, IR (KBr, cm^−1^): 3747, 3186, 2951, 2869, 1685, 1590, 1514, 1475, 1423, 1331, 1256, 1203, 1150, 1078, 971, 879, 851, 784, 703, 683, 640, 523. ^1^H NMR (500 MHz, DMSO-*d*_6_): *δ* (ppm): 10.39 (s, 1H), 10.15 (s, 1H), 7.67–7.61 (m, 2H), 7.56–7.45 (m, 5H), 6.7–6.67 (d, *J* = 16 Hz, 1H), 3.68–3.66 (d, *J* = 11 Hz, 1H), 2.49–2.41 (m, 1H), 1.82–1.78 (m, 1H), 1.68–1.64 (m, 1H), 1.58–1.49 (s, 6H), 0.99–0.96 (m, 1H). ^19^F NMR (500 MHz, DMSO-*d*_6_): *δ* (ppm): 118.79, 136.42, 142.92. ^13^C NMR spectrum, *δ*_c_, ppm, DMSO-*d*_6_: 169.99, 163.31, 138.72, 136.83, 133.7, 130.81, 129.43, 127.44, 126.11, 120.95, 105.3, 45.46, 42.99, 30.52, 29.99, 24.48, 24.27. HRMS (ESI) *m*/*z* calcd. For [C_22_H_20_Cl F_3_N_3_O_2_]^+^: 436.86 [M + H]^+^, found 437.1.

##### 4.2.4.12 *N*′-(2-Cyclopentyl-2-(2,4,5-trifluorophenyl)acetyl)-3-(4-methoxyphenyl)acrylohydrazide (7e)

Off-white solid, 141 mg, 89% yield, IR (KBr, cm^−1^): 3747, 3201, 3025, 2954, 2868, 2838, 1667, 1630, 1583, 1512, 1458, 1423, 1331, 1306, 1287, 1259, 1196, 1174, 1157, 1110, 1030, 973, 884, 864, 830, 809, 783, 757, 698, 645, 550, 525. ^1^H NMR (500 MHz, DMSO-*d*_6_): *δ* (ppm): 10.31 (s, 1H), 10.01 (s, 1H), 7.67–7.61 (m, 1H), 7.54–7.43 (m, 4H), 6.99–6.97 (d, *J* = 8.5 Hz, 1H), 6.49–6.46 (d, *J* = 16 Hz, 1H), 3.8 (s, 3H), 3.67–3.63 (d, *J* = 11 Hz, 1H), 2.49–2.44 (m, 1H), 1.83–1.77 (m, 1H), 1.69–1.64 (m, 1H), 1.59–1.4 (m, 6H), 0.99–0.95 (m, 1H). ^19^F NMR (500 MHz, DMSO-*d*_6_): *δ* (ppm): 118.8, 136.48, 142.96. ^13^C NMR spectrum, *δ*_c_, ppm, DMSO-*d*_6_: 170.06, 164.05, 160.6, 139.97, 129.32, 127.09, 116.66, 114.43, 55.28, 45.46, 43.01, 30.52, 29.98, 24.48, 24.27. HRMS (ESI) *m*/*z* calcd. For [C_23_H_23_F_3_N_2_O_3_]^+^: 432.44 [M + H]^−^, found 433.1.

##### 4.2.4.13 3-(3-Bromophenyl)-*N*′-(2-cyclopentyl-2-(2,4,5-trifluorophenyl)acetyl)acrylohydrazide (7f)

Off-white solid, 152 mg, 86% yield, IR (KBr, cm^−1^): 3747, 3178, 3034, 2962, 2869, 1646, 1598, 1513, 1472, 1424, 1335, 1258, 1204, 1157, 1072, 970, 890, 860, 844, 809, 781, 741, 719, 697, 663, 642, 603, 522. ^1^H NMR (500 MHz, DMSO-*d*_6_): *δ* (ppm): 10.39 (s, 1H), 10.14 (s, 1H), 7.79–7.67 (t, *J* = 1.5 Hz, 1H), 7.65–7.57 (m, 3H), 7.53–7.4 (m, 2H), 7.38–7.32 (t, *J* = 8 Hz, 1H), 6.69–6.66 (m, *J* = 16 Hz, 1H), 3.68–3.66 (d, *J* = 11 Hz, 1H), 2.49–2.44 (m, 1H), 1.81–1.78 (m, 1H), 1.67–1.66 (m, 1H), 1.58–1.44 (m, 6H), 0.99–0.96 (m, 1H). ^19^F NMR (500 MHz, DMSO-*d*_6_): *δ* (ppm): 118.79, 136.42, 142.92. ^13^C NMR spectrum, *δ*_c_, ppm, DMSO-*d*_6_: 169.98, 163.28, 138.64, 137.09, 132.33, 131.07, 130.3, 126.47, 122.27, 120.93, 105.54, 45.46, 42.99, 30.52, 29.99, 24.48, 24.27. HRMS (ESI) *m*/*z* calcd. For [C_22_H_20_BrF_3_N_2_O_2_]^+^: 481.31 [M + H]^+^, found 481.2.

##### 4.2.4.14 3-(3-Nitrophenyl)-*N*′-(2-cyclopentyl-2-(2,4,5-trifluorophenyl)acetyl)acrylohydrazide (7g)

Brown colored solid, 145 mg, 89% yield, IR (KBr, cm^−1^): 3198, 3073, 2948, 2865, 1641, 1586, 1530, 1462, 1426, 1354, 1253, 1205, 1157, 1099, 961, 886, 856, 808, 775, 751, 718, 700, 660, 634, 603, 523. ^1^H NMR (500 MHz, DMSO-*d*_6_): *δ* (ppm): 10.43 (s, 1H), 10.22 (s, 1H), 8.41 (d, *J* = 2 Hz, 1H), 8.23–8.22 (dd, *J* = 1.5, 1 Hz, 1H), 8.21 (d, *J* = 0.5 Hz, 1H), 7.73–7.7 (t, *J* = 8 Hz, 1H), 7.66–7.63 (m, 2H), 7.53–7.5 (m, 1H), 6.84–6.81 (d, *J* = 16 Hz, 1H), 3.69–3.67 (d, *J* = 11 Hz, 1H), 2.49–2.43 (m, 1H), 1.86–1.8 (m, 1H), 1.67–1.65 (m, 1H), 1.58–1.4 (m, 6H), 0.99–0.96 (m, 1H). ^19^F NMR (500 MHz, DMSO-*d*_6_): *δ* (ppm): 118.79, 136.41, 142.92. ^13^C NMR spectrum, *δ*_c_, ppm, DMSO-*d*_6_: 169.93, 163.01, 148.29, 146.93, 137.96, 136.4, 133.83, 130.5, 123.79, 122.18, 121.95, 117.25, 105.69, 45.73, 43.11, 30.51, 24.88. HRMS (ESI) *m*/*z* calcd. For [C_22_H_20_F_3_N_3_O_4_]^+^: 447.41 [M + H]^+^, found 445.9.

### 4.3. α-Glucosidase inhibitory assay

The test compounds were evaluated for their α-glucosidase inhibitory activity following the methodology outlined by Pistia *et al.*^62^ and Bhatia *et al.*^63^ The test compounds and Acarbose were both dissolved in phosphate buffer at a pH of 6.8. The compounds at varied concentrations ranging from 50 to 200 μg mL^−1^ were combined with 50 μL of α-glucosidase (maltase) enzyme solution (1 U mL^−1^) from Yeast (Sisco Research Laboratories Pvt. Ltd). The mixer was placed at a temperature of 37 °C for duration of 15 minutes, after the addition of 0.1 M phosphate buffer (pH 6.8). Afterward, 25 μl of substrate buffer was introduced into the system to initiate the reaction, and the incubation was prolonged at a temperature of 37 °C for a length of 15 minutes. Finally, the reaction was stopped by adding 50 μl of a 0.2 M sodium carbonate solution, thereby terminating the processes. The measurement was determined at a wavelength of 450 nm. Acarbose was used as a standard chemical and given at various doses ranging from 50 to 200 μg mL^−1^. The measurement of enzyme activity was carried out as follows.

The formula for calculating the percentage is (OD_blank_ − OD_sample_) divided by OD_blank_, multiplied by 100.

A unit of enzyme may be accurately defined as the amount of α-glucosidase enzyme required to generate one micromole of *p*-nitrophenol (product) from *p*-nitrophenyl-α-d-glucopyranoside (substrate) for a time of one minute. The IC_50_, representing the concentration required to inhibit 50% of the enzyme activity, was determined by fitting a regression equation to a graph that plotted concentration (varying from 50–200 μg mL^−1^) on the *X*-axis and percentage of inhibition on the *Y*-axis for various fractions and extracts.

### 4.4. Molecular docking

To ascertain the most effective way of binding a ligand to a target protein, molecular docking is utilized. When the ligand forms a stable combination with other potential ligands, this is very beneficial. A highly effective technique for examining the molecular interactions between ligands and proteins is docking.

#### 4.4.1 UCSF chimera

Structural biologists, medicinal researchers, and experts in the fields of bioinformatics and drug development regularly use the UCSF chimera programme to further enhance their understanding of molecular structure and function. A flexible software suite called chimera makes it possible to interactively explore and visualize molecular structures. It can help users comprehend pertinent data better, including conformational ensembles, supra-molecular assemblies, trajectories, density maps, and sequence alignments.

#### 4.4.2 AutoDock Vina

The automated character of the AutoDock Vina molecular docking software is evident. The three-dimensional structure of the target protein exhibits enhanced binding of the ligand. Computational docking approaches, such as protein-ligand docking, blind docking, and site-specific docking, are performed using the reputable bioinformatics software AutoDock Vina. Along with docking, AutoDock Vina, sometimes called AutoDock tools, helps modify the structure of ligands and proteins. This method makes it possible to evaluate molecular assemblies of different sizes. Alpha-glucosidase's crystal structure (PDB ID:3WY1) was obtained from the RCSB PDB database. The data supplied includes the number of hydrogen bonds (H-bonds) at position 44, binding energy, and root mean square deviation (RMSD). The protein target's docking location was determined to be a grid box with dimensions of 21 × 21 × 21 and parameters of *X*: −9.1, *Y*: −7.4, *Z*: 24.2 Å. Inside this grid box, significant chemical substances for interaction were found.^25^

### 4.5. Swiss ADME

#### 4.5.1 Evaluation of physico-chemical properties

SMILES representations for every planned chemical have been created, and the process has been completed with success.^64^ SwissADME is an online tool that may be accessed at http://www.swissadme.ch/index.php. The compounds SMILES representations and corresponding chemical codes were entered into the tool. The programme has a “run” button function that helps with parameter calculating once the submission process is completed. An examination of the data was then performed on the collected results, which were available in both PDF and CSV formats.

## Data availability

All data generated or analyzed during this study are included in this published article and its ESI files.†

## Ethics approval

This article does not contain any studies with animals performed by any of the authors.

## Consent for publication

We authorize to publish the article without any conflict.

## Author contributions

Ram Reddy Mudireddy: investigation, formal analysis, methodology, and writing – original draft. Rambabu Gundla: project administration and supervision. Baji Baba Shaik: investigation, and software. Anoop Bodapati: validation, and resources. Panasa Mahesh: conceptualisation, and writing – original draft. Shiva Sravan Naidu: data curation, and funding acquisition. Damodar Tirumalasetti: formal analysis, and writing – review & editing. Naresh Kumar Katari: visualization and writing – review & editing.

## Conflicts of interest

The authors declare no conflict of interest regarding the publication of this manuscript.

## Supplementary Material

RA-015-D5RA01971K-s001
