# Supplementary material for: Synthesis, characterization, and in vitro and in silico α-glucosidase inhibitory evolution of novel N′-(2-cyclopentyl-2-phenylacetyl)cinnamohydrazide derivatives
Source: RSC Adv. 2025 May 21;15(22):17118–29. doi: 10.1039/d5ra01971k (PMC12093506; doi:10.1039/d5ra01971k)
Supplement: RA-015-D5RA01971K-s001 [file RA-015-D5RA01971K-s001.pdf]

Supplementary Information (SI) for RSC Advances.
This journal is © The Royal Society of Chemistry 2025

Supporting Information

Synthesis, Characterization, *In vitro* and *In silico* α-glucosidase inhibitory evolution of novel *N'*-(2-cyclopentyl-2-phenylacetyl)cinnamohydrazide derivatives

Table of contents

Table S1: ^{1}H, ^{13}C and 2D NMR data interpretation of **6f.**
Figure S1-S4: FT-IR, ^{1}H NMR, ^{13}C NMR, HRMS for compound **6a**
Figure S5-S8: FT-IR, ^{1}H NMR, ^{13}C NMR, HRMS for compound **6b**
Figure S9-S12: FT-IR, ^{1}H NMR, ^{13}C NMR, HRMS for compound **6c**
Figure S13-S16: FT-IR, ^{1}H NMR, ^{13}C NMR, HRMS for compound **6d**
Figure S17-S20: FT-IR, ^{1}H NMR, ^{13}C NMR, HRMS for compound **6e**
Figure S21-S26: FT-IR, ^{1}H NMR, APT, HRMS, HMBC and HSQC for compound **6f**
Figure S27-S30: FT-IR, ^{1}H NMR, ^{13}C NMR, HRMS for compound **6g**
Figure S31-S35: FT-IR, ^{1}H NMR, ^{13}C NMR, HRMS and ^{19}F NMR for compound **7a**
Figure S36-S40: FT-IR, ^{1}H NMR, ^{13}C NMR, HRMS and ^{19}F NMR for compound **7b**
Figure S41-S45: FT-IR, ^{1}H NMR, ^{13}C NMR, HRMS and ^{19}F NMR for compound **7c**
Figure S46-S50: FT-IR, ^{1}H NMR, ^{13}C NMR, HRMS and ^{19}F NMR for compound **7d**
Figure S51-S55: FT-IR, ^{1}H NMR, ^{13}C NMR, HRMS and ^{19}F NMR for compound **7e**
Figure S56-S60: FT-IR, ^{1}H NMR, ^{13}C NMR, HRMS and ^{19}F NMR for compound **7f**
Figure S61-S65: FT-IR, ^{1}H NMR, ^{13}C NMR, HRMS and ^{19}F NMR for compound **7g**
Figure S66: α-Glucosidase inhibitory effect of compounds 7b and 7d

List of abbreviations:

IR- Infrared spectroscopy

TMS- Tetramethylsilane

ITMS- Ion trap *mass spectrometry*

***N'*-(2-cyclopentyl-2-phenylacetyl)cinnamohydrazide (6a):**

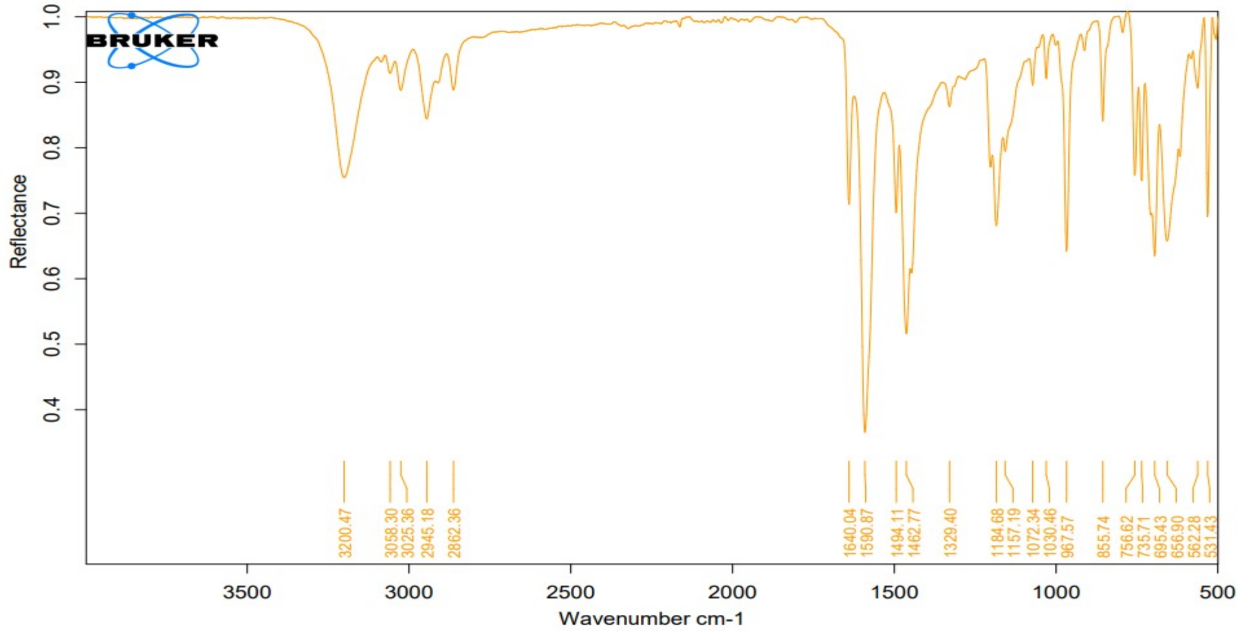

Figure S1: FT-IR spectrum of compound 6a

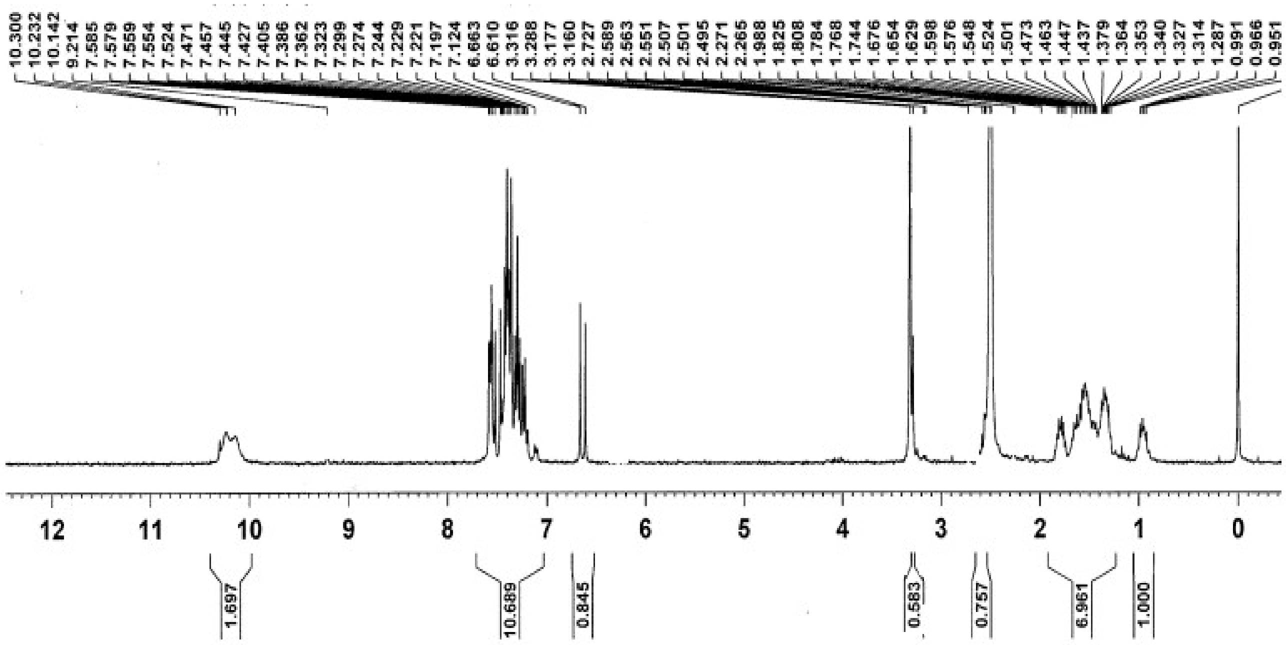

Figure S2: H[1] NMR spectrum of compound 6a

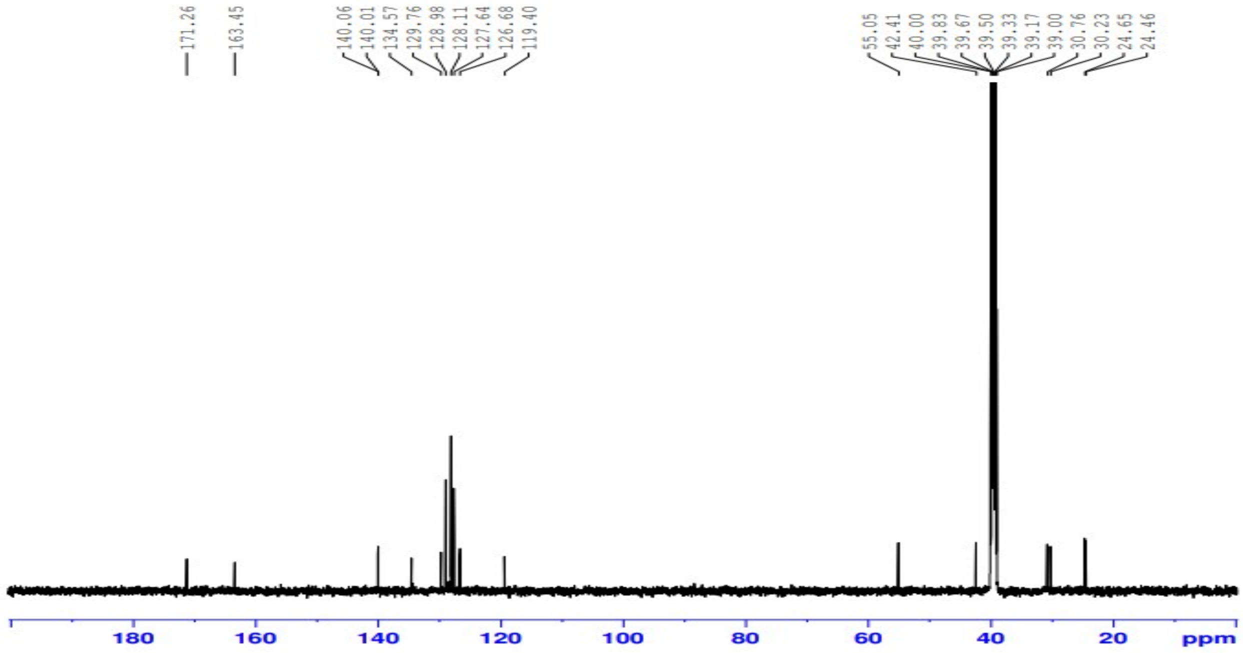

Figure S3: ^{13}C NMR spectrum (500 MHz, DMSO-d_6) of compound 6a

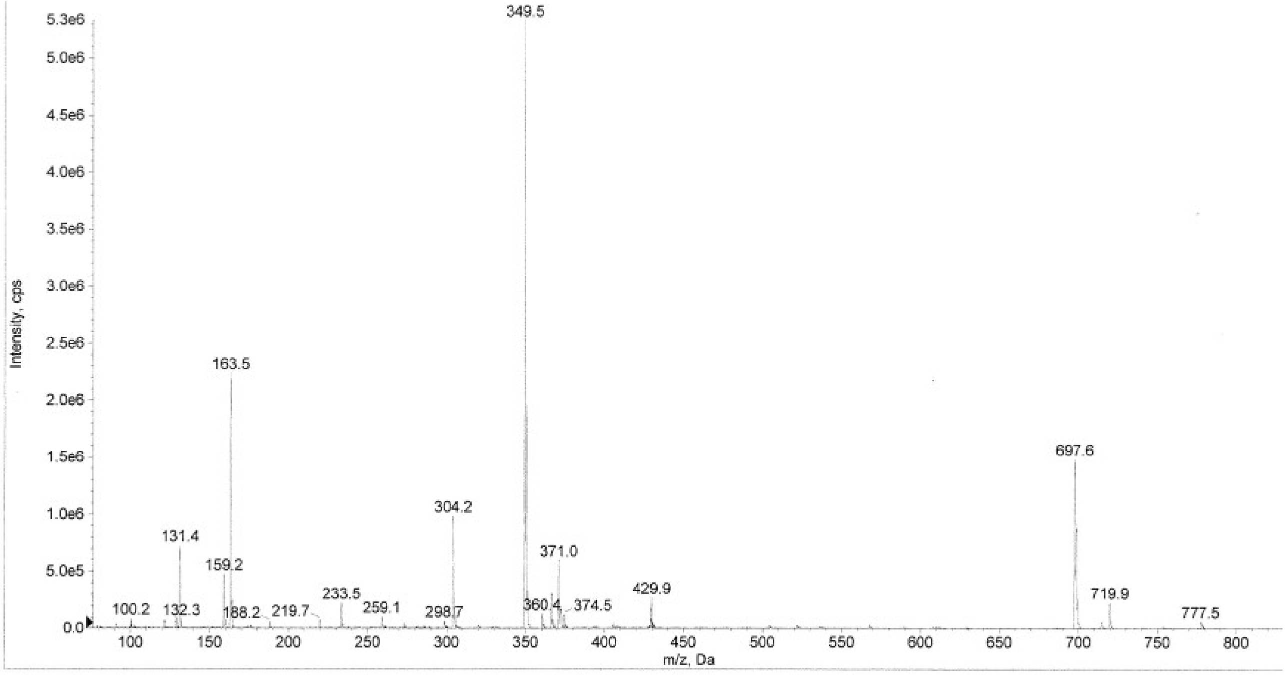

Figure S4: Mass spectrum of compound 6a

3-(2-bromophenyl)-*N’*-(2-cyclopentyl-2-phenylacetyl)acrylohydrazide (6b):

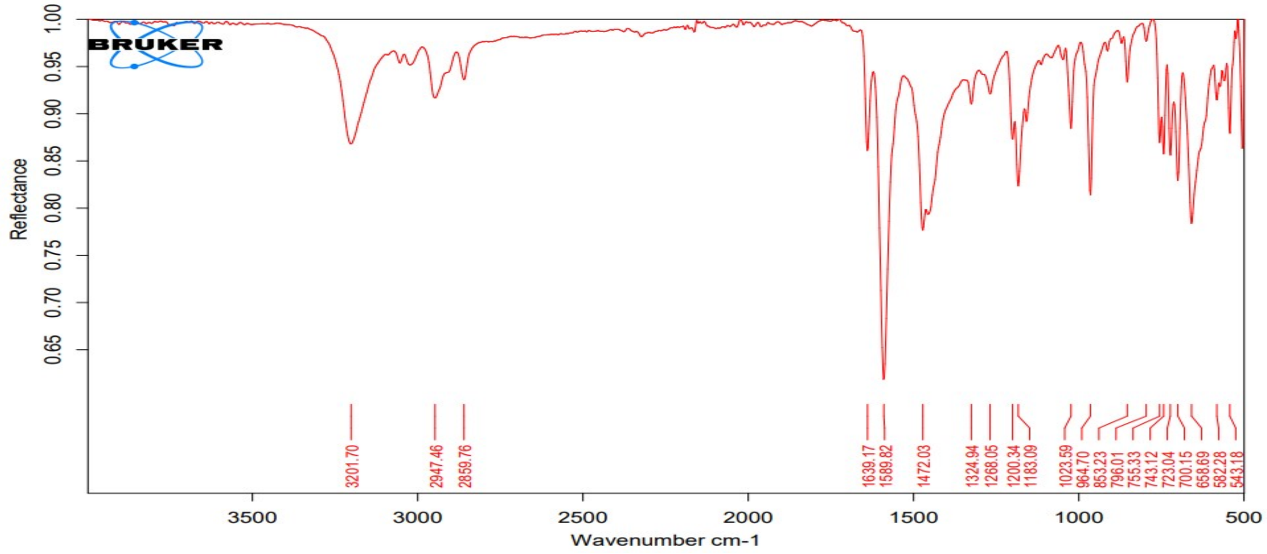

Figure S5: FT-IR spectrum of compound 6b

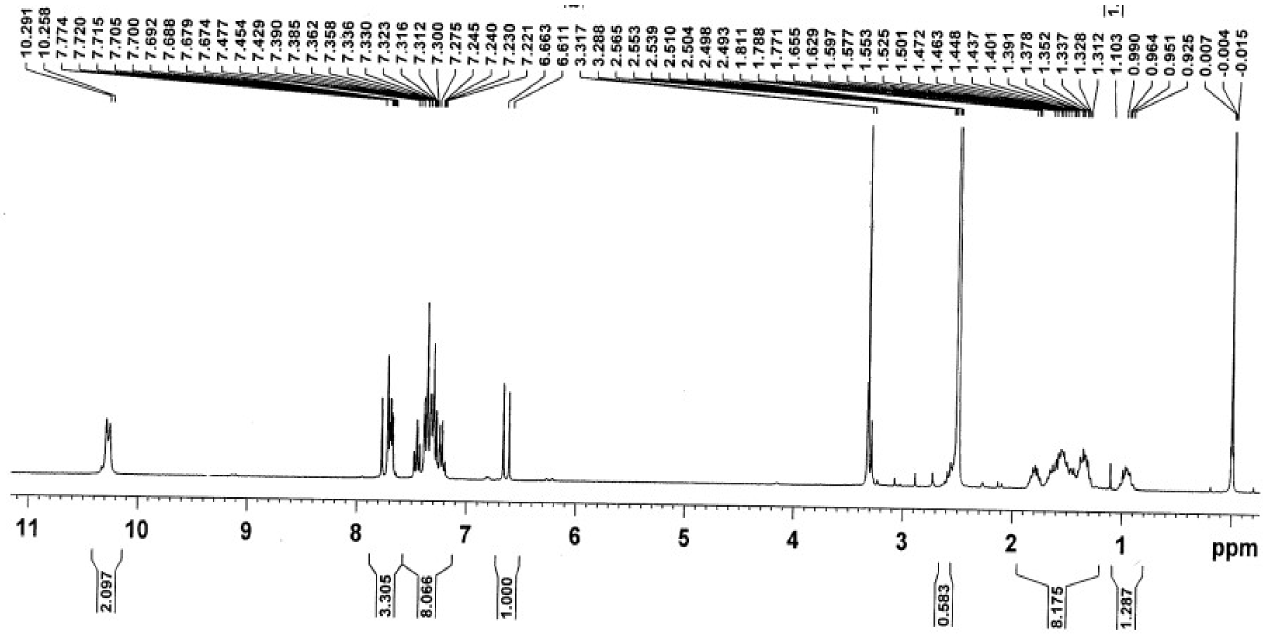

Figure S6: H[1] NMR spectrum of compound 6b

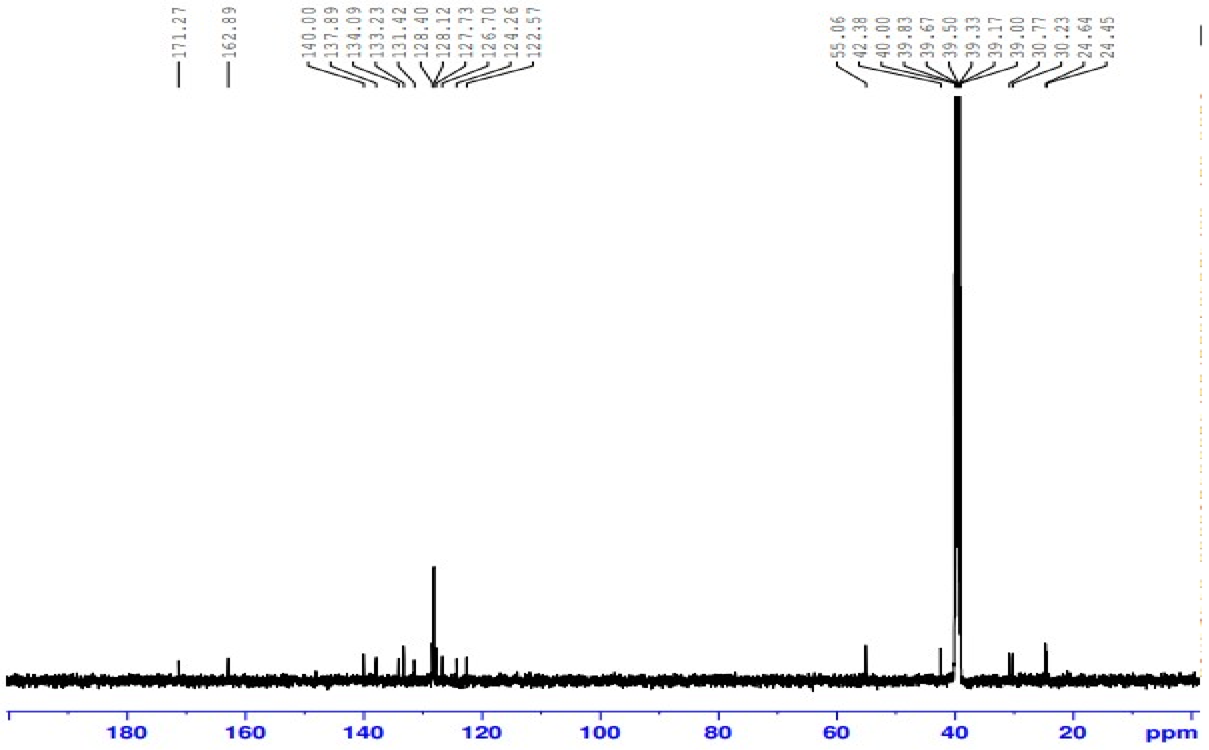

Figure S7: ^{13}C NMR spectrum (500 MHz, DMSO-d_6) of compound 6b

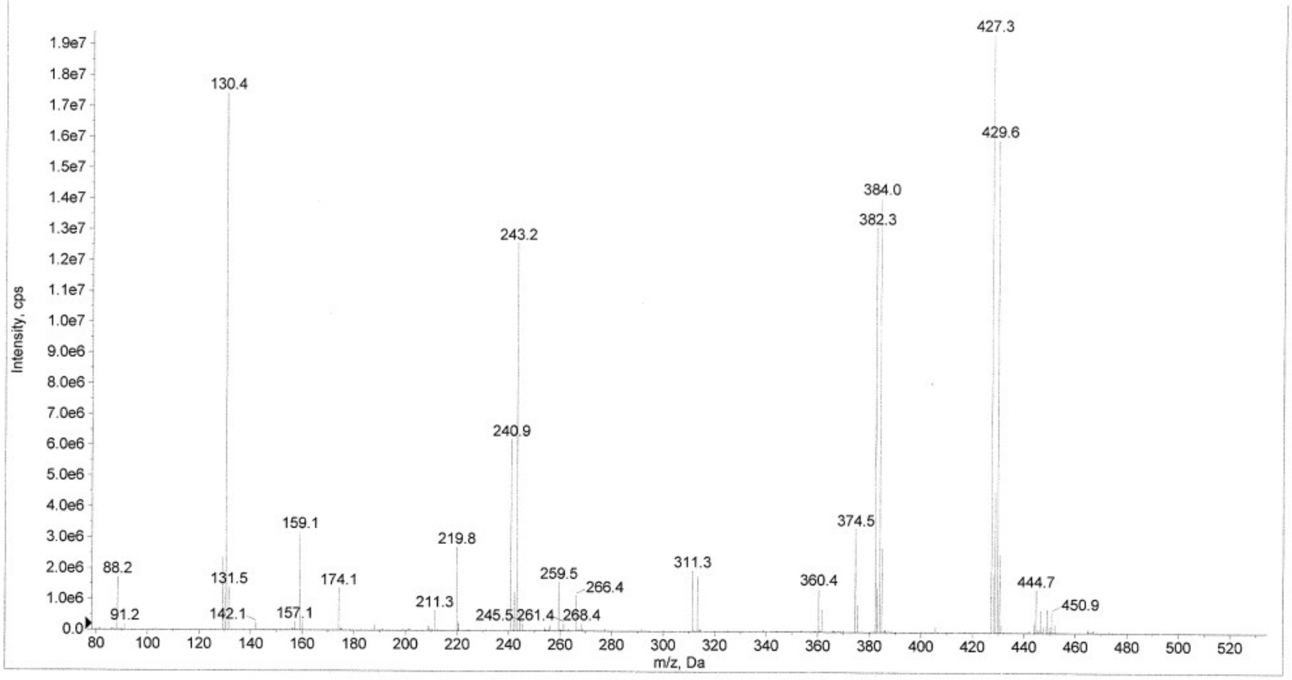

Figure S8: Mass spectrum of compound 6b

3-(3-bromophenyl)-*N'*-(2-cyclopentyl-2-phenylacetyl)acrylohydrazide (6c):

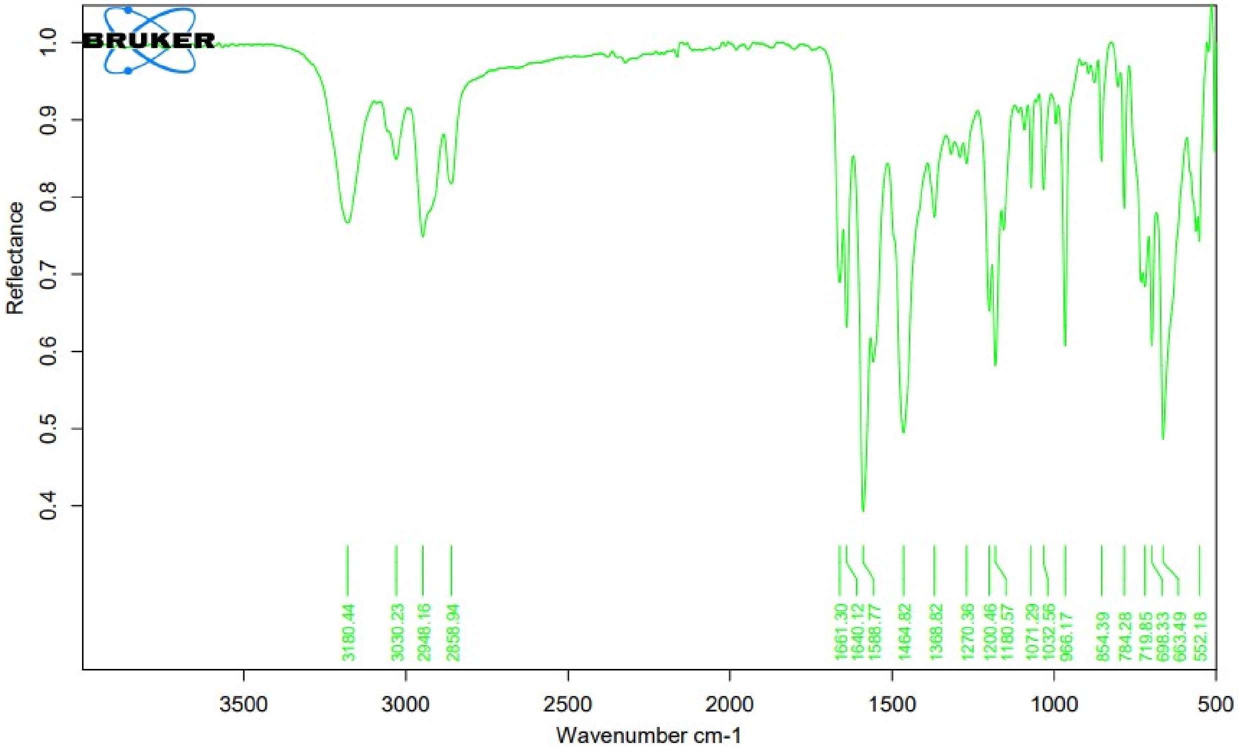

Figure S9: FT-IR spectrum of compound 6c

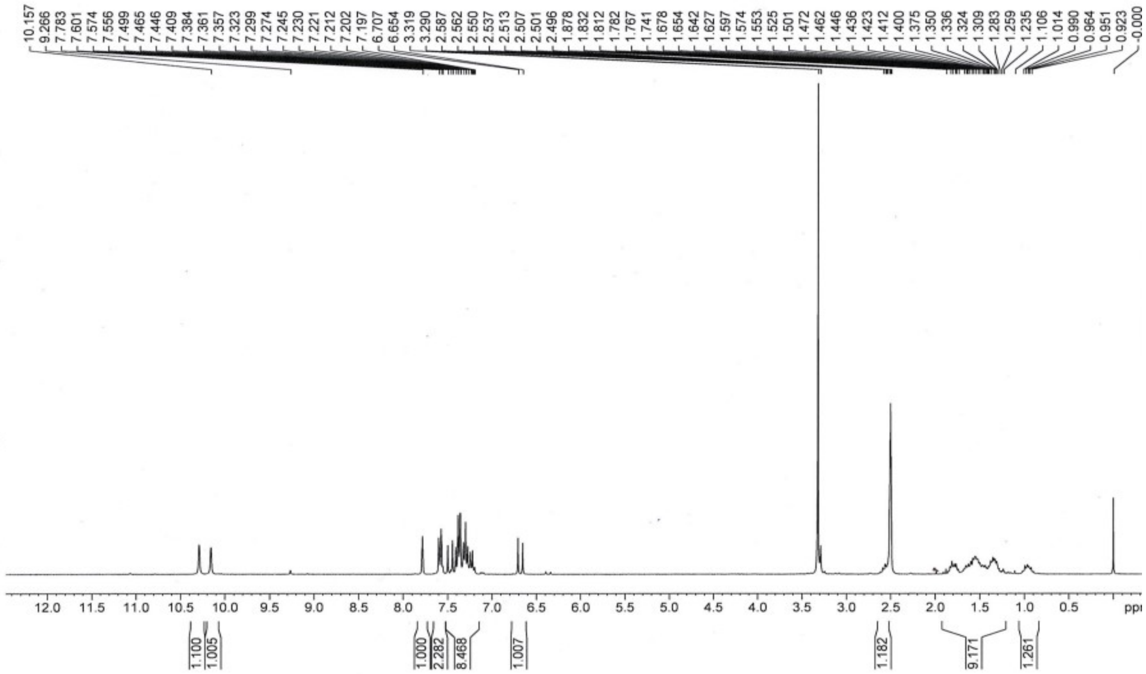

Figure S10: H¹ NMR spectrum of compound 6c

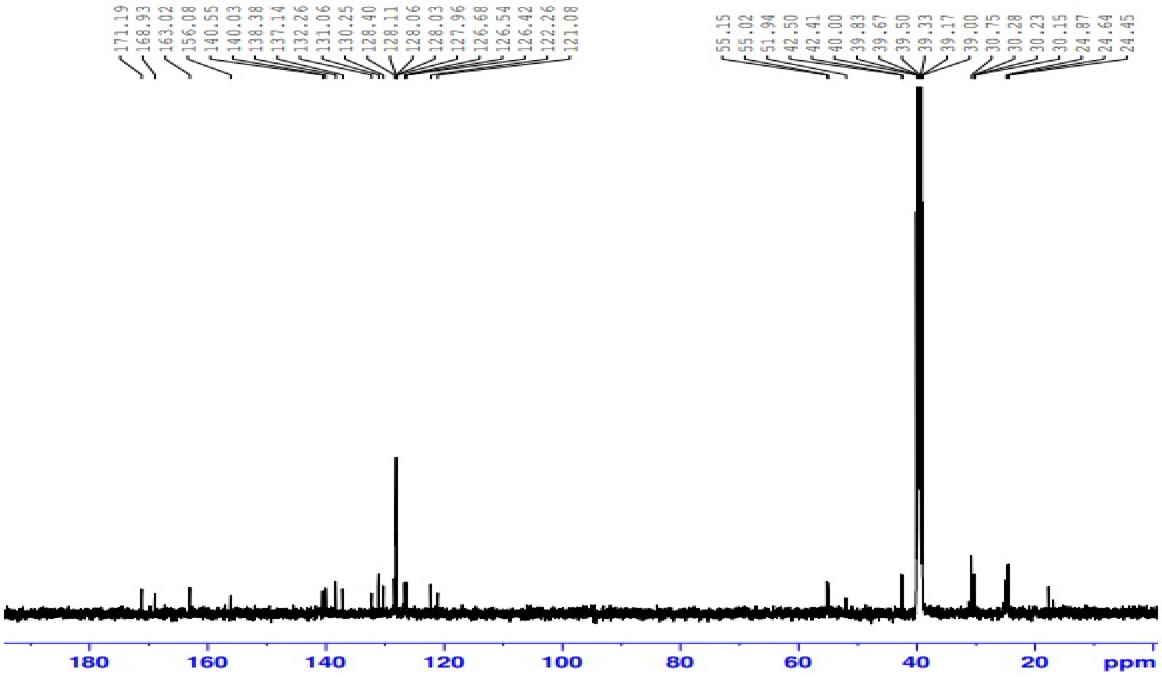

Figure S11: ^{13}C NMR spectrum (500 MHz, DMSO-d_6) of compound 6c

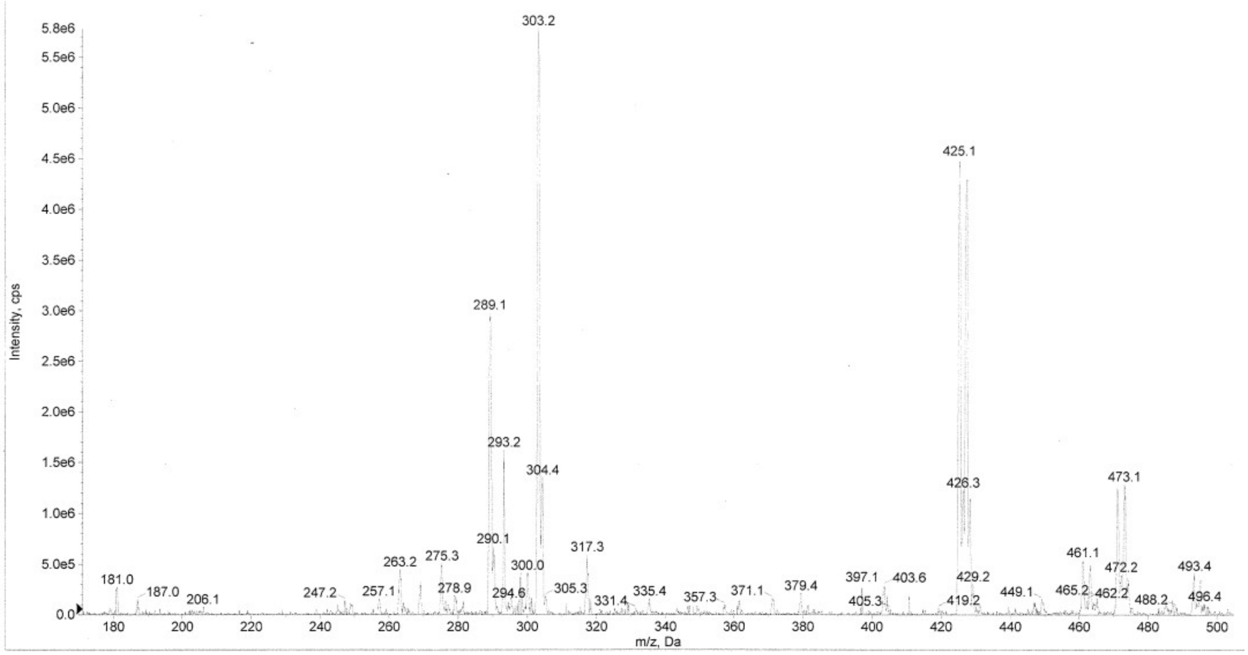

Figure S12: Mass spectrum of compound 6c

3-(4-bromophenyl)-*N'*-(2-cyclopentyl-2-phenylacetyl)acrylohydrazide (6d):

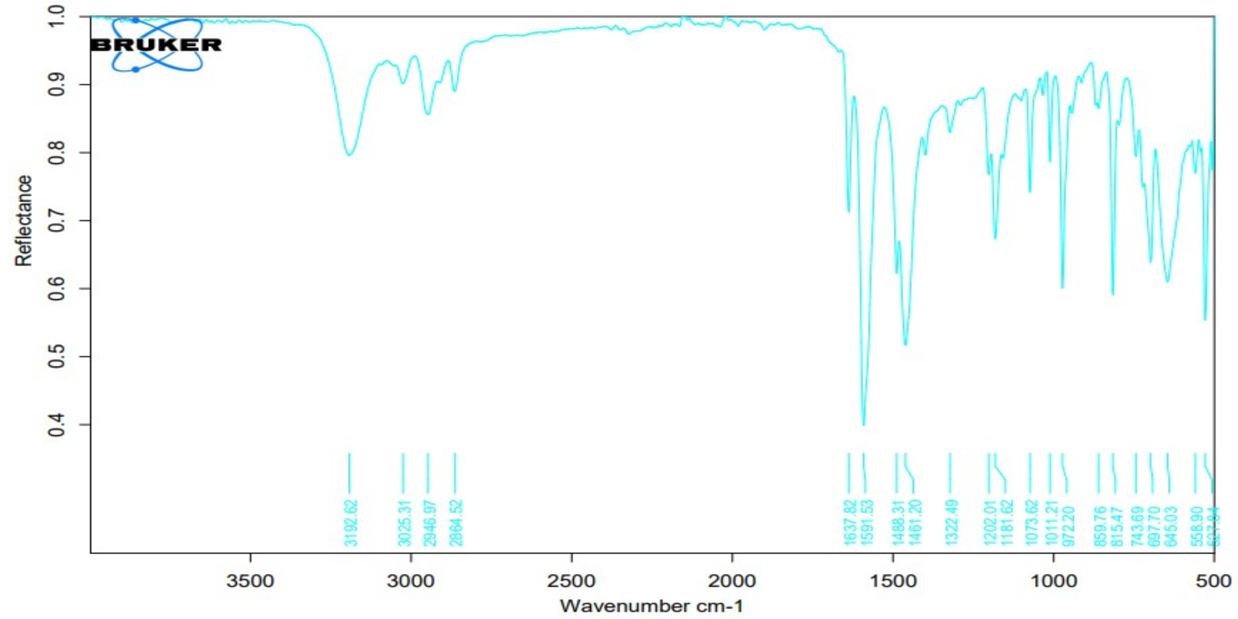

Figure S13: FT-IR spectrum of compound 6d

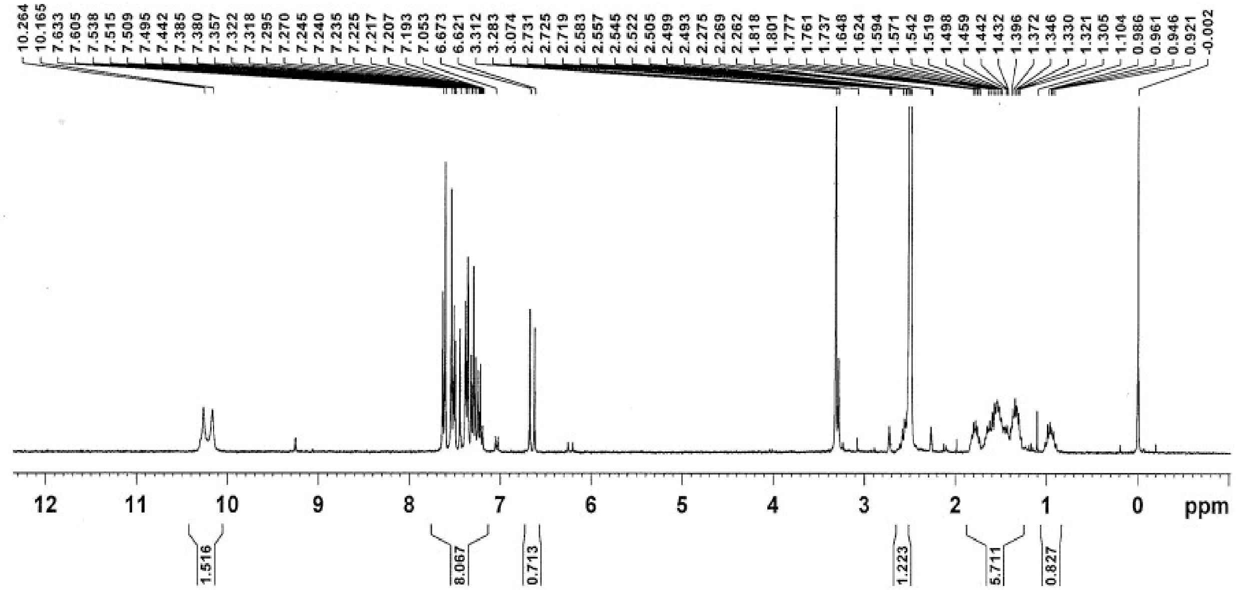

Figure S14: H[1] NMR spectrum of compound 6d

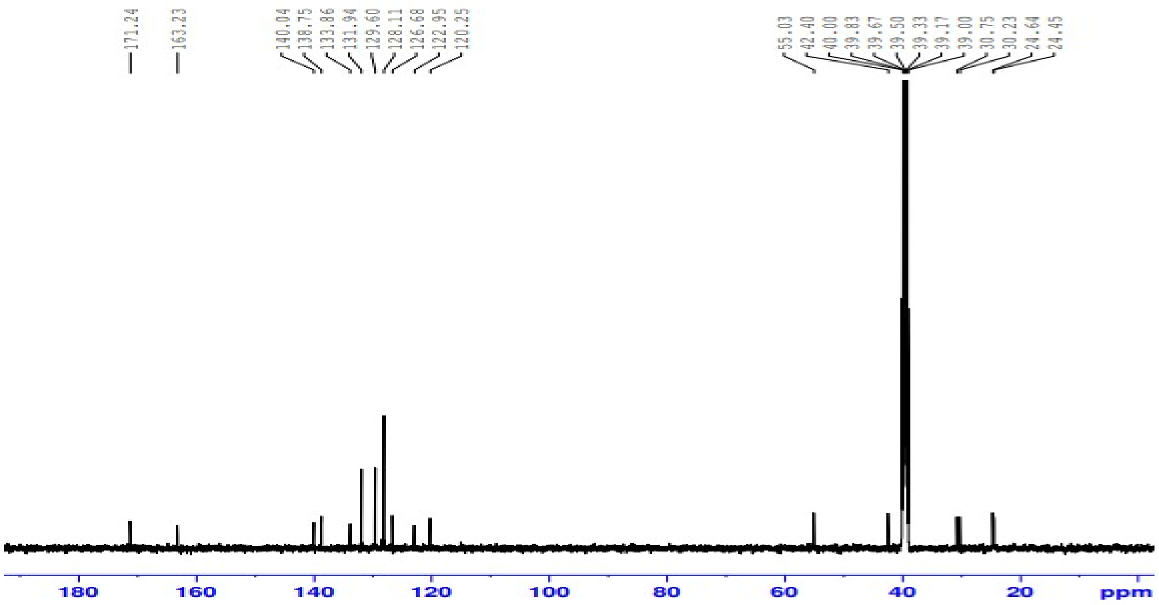

Figure S15: ^{13}C NMR spectrum (500 MHz, DMSO-d_6) of compound 6d

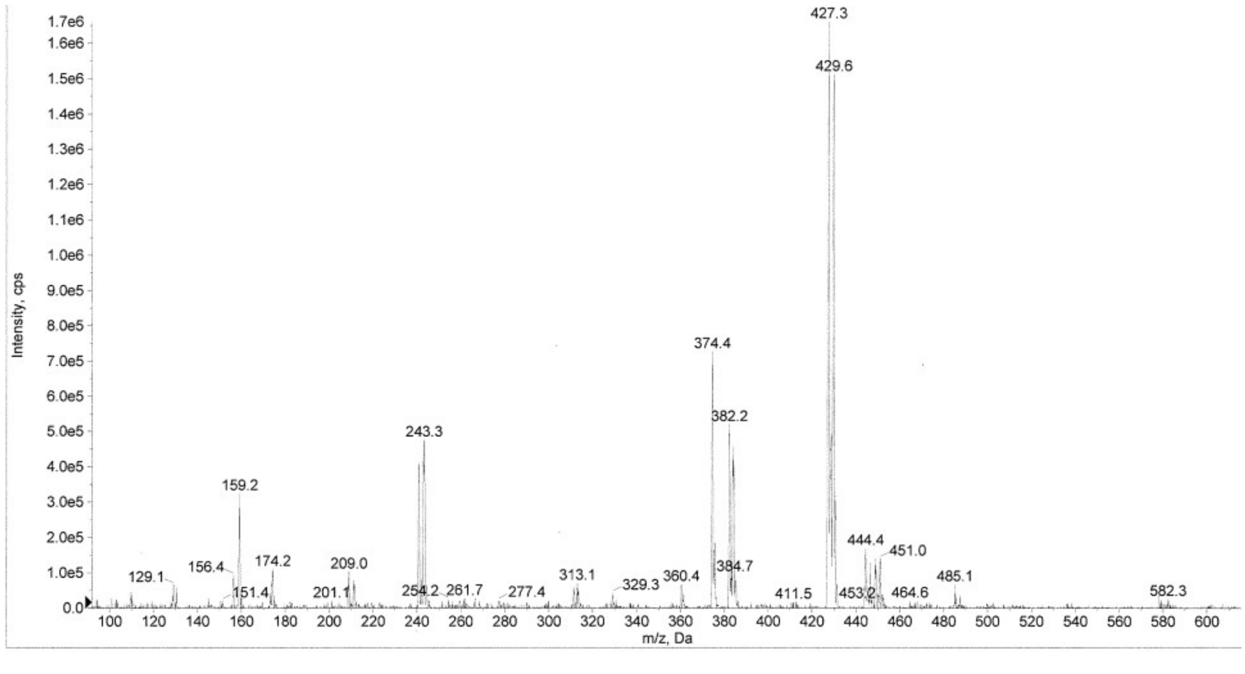

Figure S16: Mass spectrum of compound 6d

***N'*-(2-cyclopentyl-2-phenylacetyl)-3-(p-tolyl)acrylohydrazide (6e):**

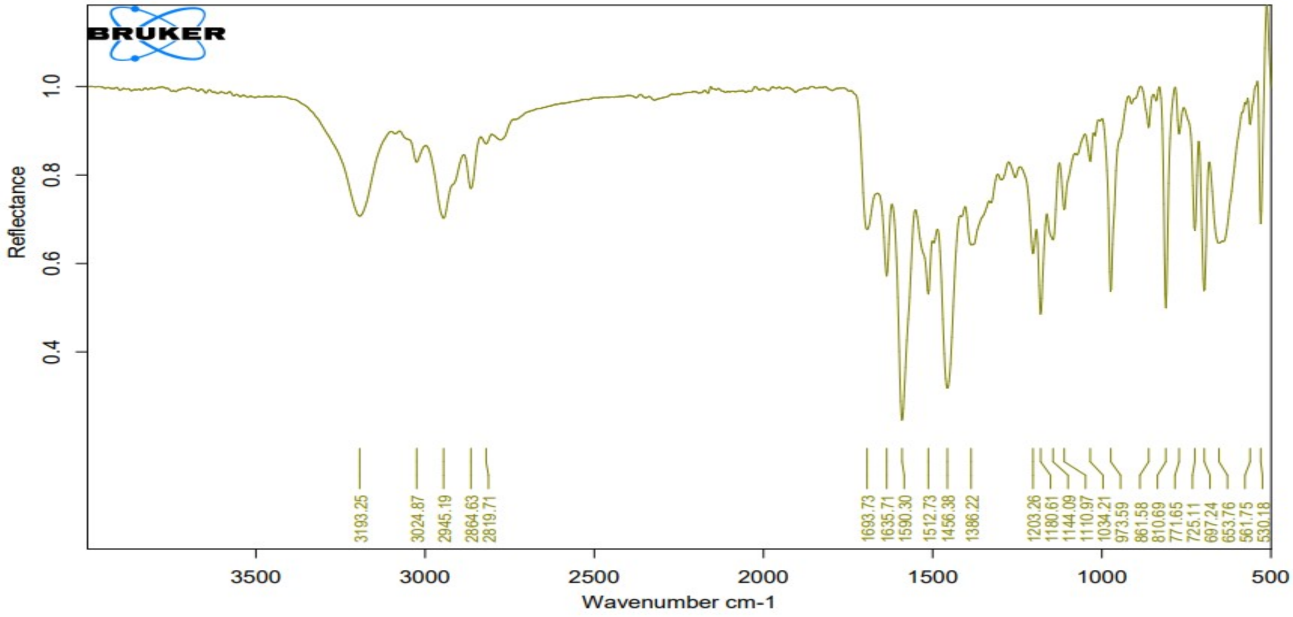

Figure S17: FT-IR spectrum of compound 6e

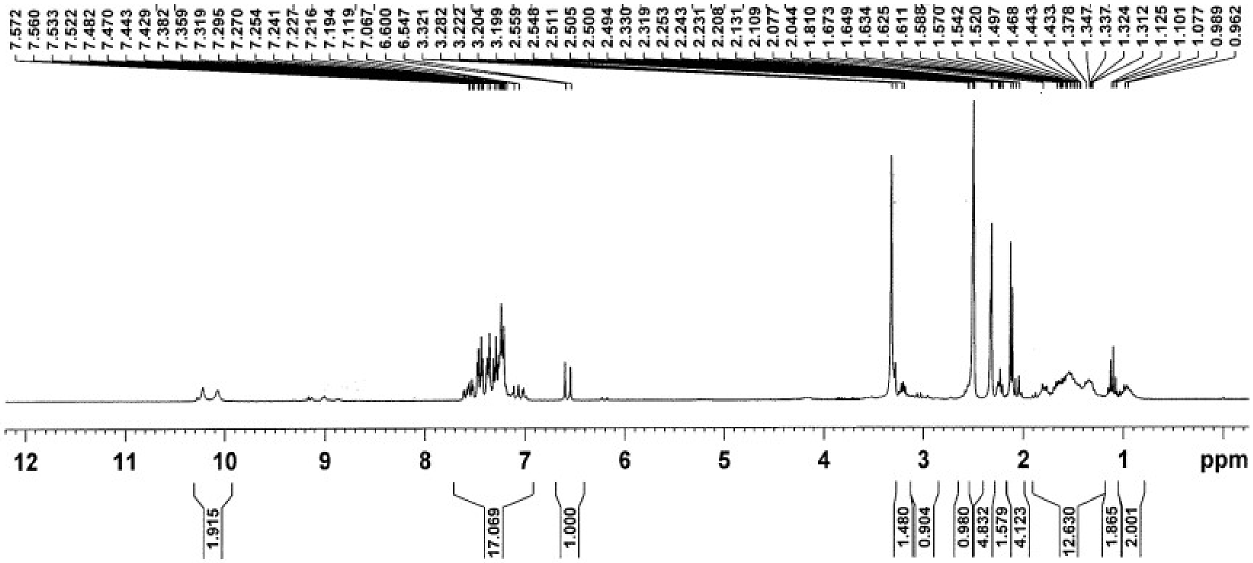

Figure S18: H[1] NMR spectrum of compound 6e

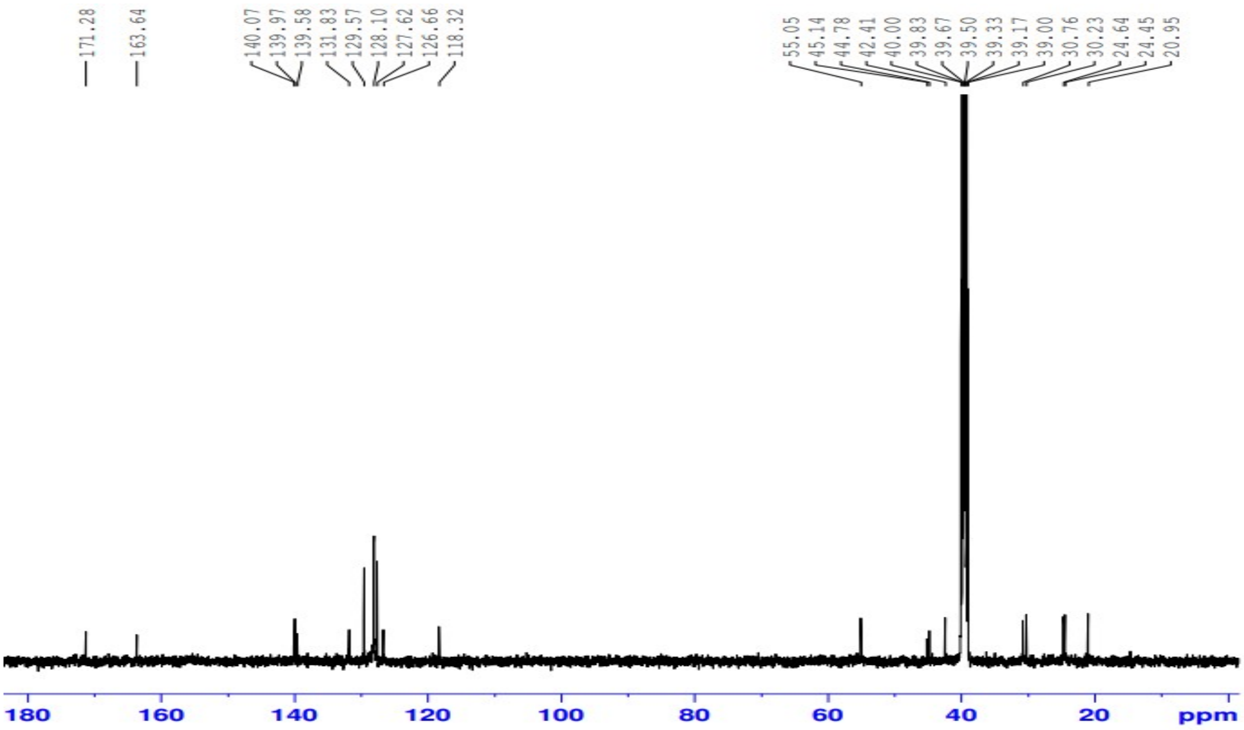

Figure S19: ^{13}C NMR spectrum (500 MHz, DMSO-d_6) of compound 6e

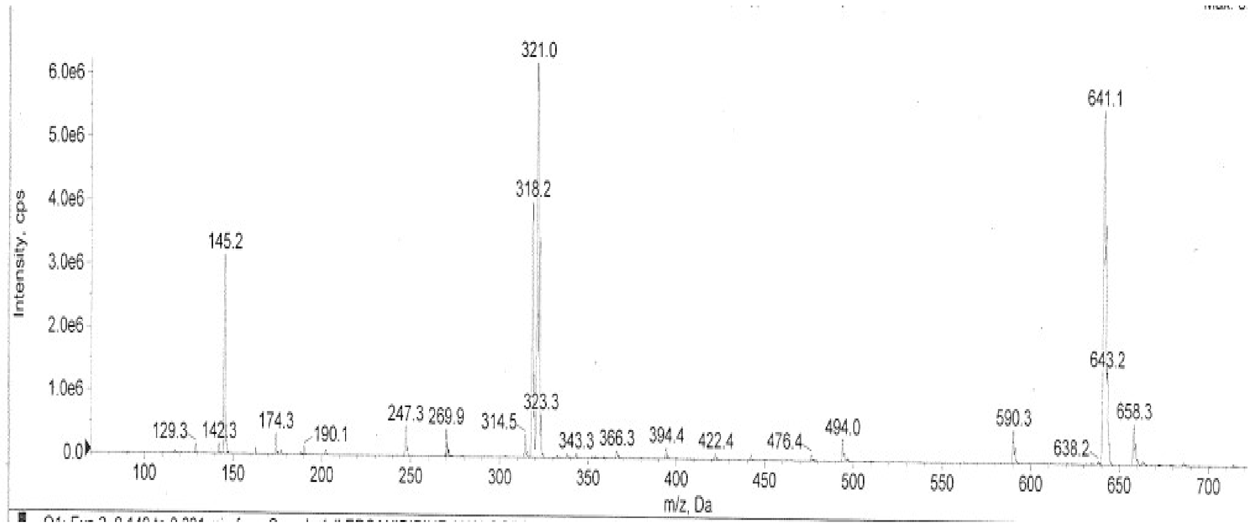

Figure S20: Mass spectrum of compound 6e

3-(4-Fluoro)-*N'*-(2-cyclopentyl-2-phenylacetyl)acrylohydrazide (6f):

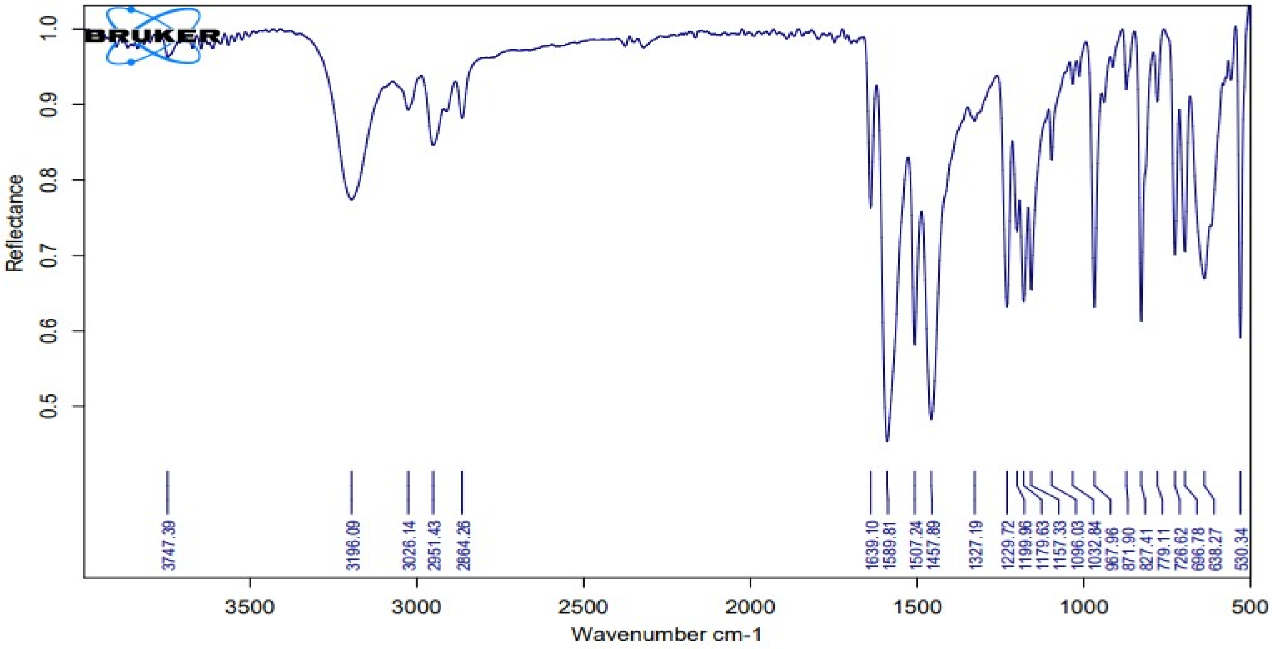

Figure S21: FT-IR spectrum of compound 6f

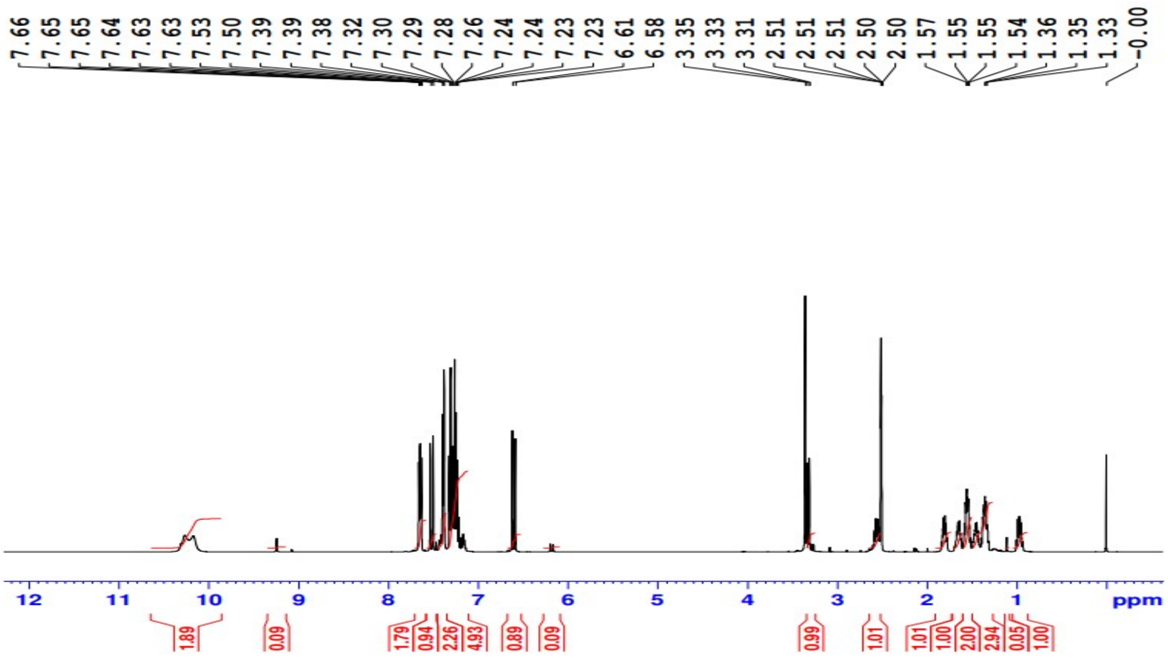

Figure S22: H[1] NMR spectrum of compound 6f

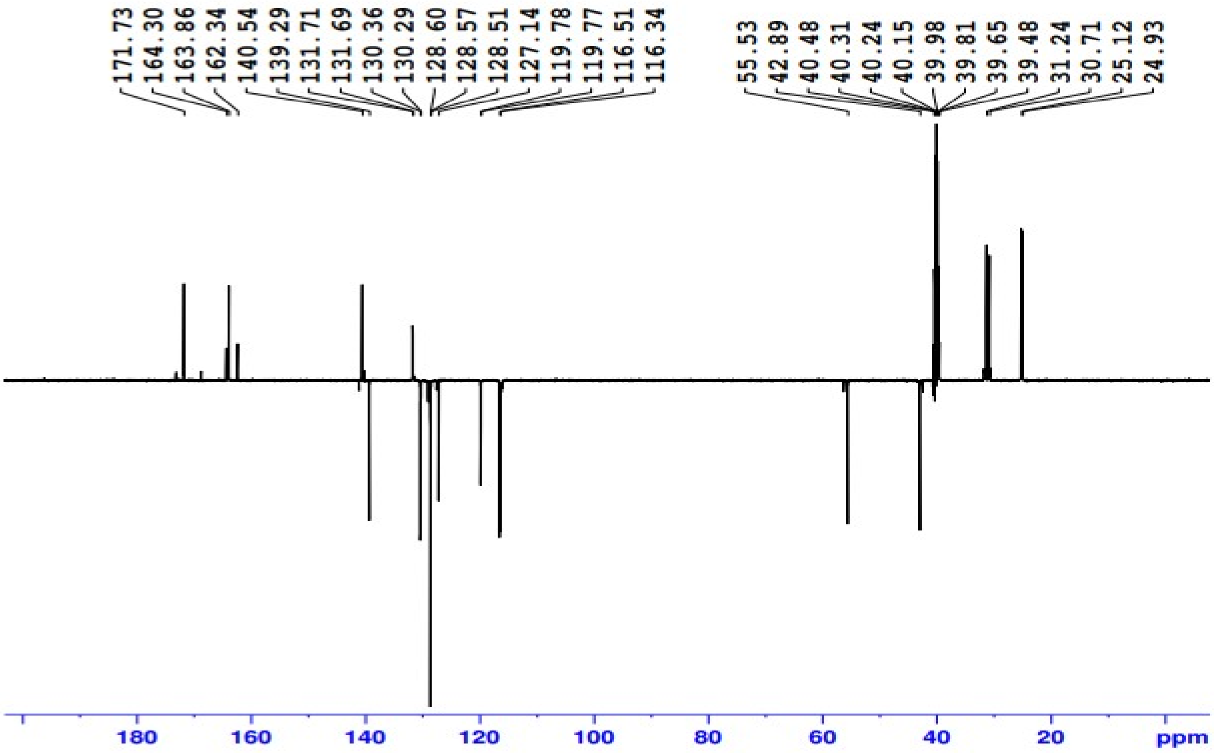

Figure S23: APT spectrum (500 MHz, DMSO-d_6) spectrum of compound 6f

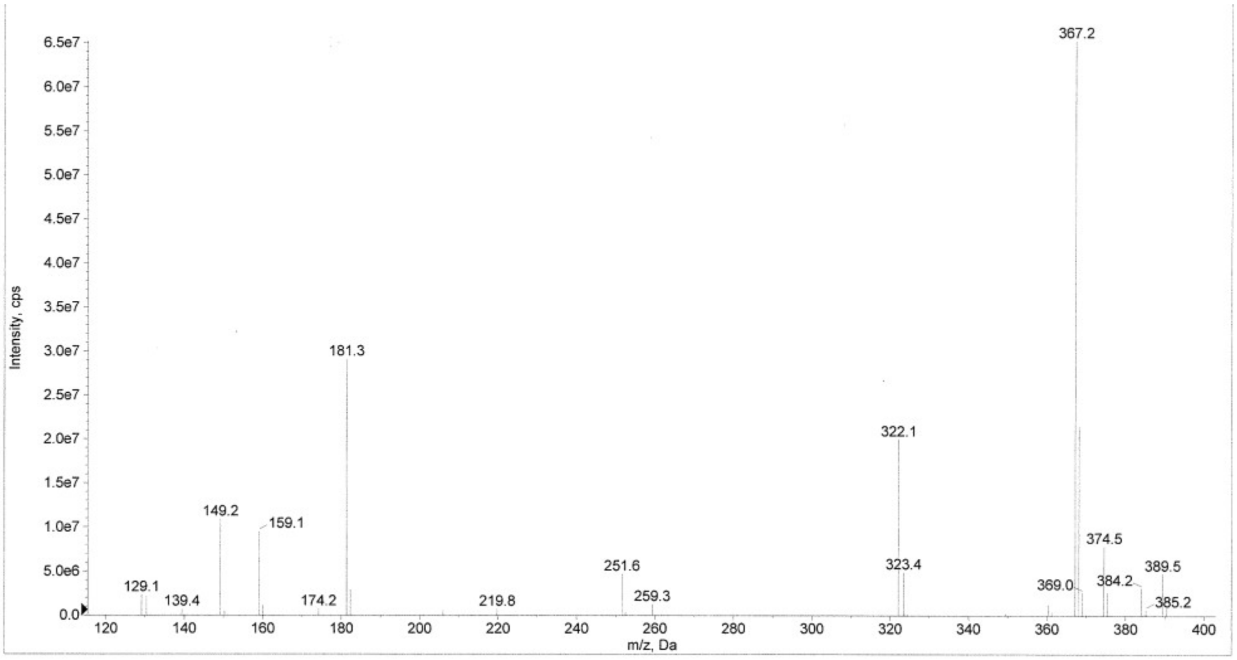

Figure S24: Mass spectrum of compound 6f

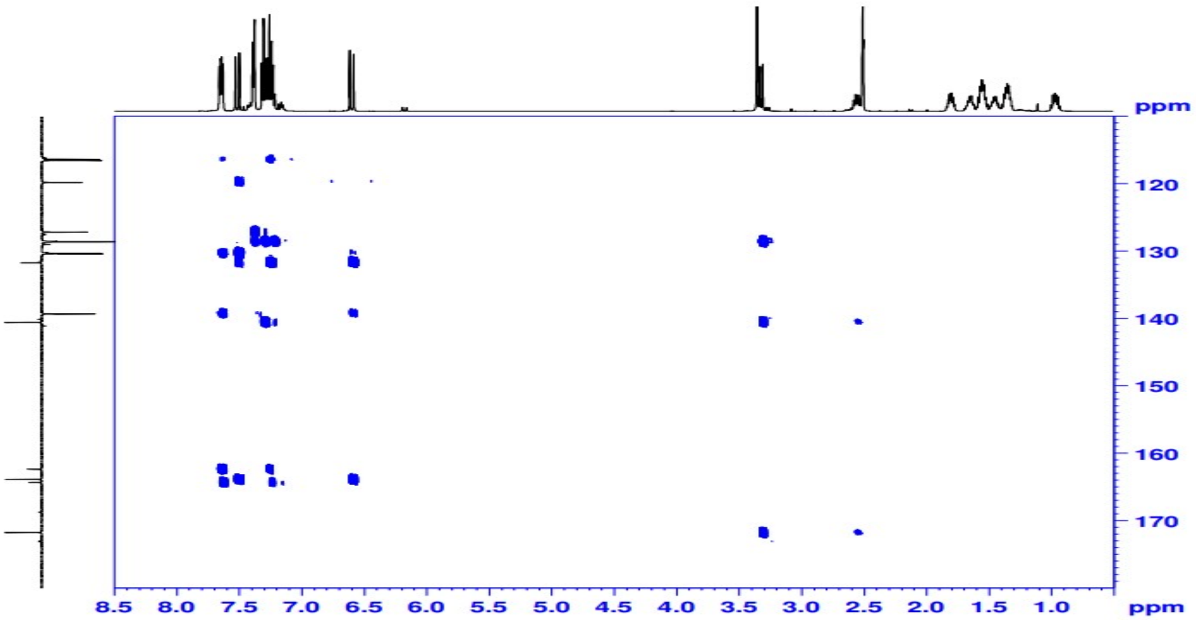

Figure S25: HMBC spectrum of compound 6f

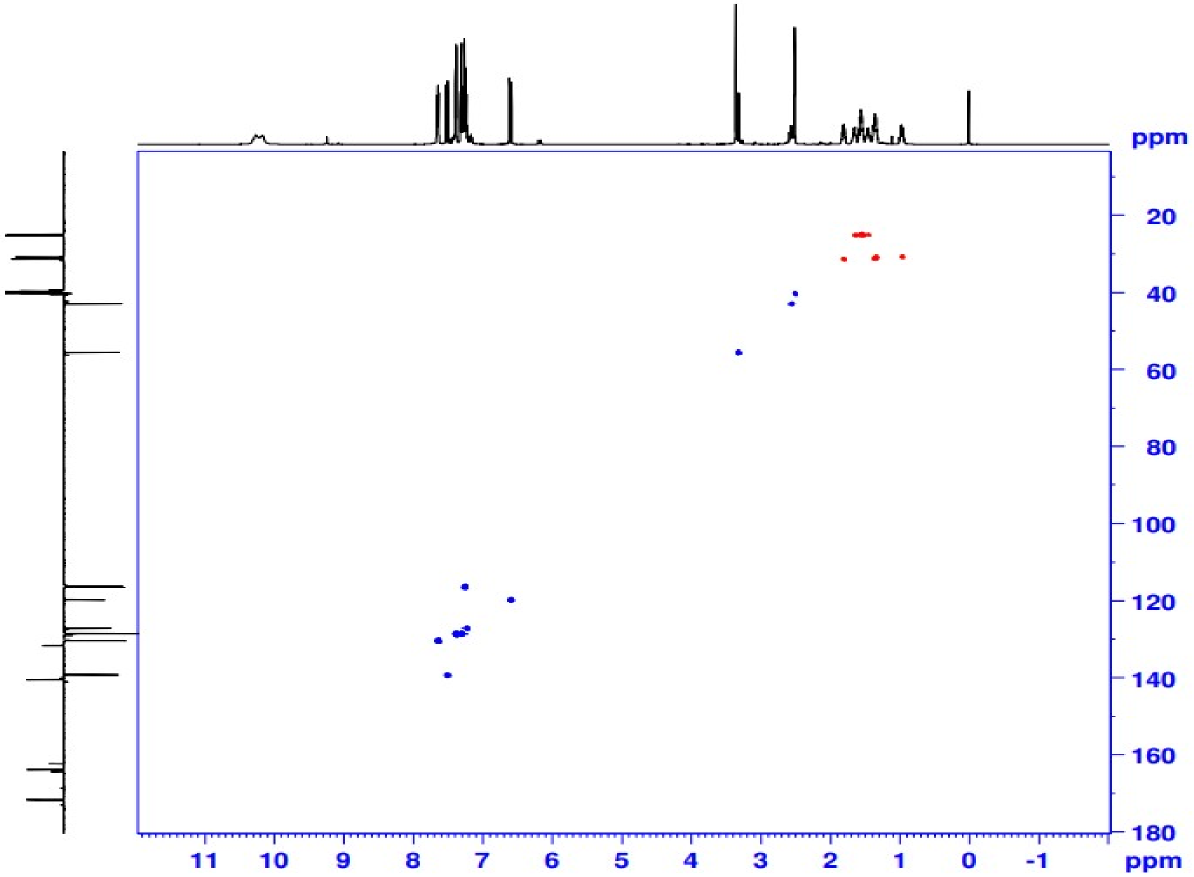

Figure S26: HSQC spectrum of compound 6f

Structural Elucidation of Derivative 6f:

We derived structural elucidation of derivative 6f with the help of ^{1}H, ^{13}C NMR, Attached Proton Test (APT), HMBC and HSQC spectral data (Table S1).

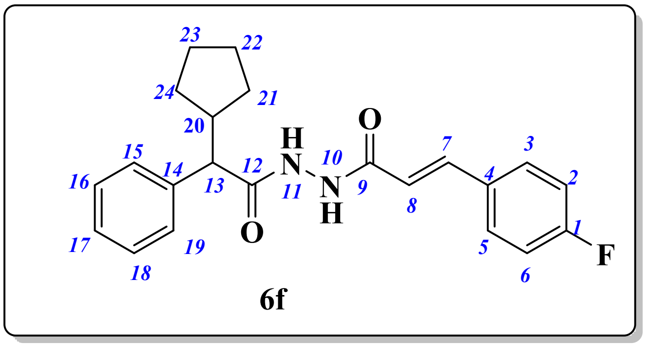

From the structure of derivative 6f it was clear that it has 5 quaternary carbon atoms (1, 4, 9, 12, 14), 4-CH_2 atoms (21, 22, 23, 24) and 9-CH atoms [(2,6), (3, 5), 7, 8, 13, 20, (15, 19), (16, 18), 17] in the structure. APT is indicated was cleared that the positive field peaks in the region of aromatic 131.71 (C-4), 140.54 (C-14), 163.86 (C-9), 179.73 (C-12) and 162.34-164.30 (C-1) indicates the presence of Quaternary carbon atoms. The higher ^{13}C values of 179.73 and 163.86 indicates the presence of two amide keto (-NH-C=0) groups. The coupling constant ($J_{C\text{-}C}$) of C-1 carbon of value 255 Hz indicated the presence of C-F coupling at C-1 carbon atom of the compound 6f. The positive field peaks in the region of 24.93 (C-21), 25.12 (C-24), 30.71 (C-22), 31.24 (C-23) indicates the presence of 4-CH_2 atoms.

The negative field peaks of 116.34-116.51 (C-2,6), 130.29-130.36 (C-3,5), 119.78 (C-7), 139.29 (C-8), 55.53 (C-13), 42.89 (C-20), 128.6 (C-15,19), 128.57 (C-16,18), 127.14 (C-17), indicates the existence of 9-CH carbon atoms. Whereas the coupling constant value of 21.25 Hz at C-2,6 carbon atoms indicates the presence of C-F coupling. The J_{HH} coupling constant value at C-7 and C-8 atoms of value 15 Hz conforms the presentation of trans isomer. There is no other protons in the olefine region and no proton (1-en-1-ol) in down field of the ^{1}H NMR indicates that there is absence of tautomer's in the compound the same was confirmed by the ^{13}C NMR spectrum.

Table S1: ^{1}H, ^{13}C and 2D NMR data interpretation of **6f.**

**Assignment**	**Type of atom**	**^{1}H, δ (ppm) (Multiplicity, J_{HH} in Hz, number of protons)**	**^{13}C, δ (ppm)**	**^{1}H-^{13}C HMBC**
1	**C**	**-**	**162.34, 164.30**	130.29 (C-3), 130.36 (C-5), 116.34 (C-2), 116.51 (C-6)
2, 6	**CH**	7.25 (m, 2H)	**116.34, 116.51**	-
3, 5	**CH**	7.68 (dd, *J* = 5 Hz, 2H)	**130.29, 130.36**	-
4	**C**	-	**131.71**	130.29 (C-3), 130.36 (C-5), 139.29 (C-8)
7	**CH**	6.61-6.55 (d, *J* = 15.9 Hz, 1H)	**119.78**	139.29 (C-8), 163.86 (C-9), 131.71 (C-4)
8	**CH**	7.53-7.50 (d, *J* = 15 Hz, 1H)	**139.29**	119.78 (C-7), 131.71 (C-4), 163.86 (C-9)
9	**C=O**	-	**163.86**	119.78 (C-7), 139.29 (C-8)
10	**NH**	10.3 (b, 1H)	**-**	-
11	**NH**	10.3 (b, 1H)	**-**	-
12	**C=O**	-	**171.73**	42.89 (C-20), 55.53 (C-13)
13	**CH**	3.31-3.33 (d, *J* =	**55.53**	140.54 (C-14), 179.73 (C-12), 42.89 (C-20),

		10 Hz, 1H)		24.93 (C-21), 25.12 (C-24)
14	**C**	-	**140.54**	128.57 (C-16, 18), 55.53 (C-13), 42.89 (C-20)
15, 19	**CH**	7.39 (dd, *J* = 5 Hz, 2H)	**128.6**	128.57 (C-16, 18), 127.17 (C-17), 55.53 (C-13)
16, 18	**CH**	7.3 (m, 2H)	**128.57**	127.17 (C-17), 128.6 (C-15, 19)
17	**CH**	7.26 (m, 2H)	**127.14**	128.57 (C-16, 18), 128.6 (C-15, 19)
20	**CH**	2.56 (m, 1H)	**42.89**	55.53 (C-13), 30.71 (C-22), 31.74 (C-23)
21, 24	**CH_2**	1.45 (m), 1.55 (m), 1.65 (m) (4H)	**24.93, 25.12**	55.53 (C-13), 30.71 (C-22), 31.74 (C-23), 42.89 (C-20)
22, 23	**CH_2**	0.98 (m), 1.35 (m), 1.8 (m) (4H)	**30.71, 31.74**	55.53 (C-13), 42.89 (C-20), 24.93 (C-21), 25.12 (C-24)

d = doublet, m = multiplet, dd = doublet of doublet.

3-(3-Nitrophenyl)-*N’*-(2-cyclopentyl-2-phenylacetyl)acrylohydrazide (6g):

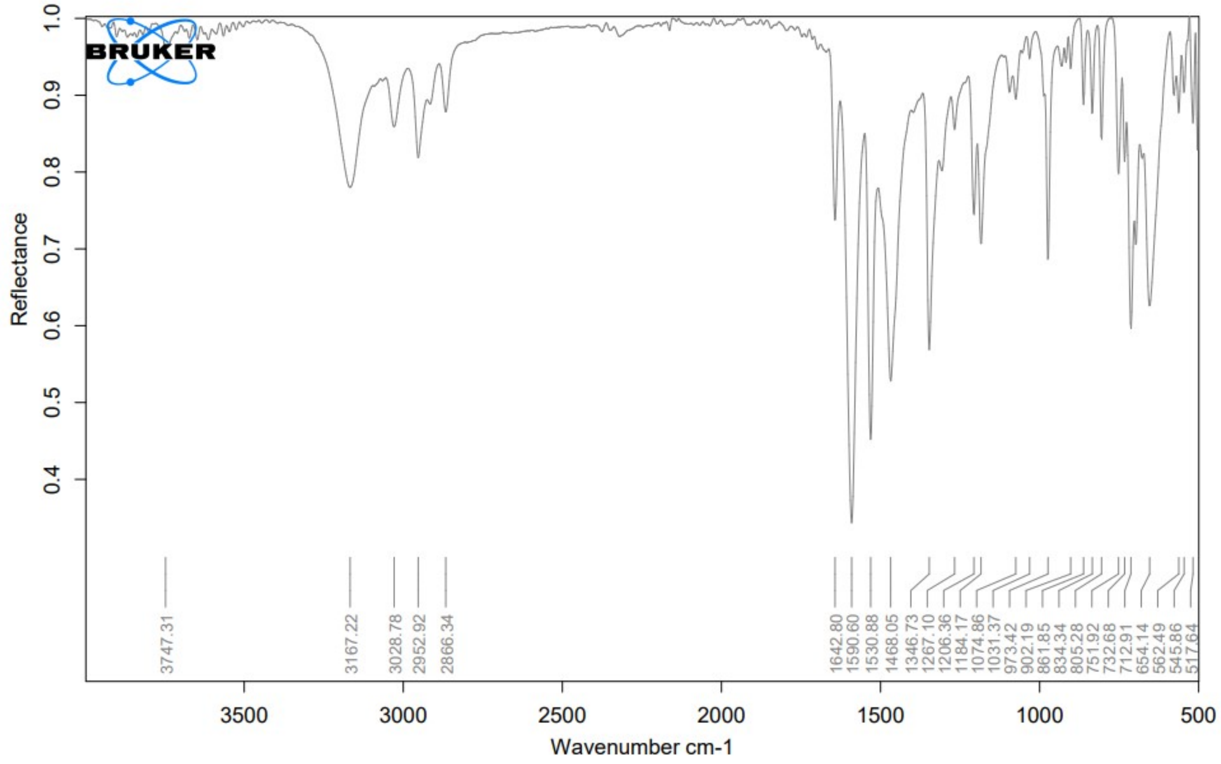

Figure S27: FT-IR spectrum of compound 6g

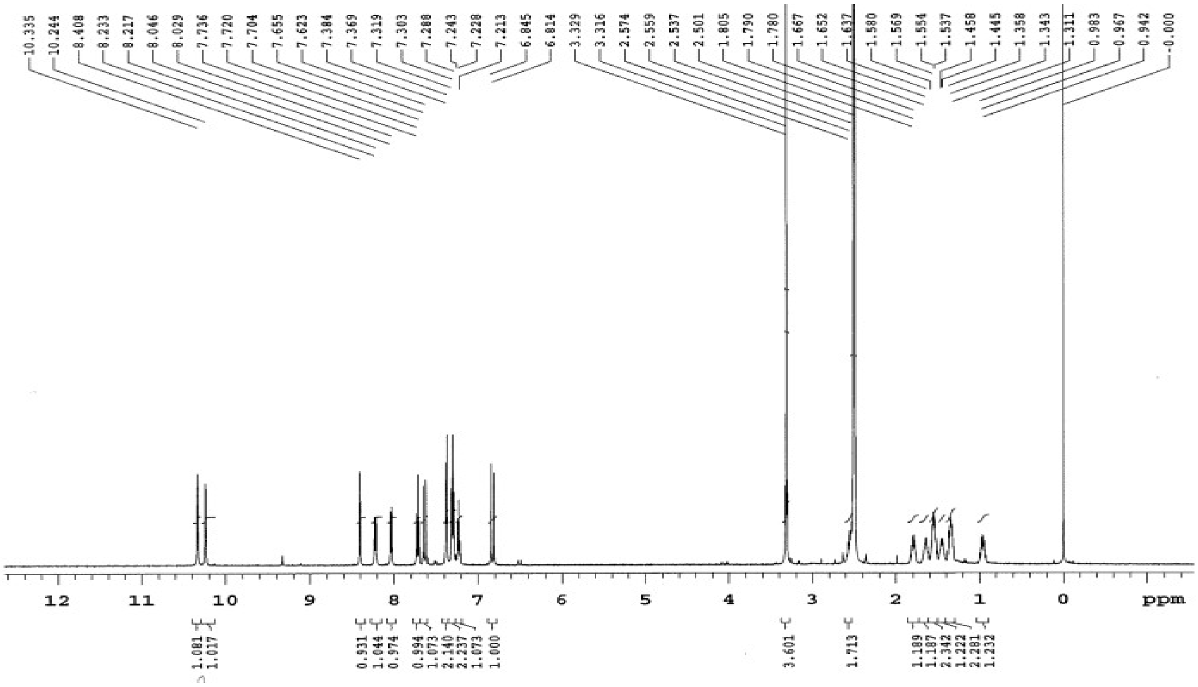

Figure S28: H[1] NMR spectrum of compound 6g

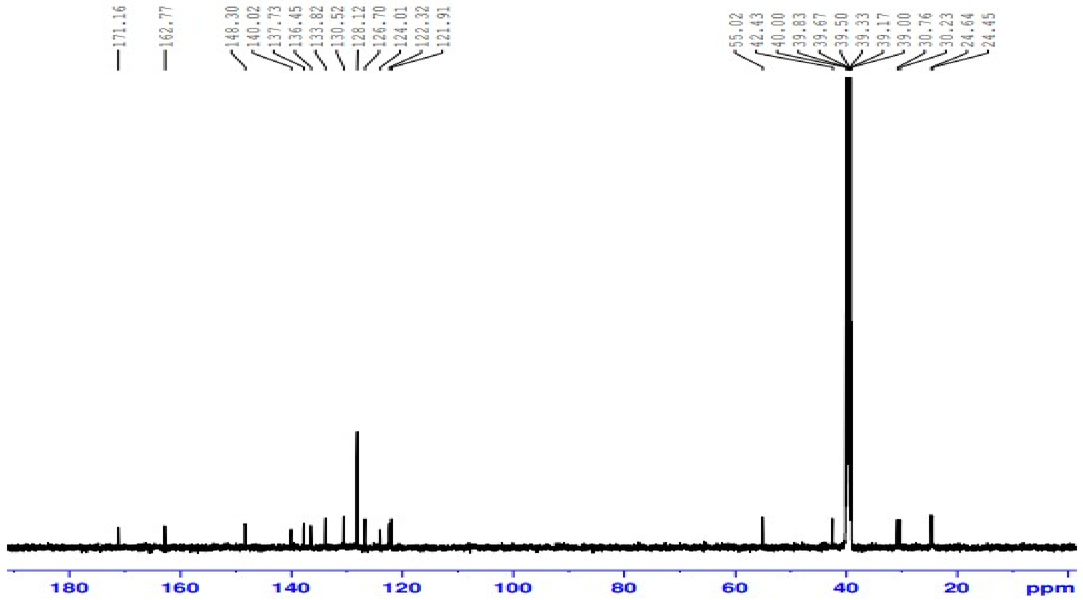

Figure S29: ^{13}C NMR spectrum (500 MHz, DMSO-d_6) spectrum of compound 6g

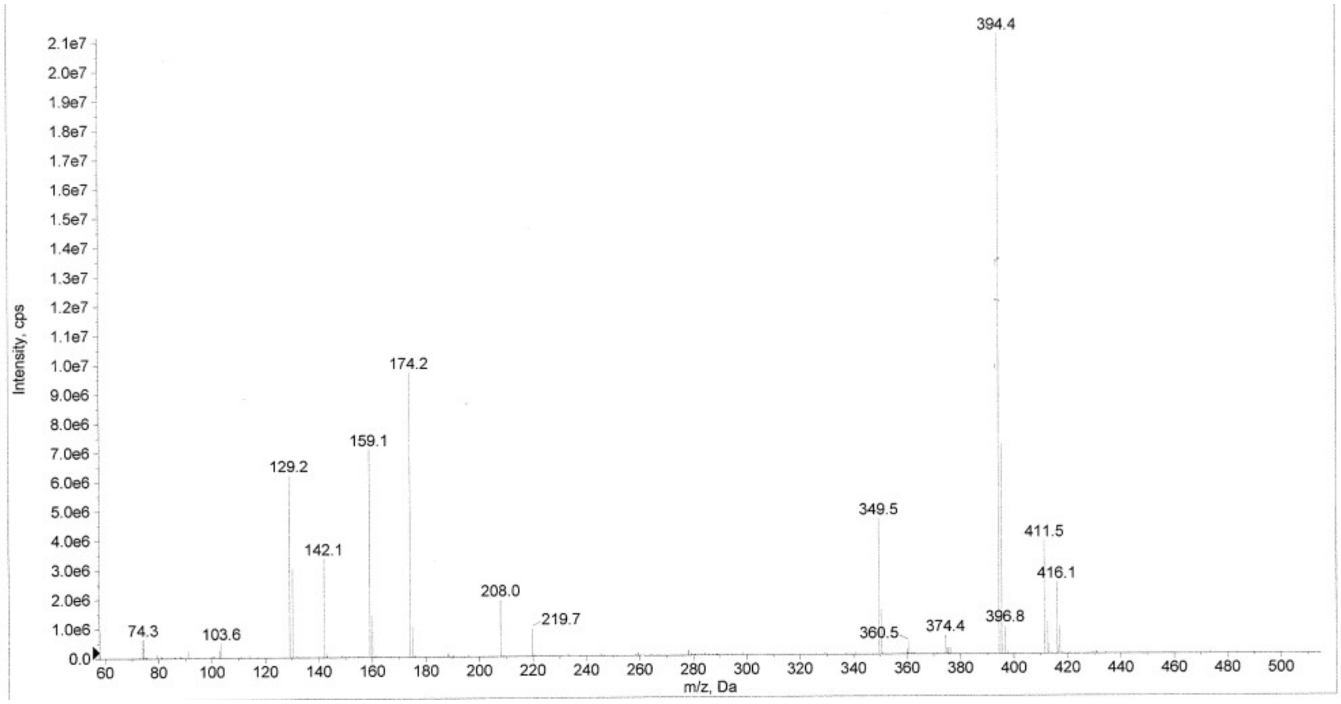

Figure S30: Mass spectrum of compound 6g

***N'*-(2-cyclopentyl-2-(2,4,5-trifluorophenyl)acetyl)cinnamohydrazide (7a):**

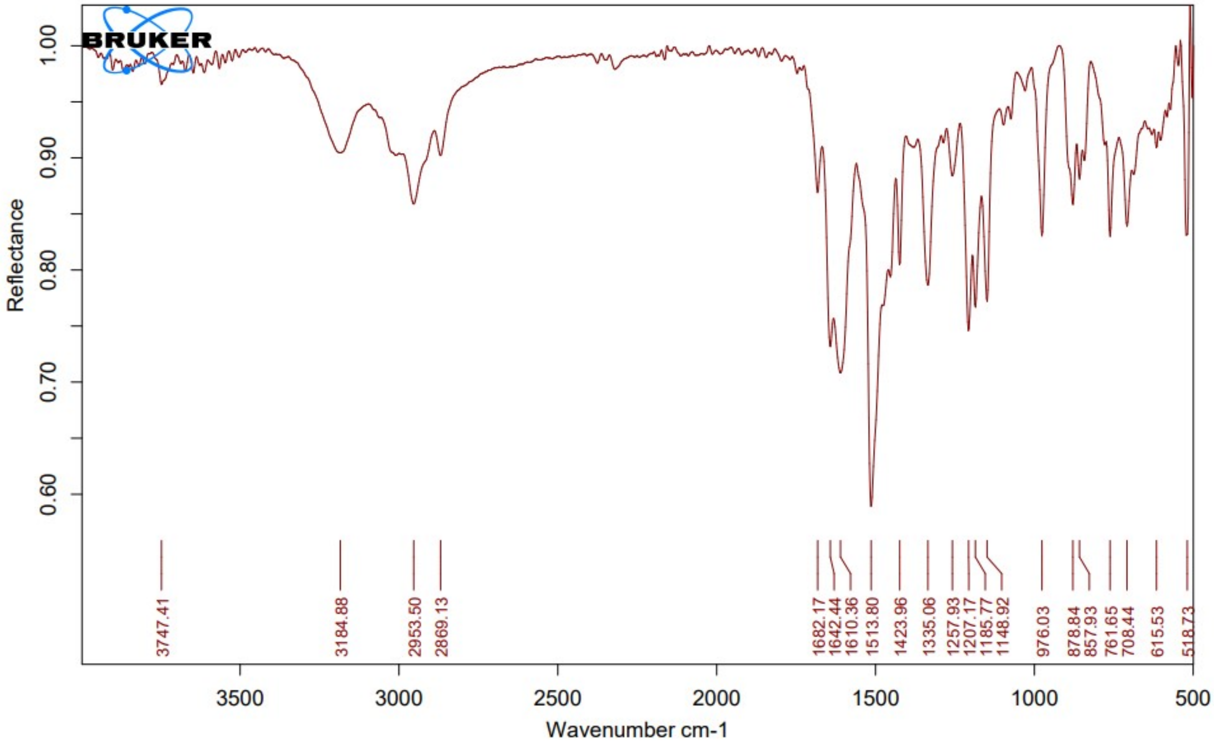

Figure S31: FT-IR spectrum of compound 7a

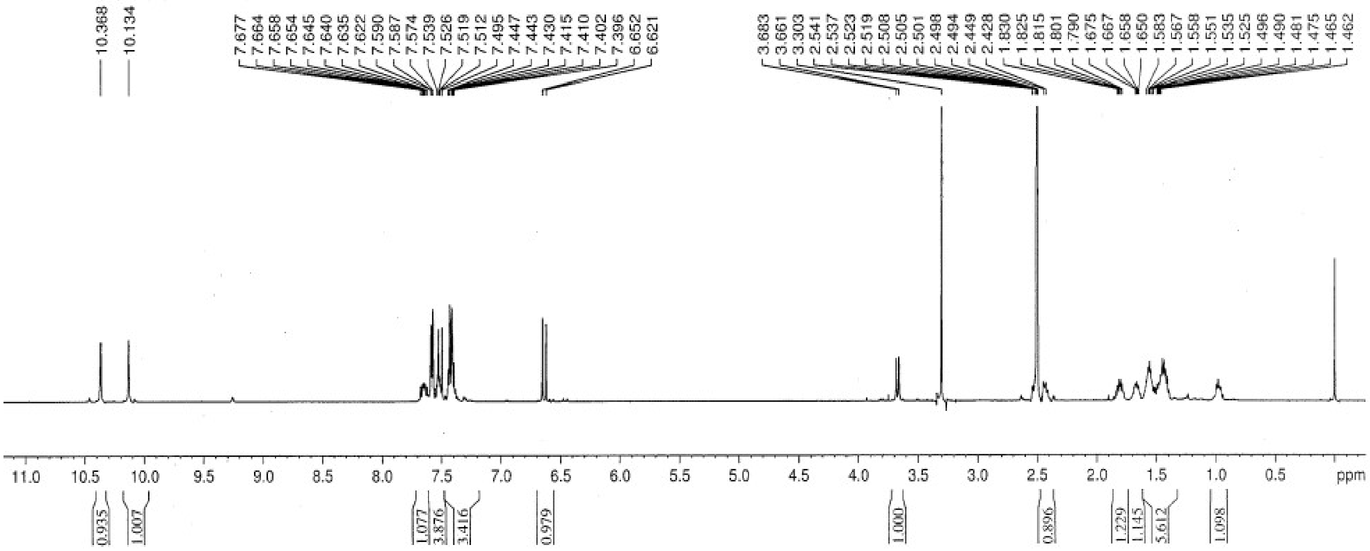

Figure S32: H[1] NMR spectrum of compound 7a

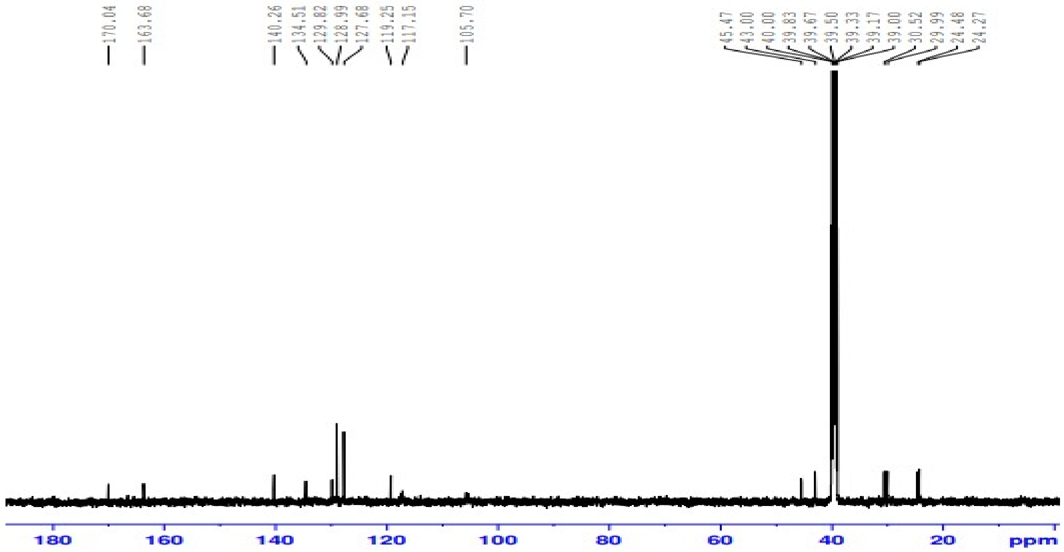

Figure S33: ^{13}C NMR spectrum (500 MHz, DMSO-d_6) spectrum of compound 7a

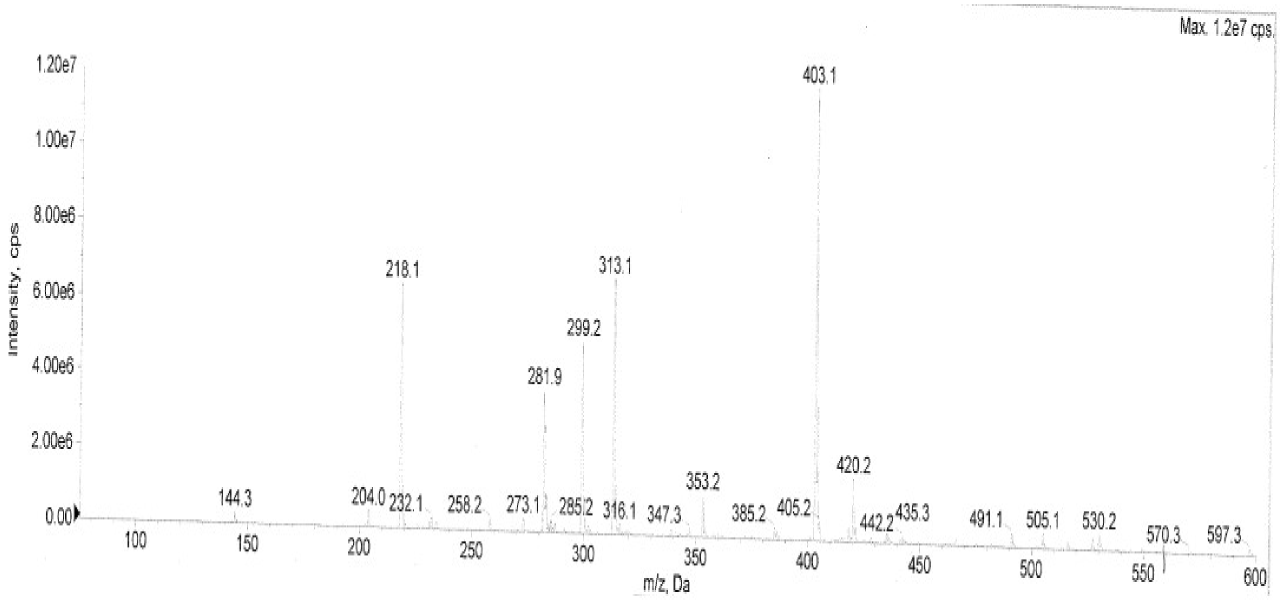

Figure S34: Mass spectrum of compound 7a

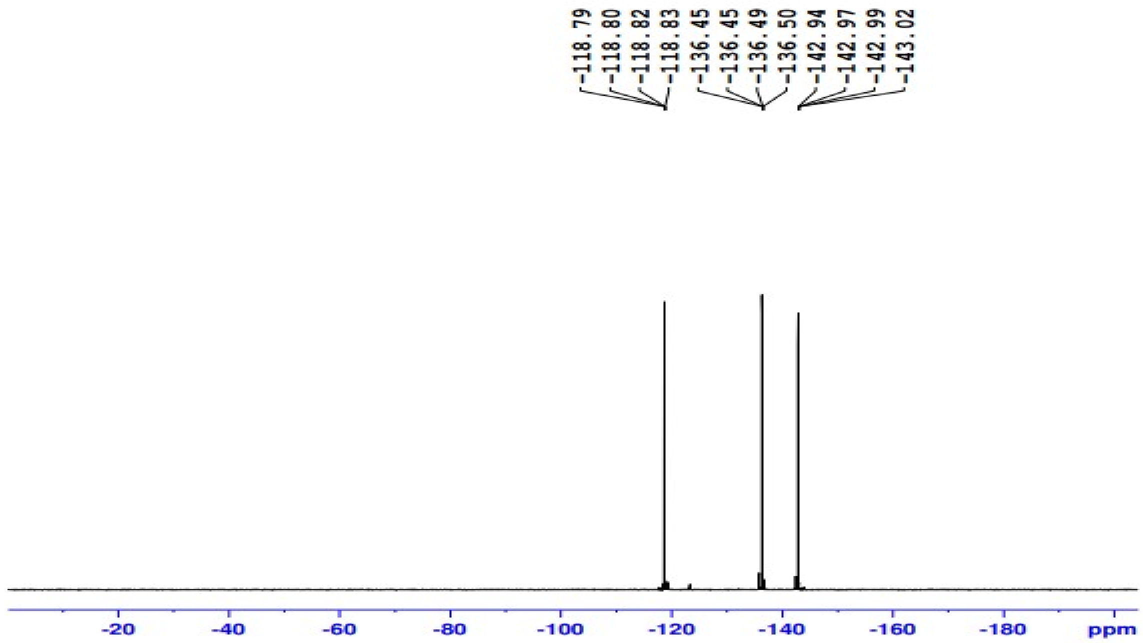

Figure S35: ^{19}F spectrum of compound 7a

***N'*-(2-cyclopentyl-2-(2,4,5-trifluorophenyl)acetyl)-3-(p-tolyl)acrylohydrazide (7b):**

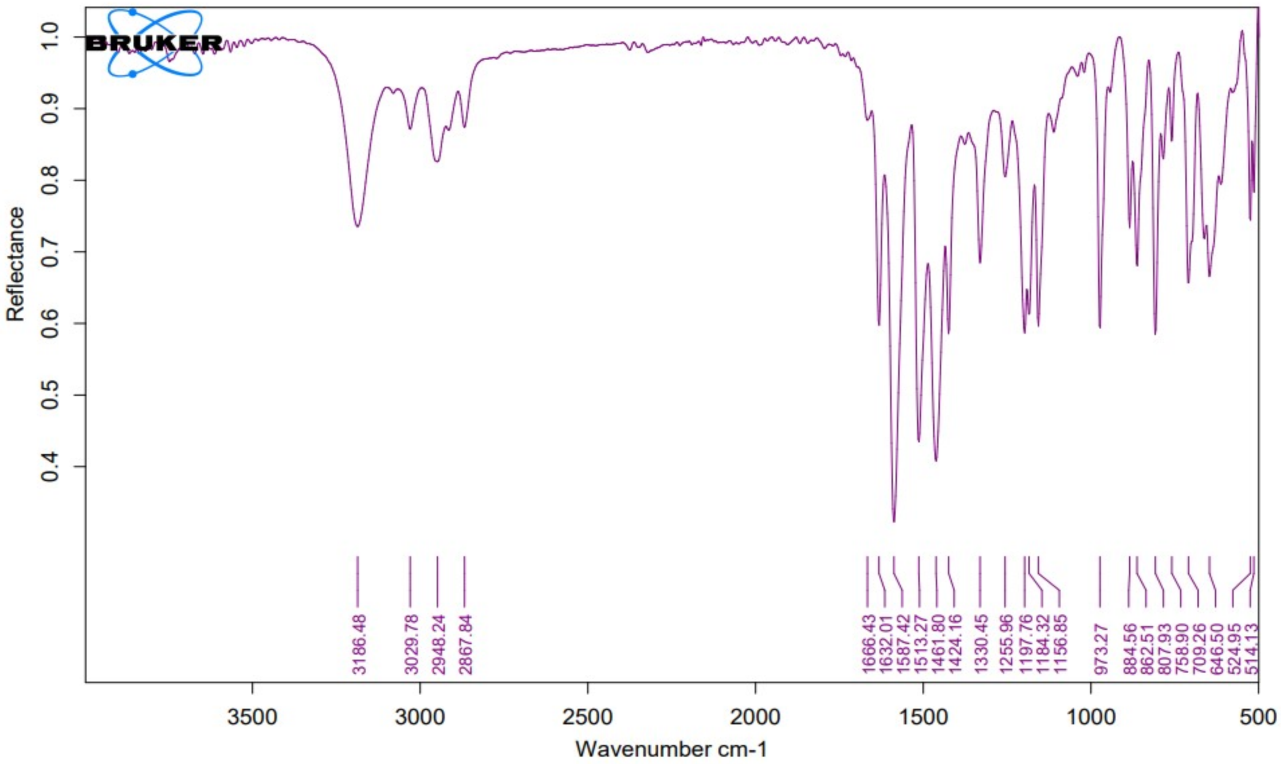

Figure S36: FT-IR spectrum of compound 7b

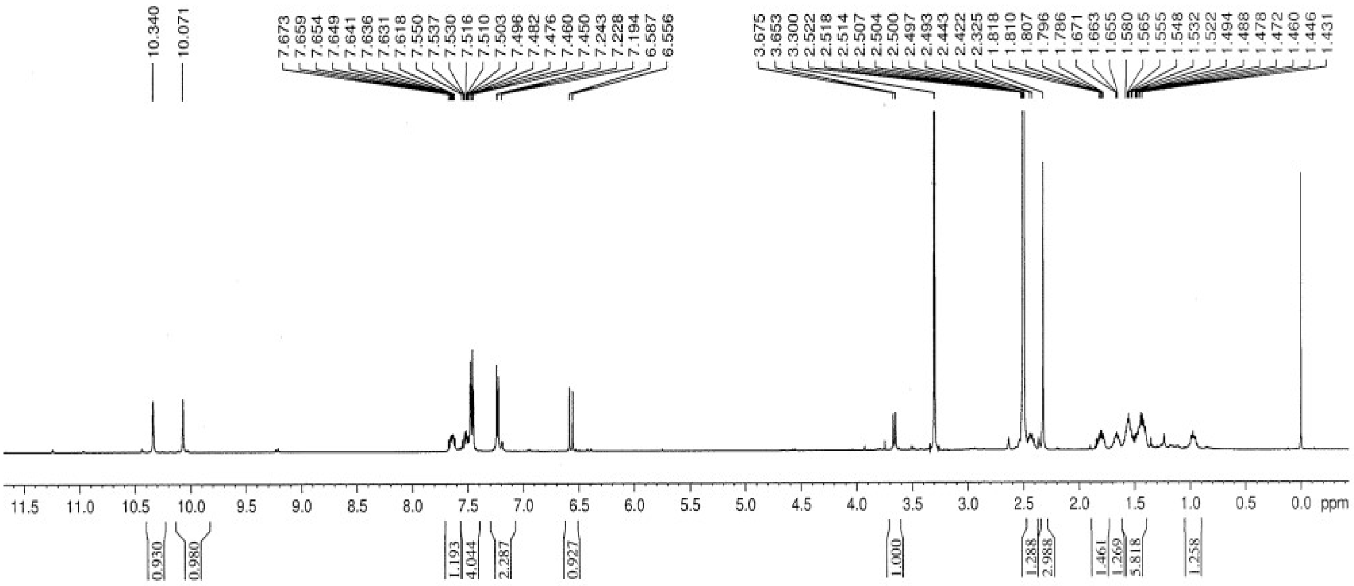

Figure S37: H[1] NMR spectrum of compound 7b

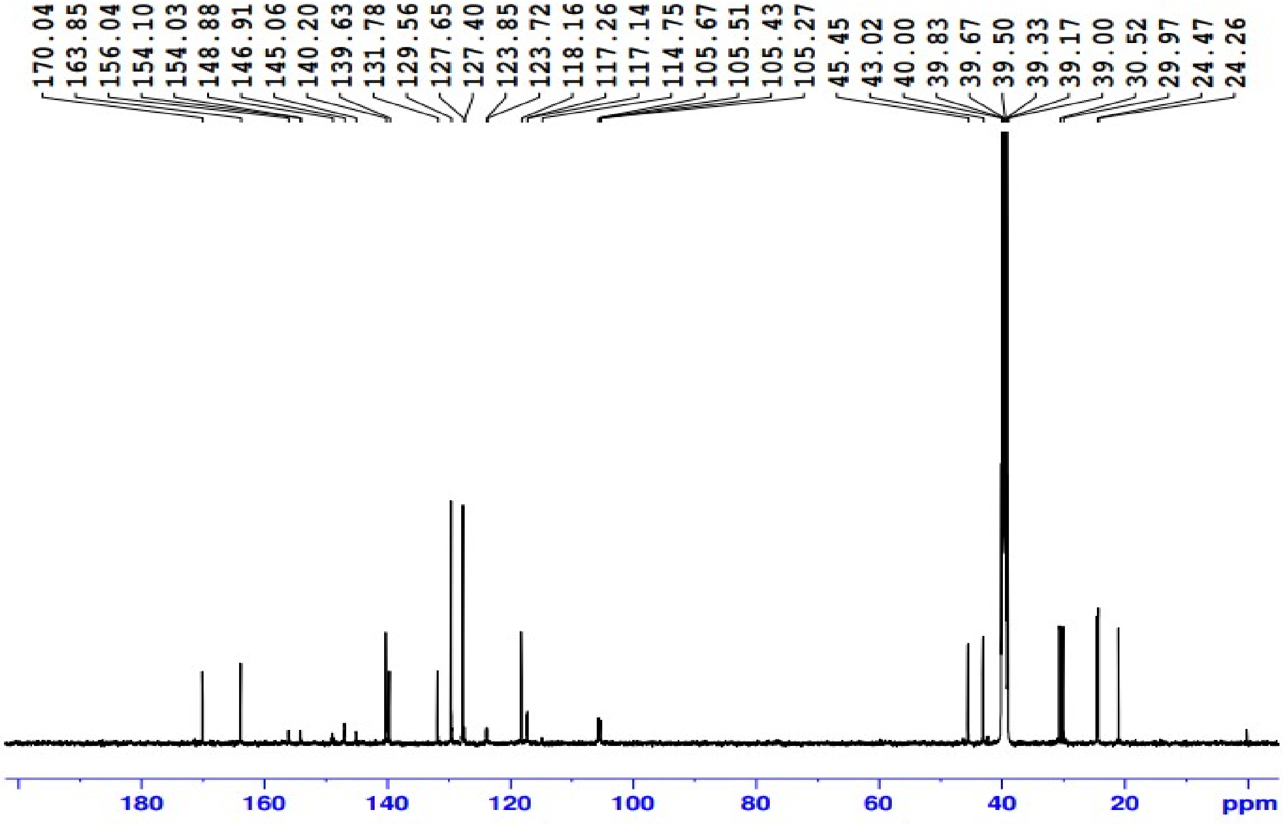

Figure S38: ^{13}C NMR spectrum (500 MHz, DMSO-d_6) spectrum of compound 7b

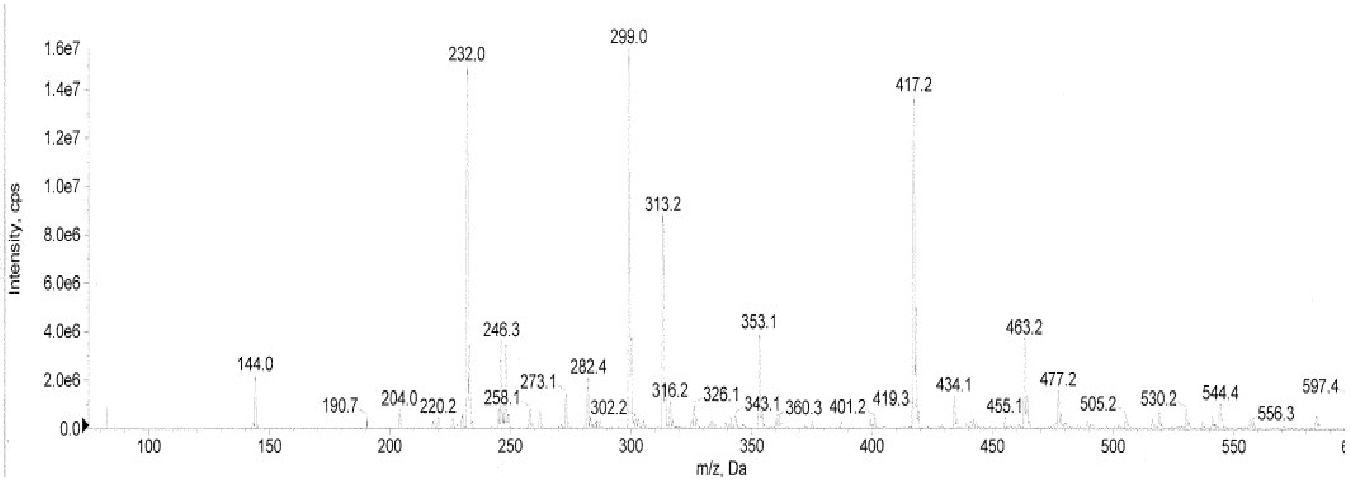

Figure S39: Mass spectrum of compound 7b

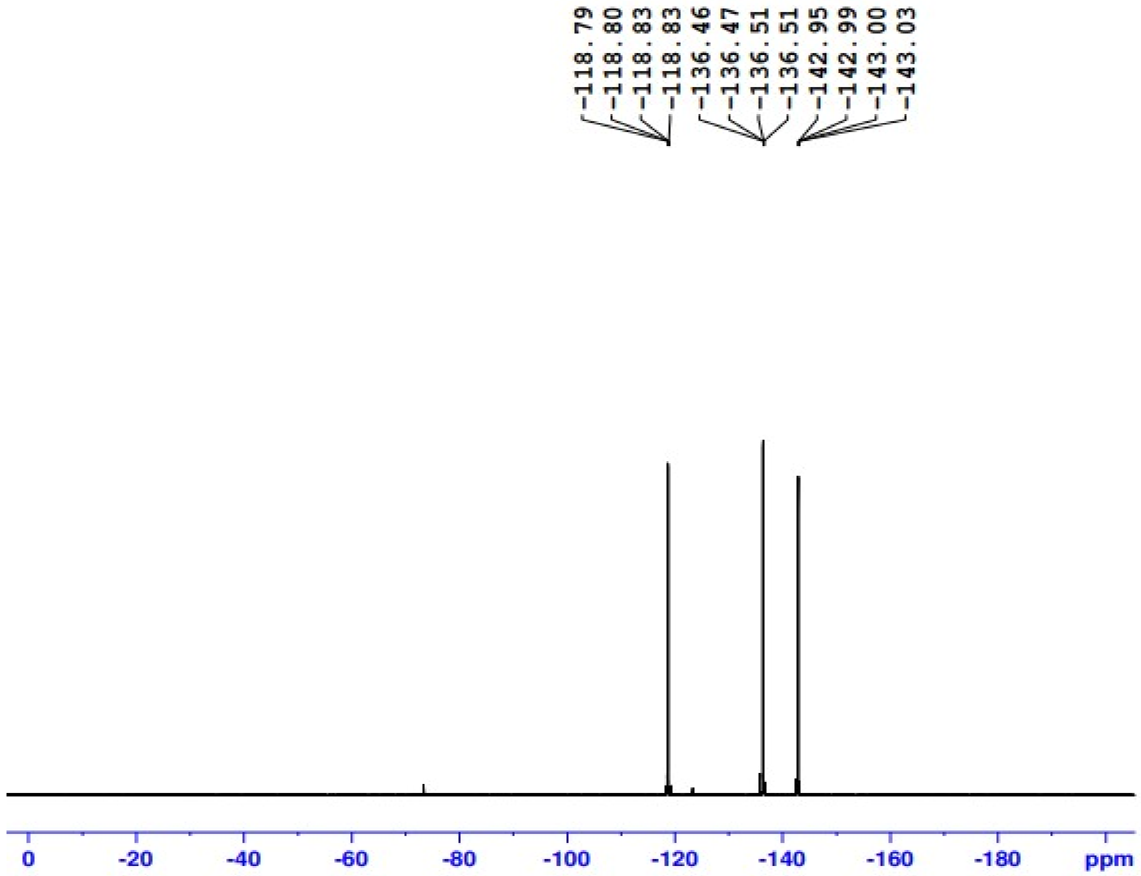

Figure S40: ^{19}F spectrum of compound 7b

3-(4-bromophenyl)-*N'*-(2-cyclopentyl-2-(2,4,5-trifluorophenyl)acetyl)acrylohydrazide (7c):

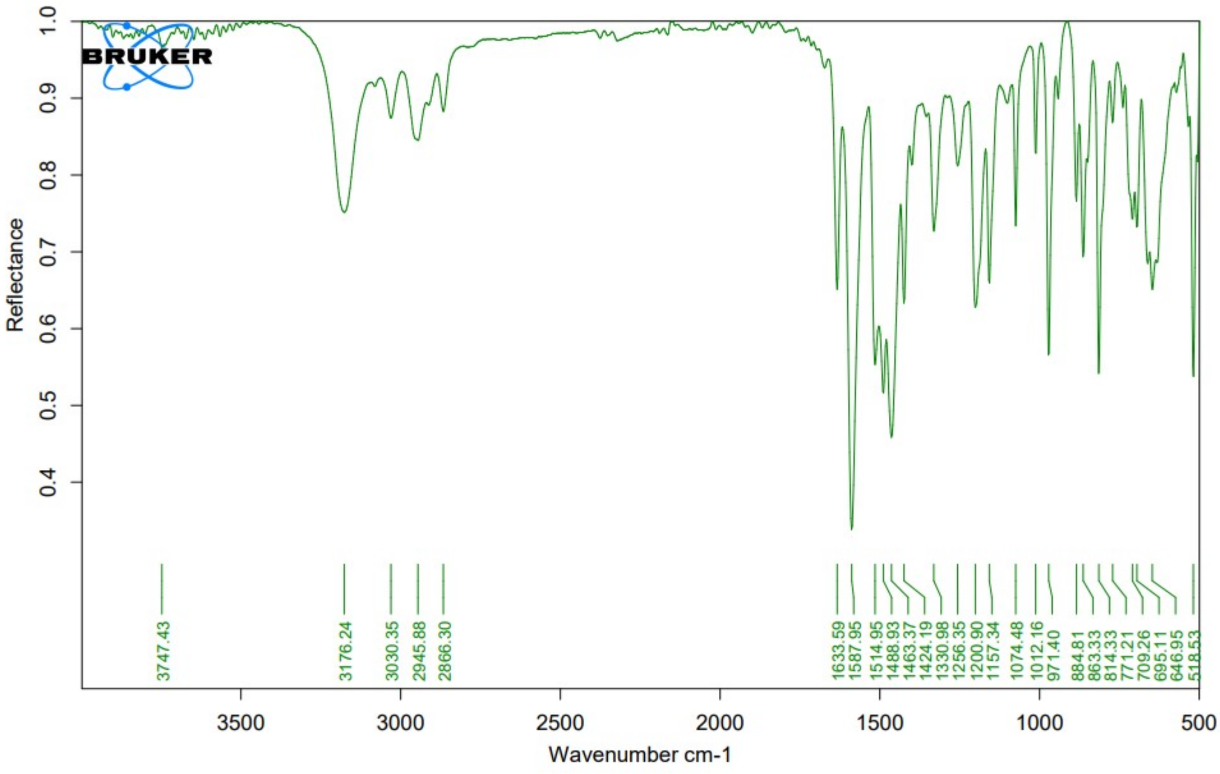

Figure S41: FT-IR spectrum of compound 7c

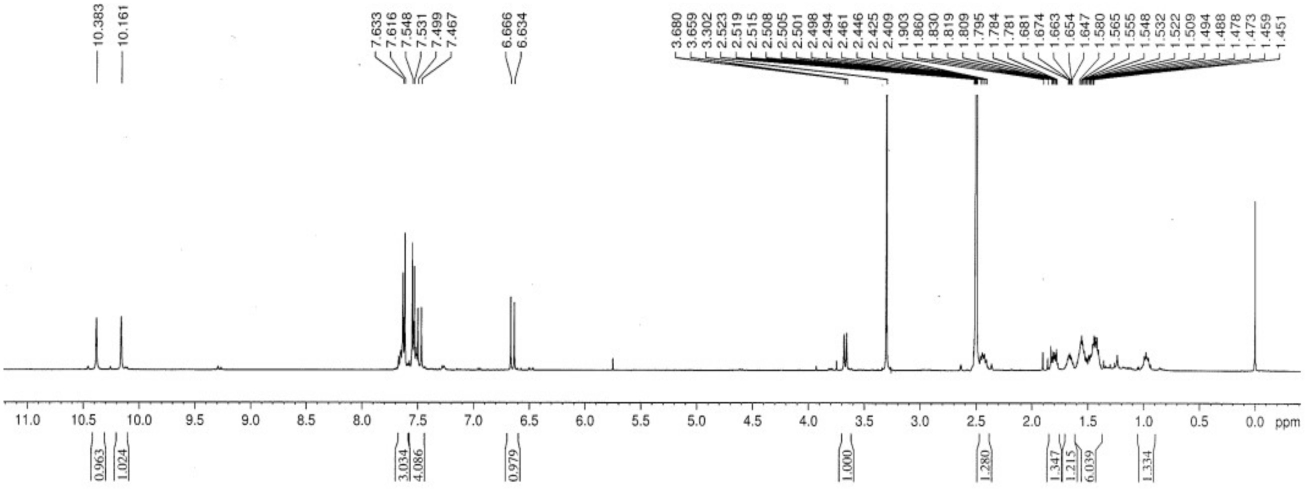

Figure S42: H[1] NMR spectrum of compound 7c

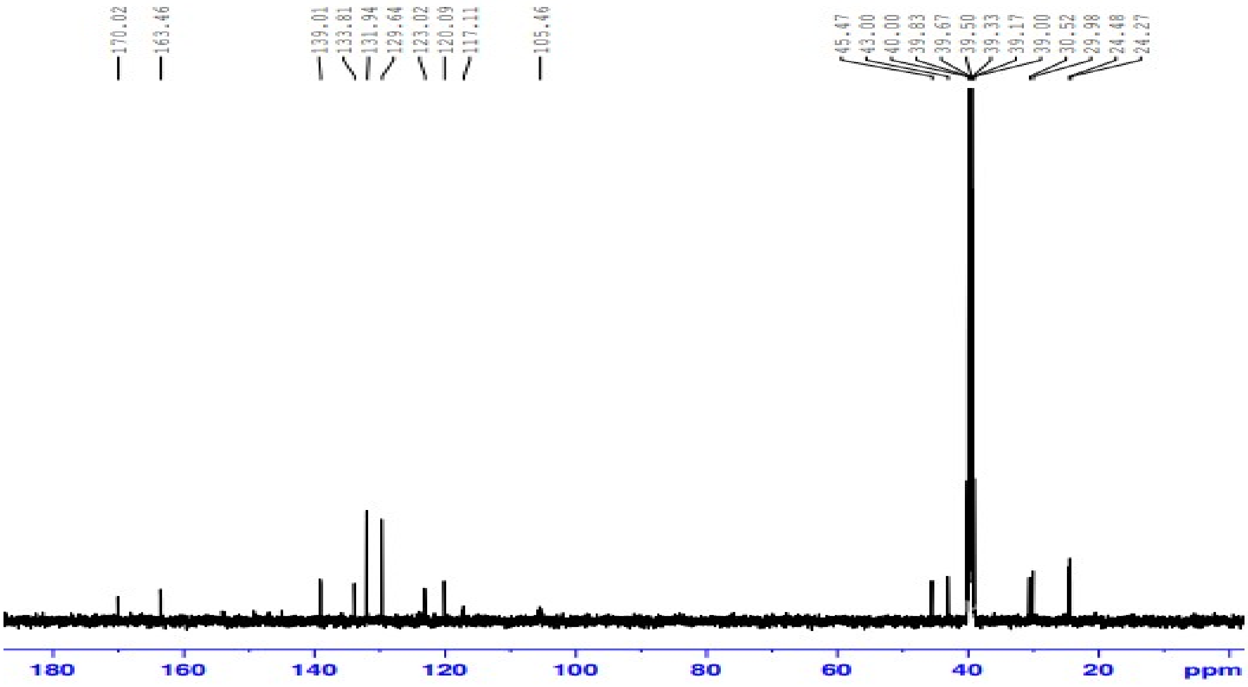

Figure S43: ^{13}C NMR spectrum (500 MHz, DMSO-d_6) spectrum of compound 7c

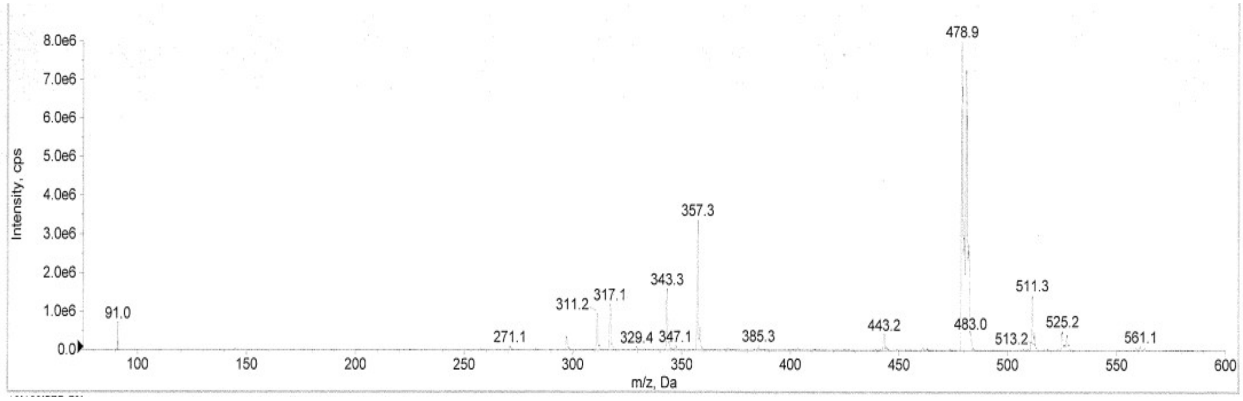

Figure S44: Mass spectrum of compound 7c

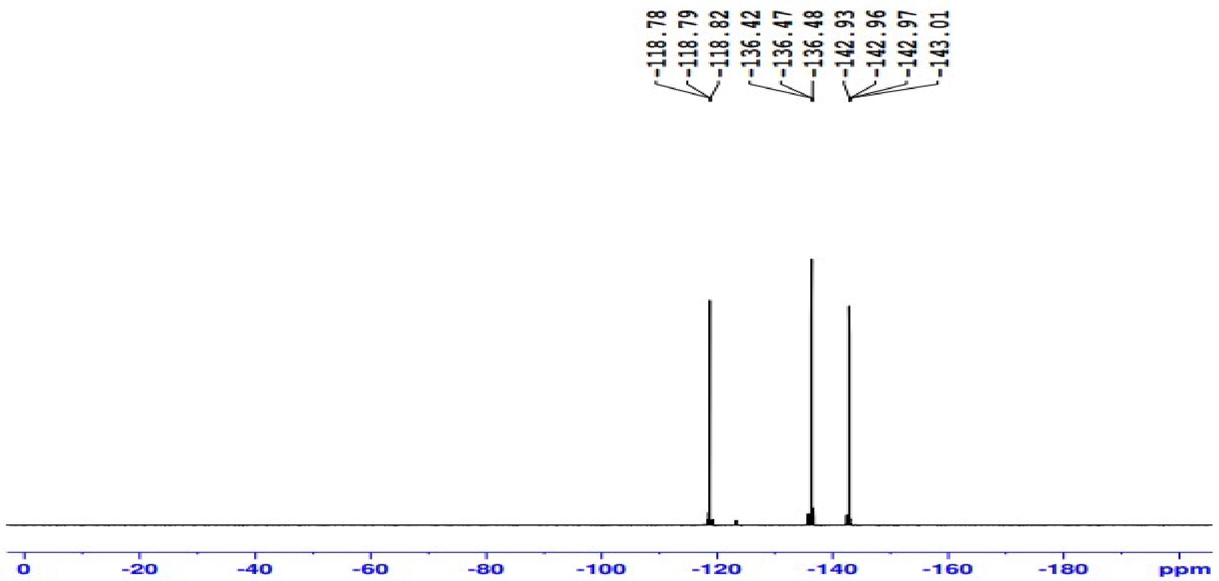

Figure S45: ^{19}F spectrum of compound 7c

3-(3-Chlorophenyl)-*N'*-(2-cyclopentyl-2-(2,4,5-trifluorophenyl)acetyl)acrylohydrazide (7d):

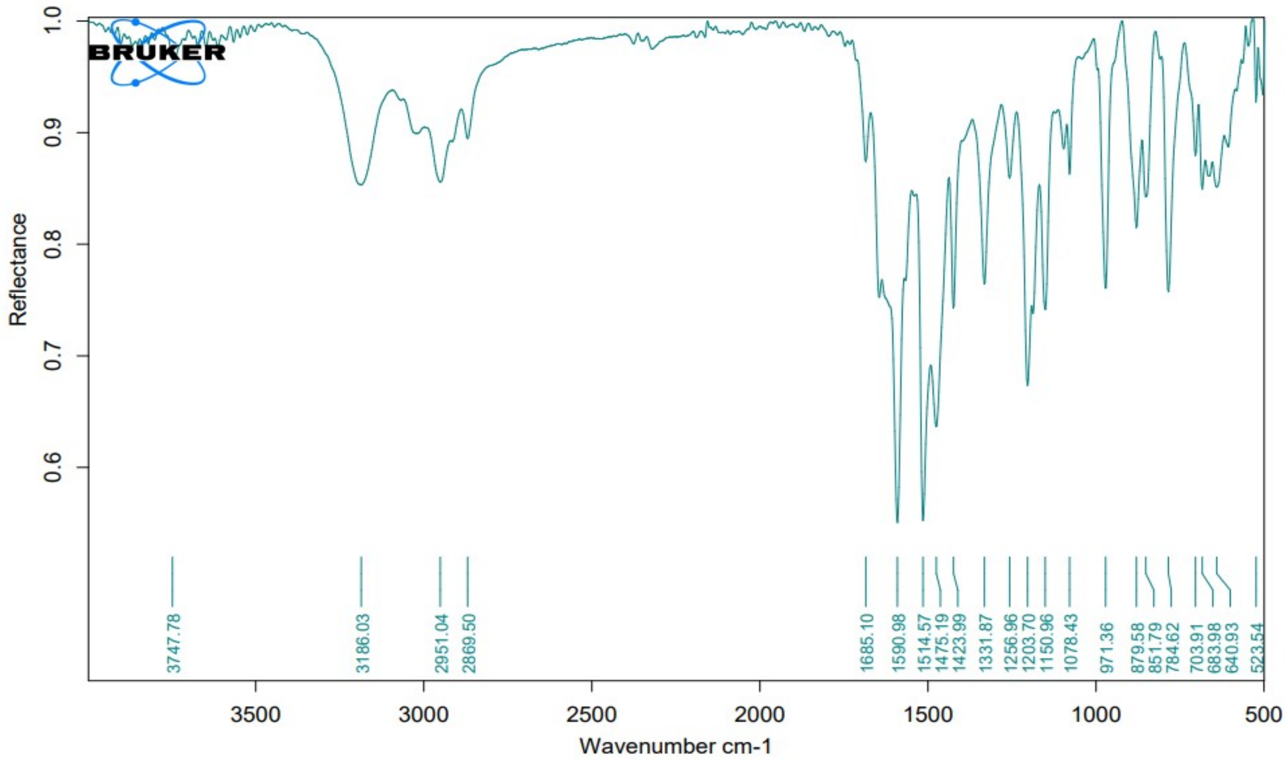

Figure S46: FT-IR spectrum of compound 7d

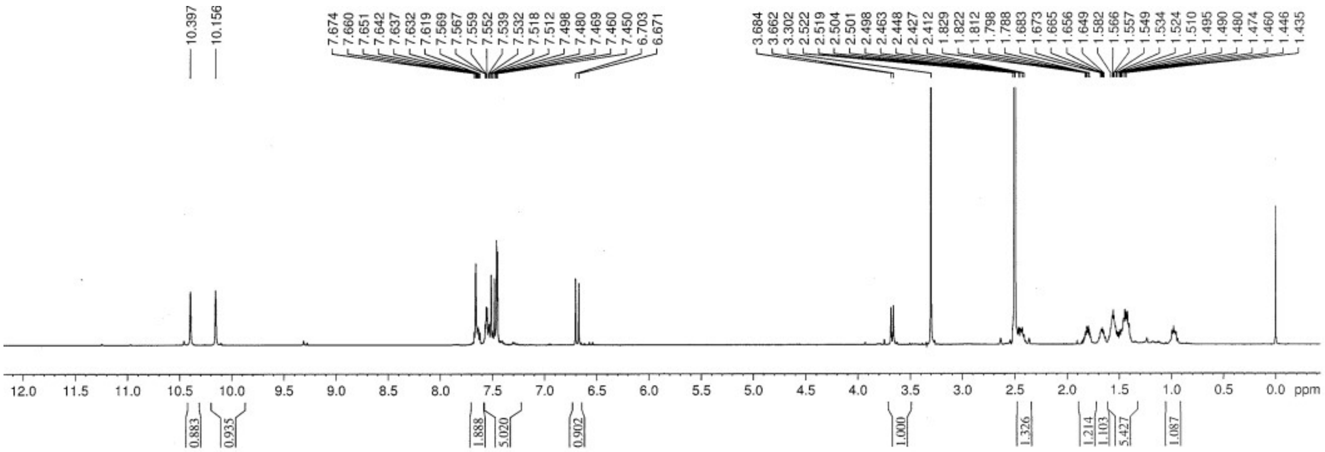

Figure S47: H[1] NMR spectrum of compound 7d

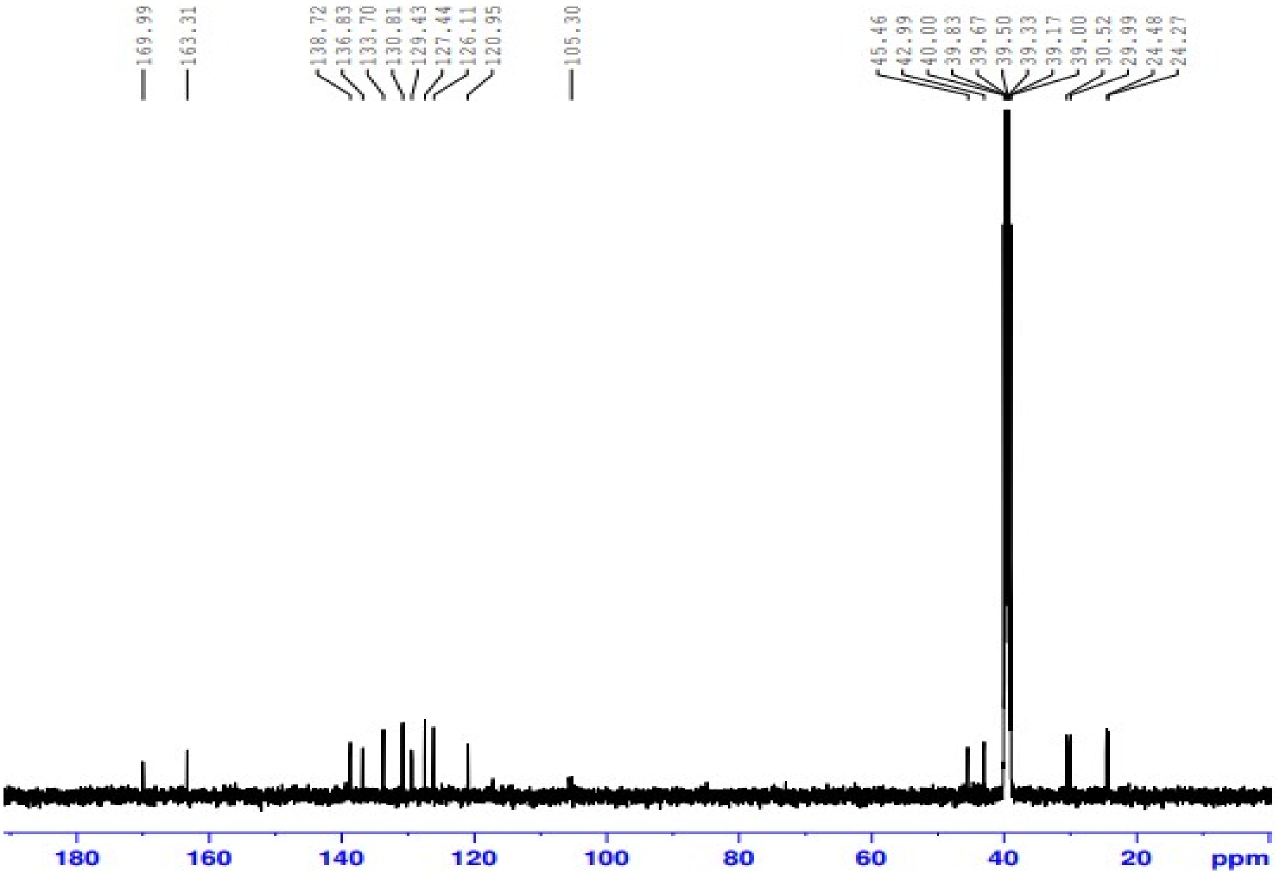

Figure S48: ^{13}C NMR spectrum (500 MHz, DMSO-d_6) spectrum of compound 7d

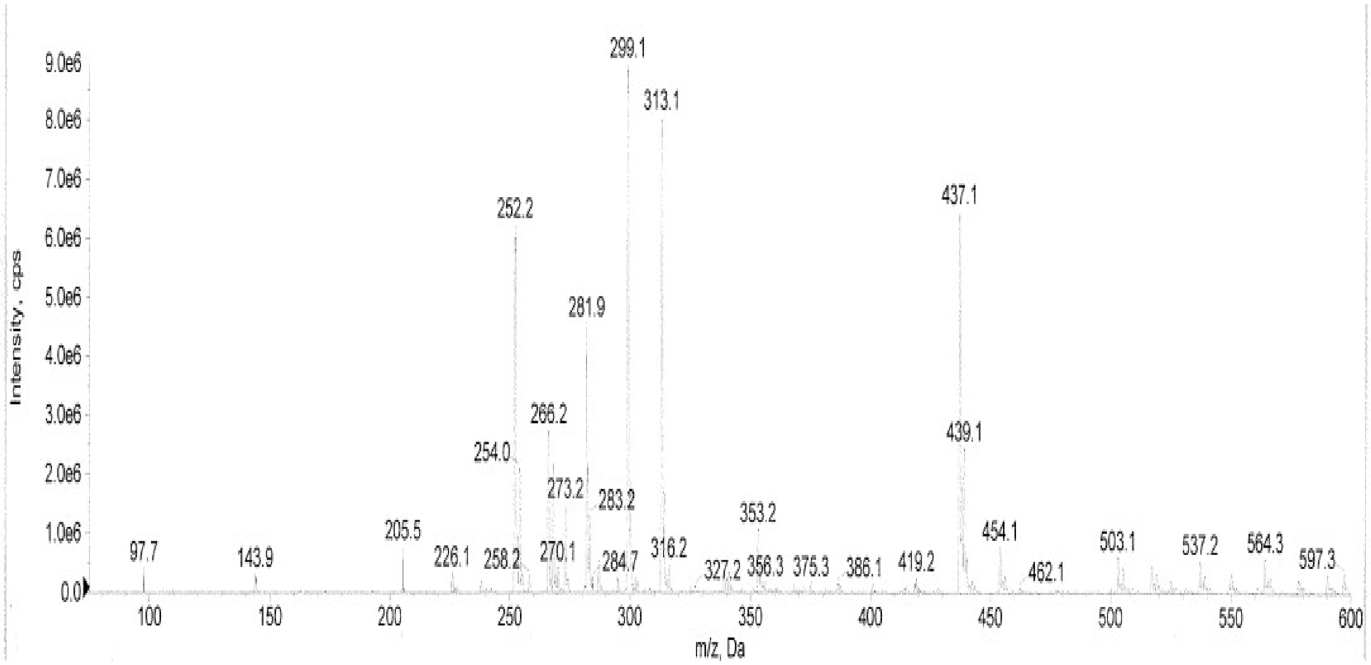

Figure S49: Mass spectrum of compound 7d

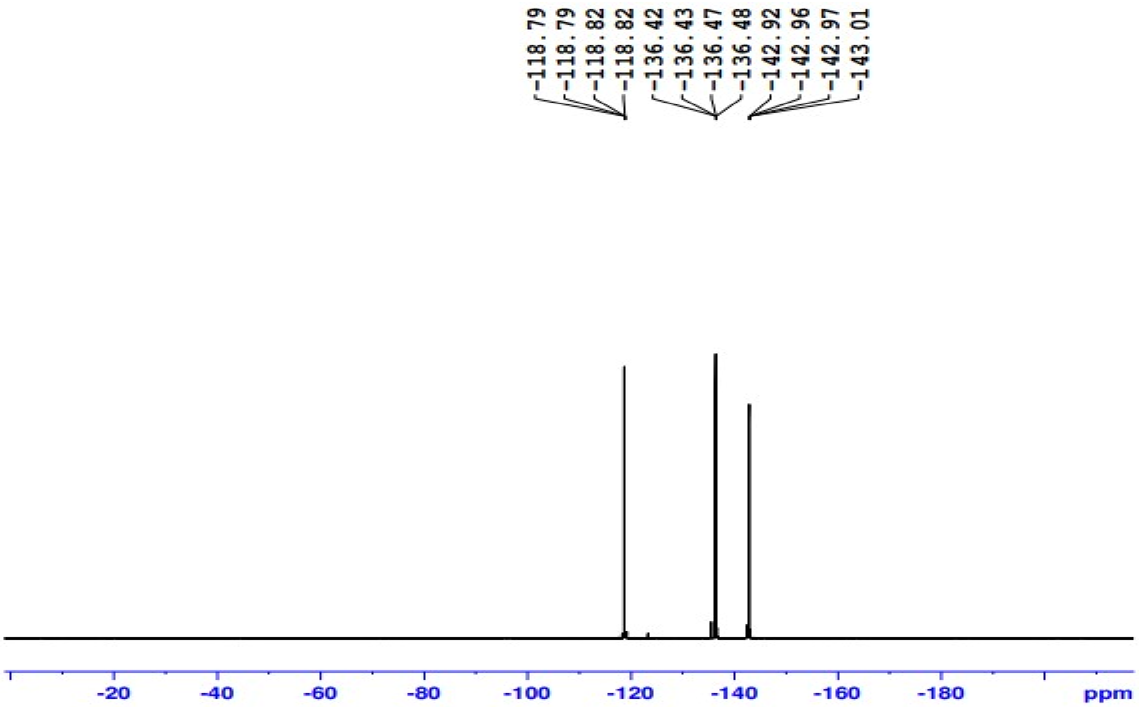

Figure S50: ^{19}F spectrum of compound 7d

N'-(3-(2-bromophenyl)acryloyl)-1-(p-tolyl)cyclopropane-1-carbohydrazide (7e):

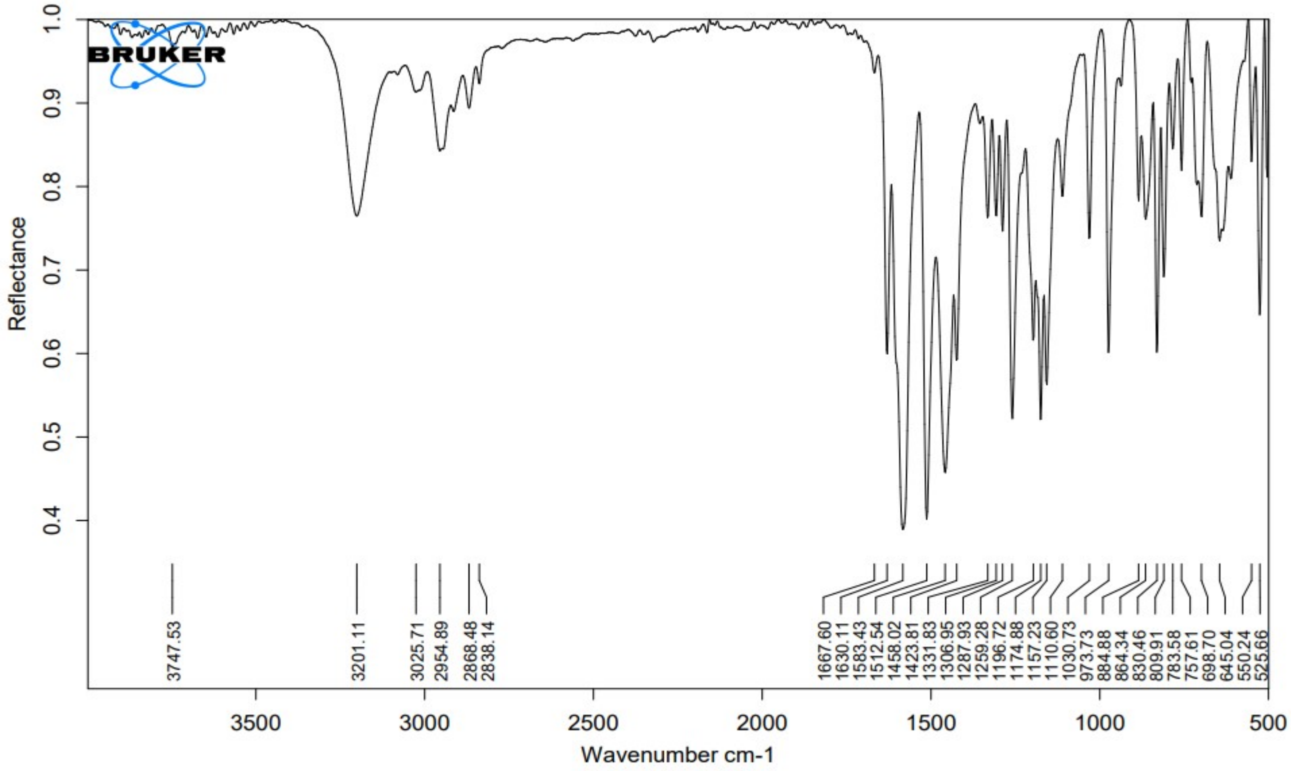

Figure S51: FT-IR spectrum of compound 7e

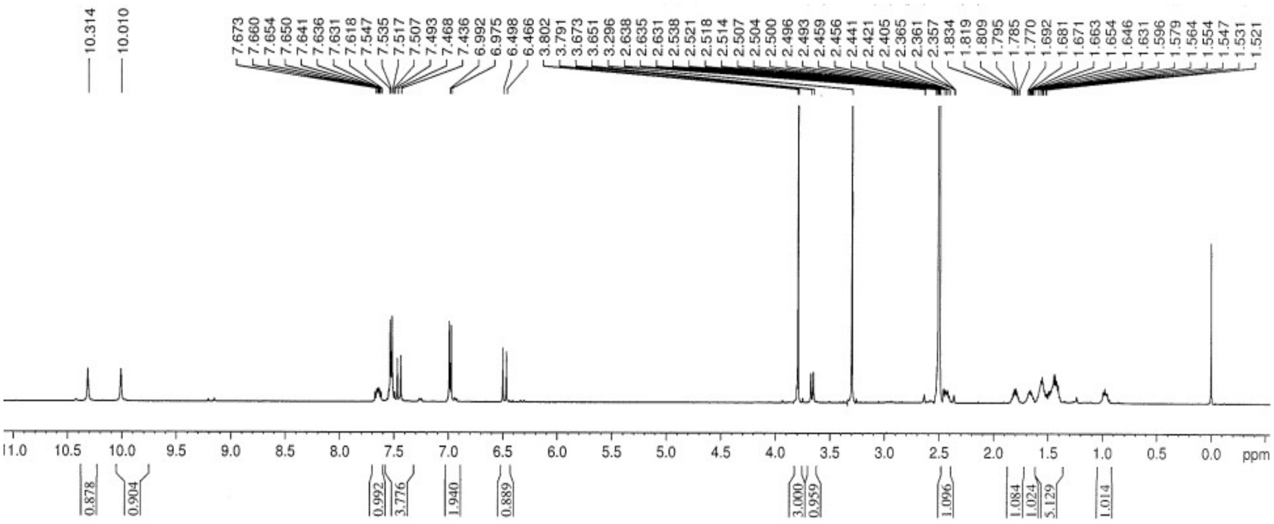

Figure S52: H[1] NMR spectrum of compound 7e

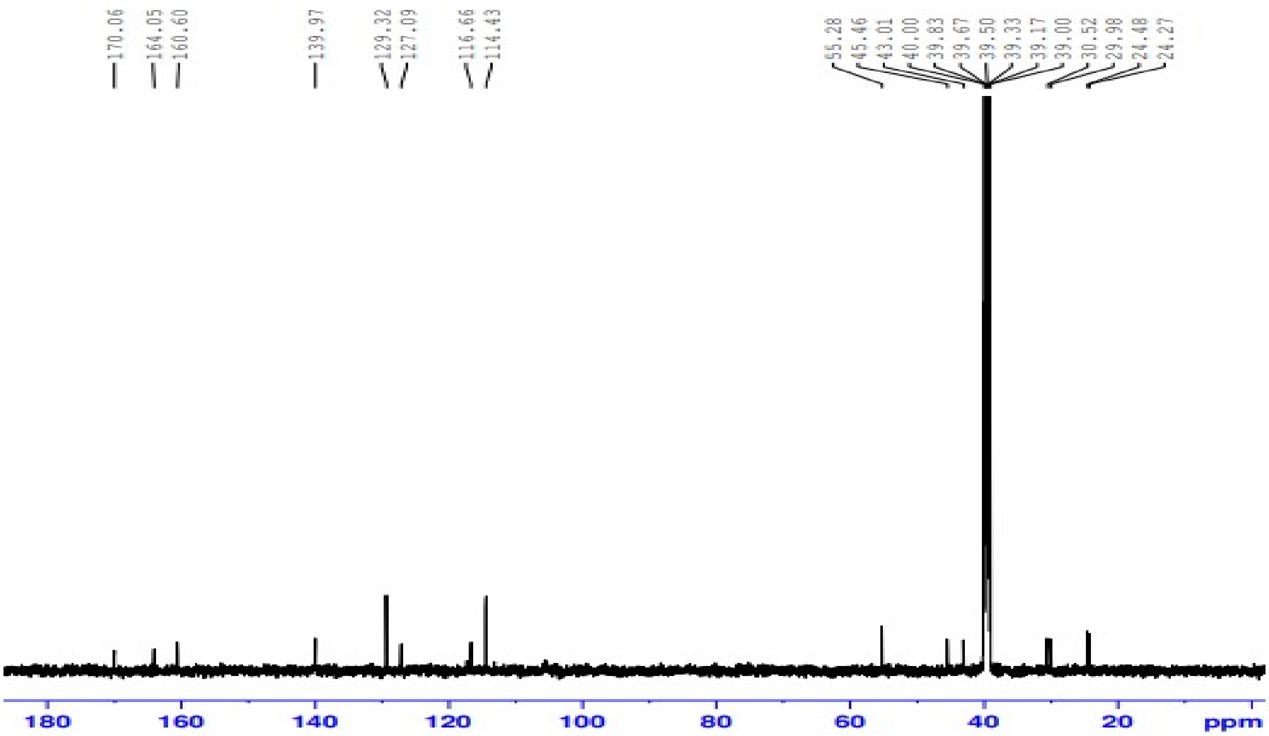

Figure S53: ^{13}C NMR spectrum (500 MHz, DMSO-d_6) spectrum of compound 7e

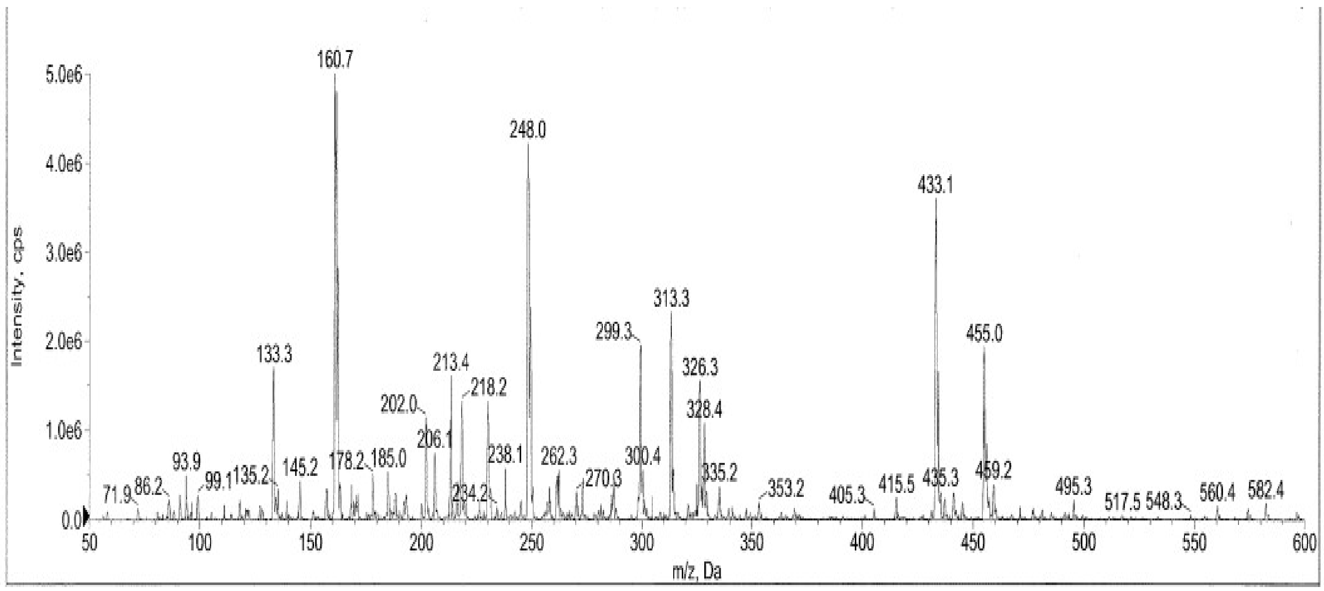

Figure S54: Mass spectrum of compound 7e

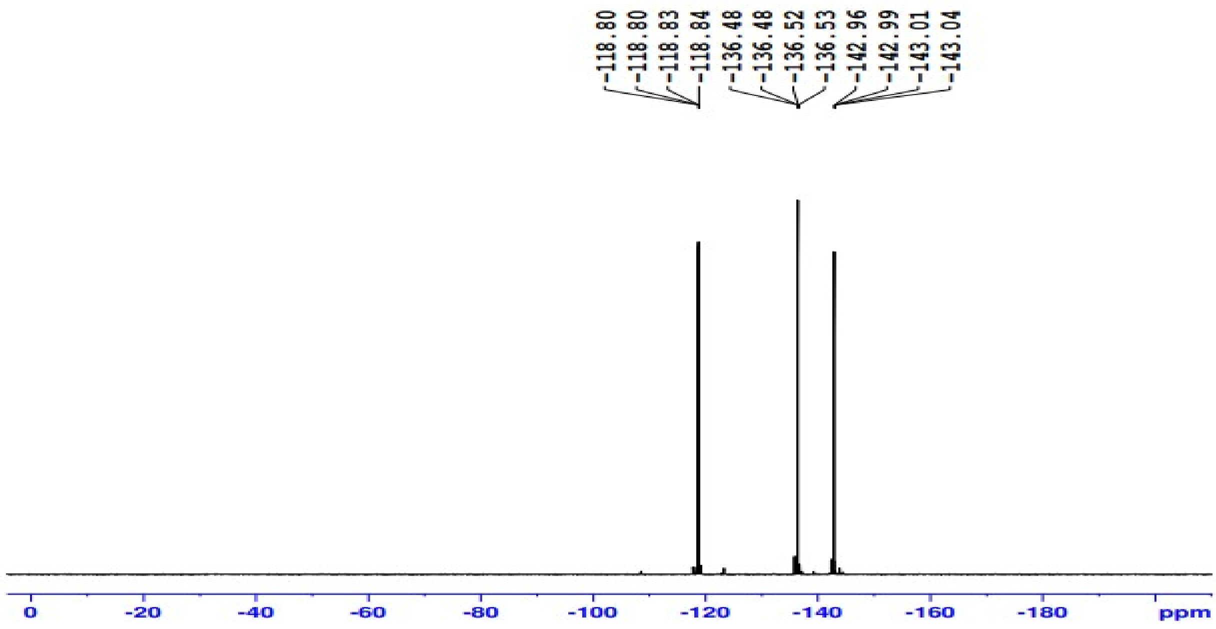

Figure S55: ^{19}F spectrum of compound 7e

***N'*-(3-(3-bromophenyl)acryloyl)-1-(p-tolyl)cyclopropane-1-carbohydrazide (7f):**

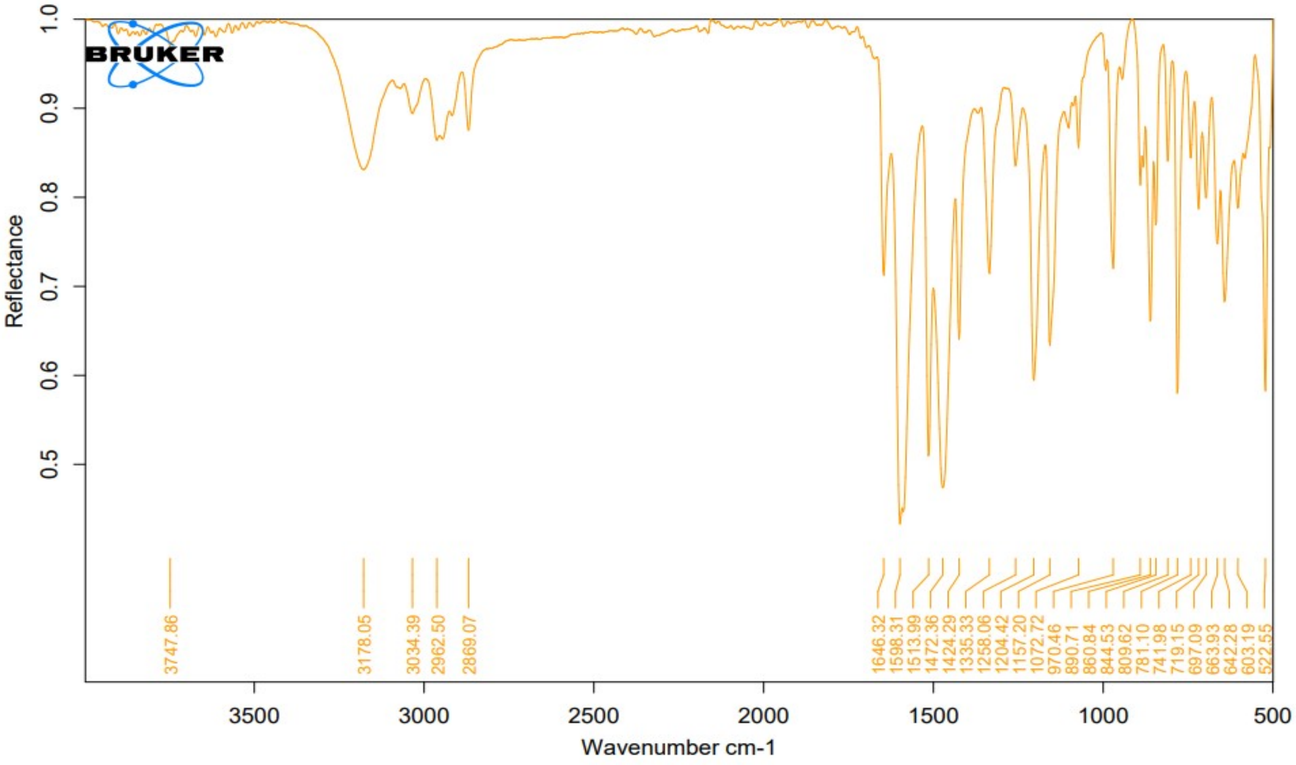

Figure S56: FT-IR spectrum of compound 7f

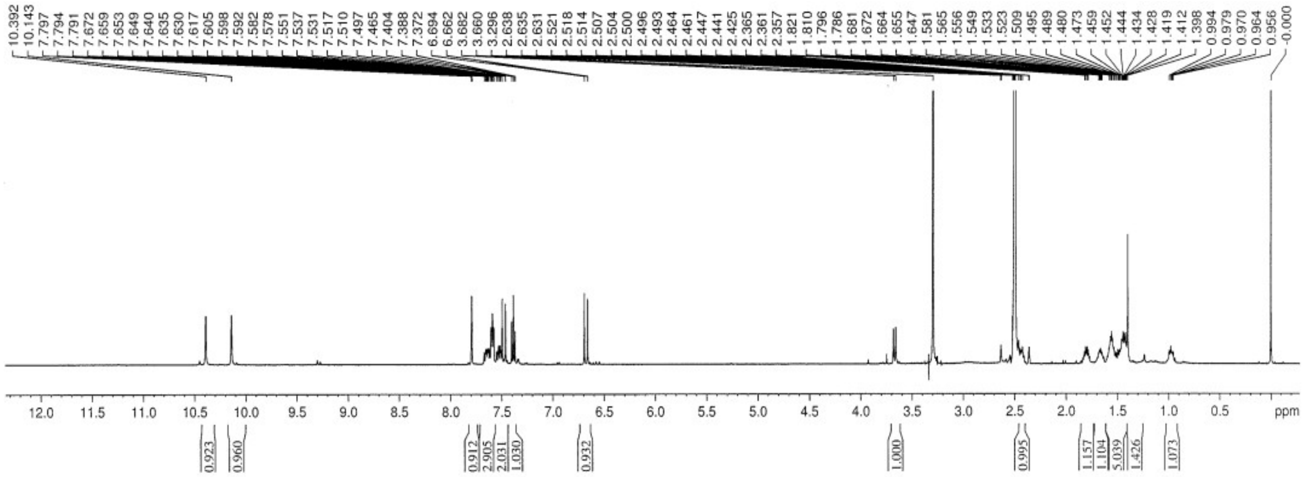

Figure S57: H[1] NMR spectrum of compound 7f

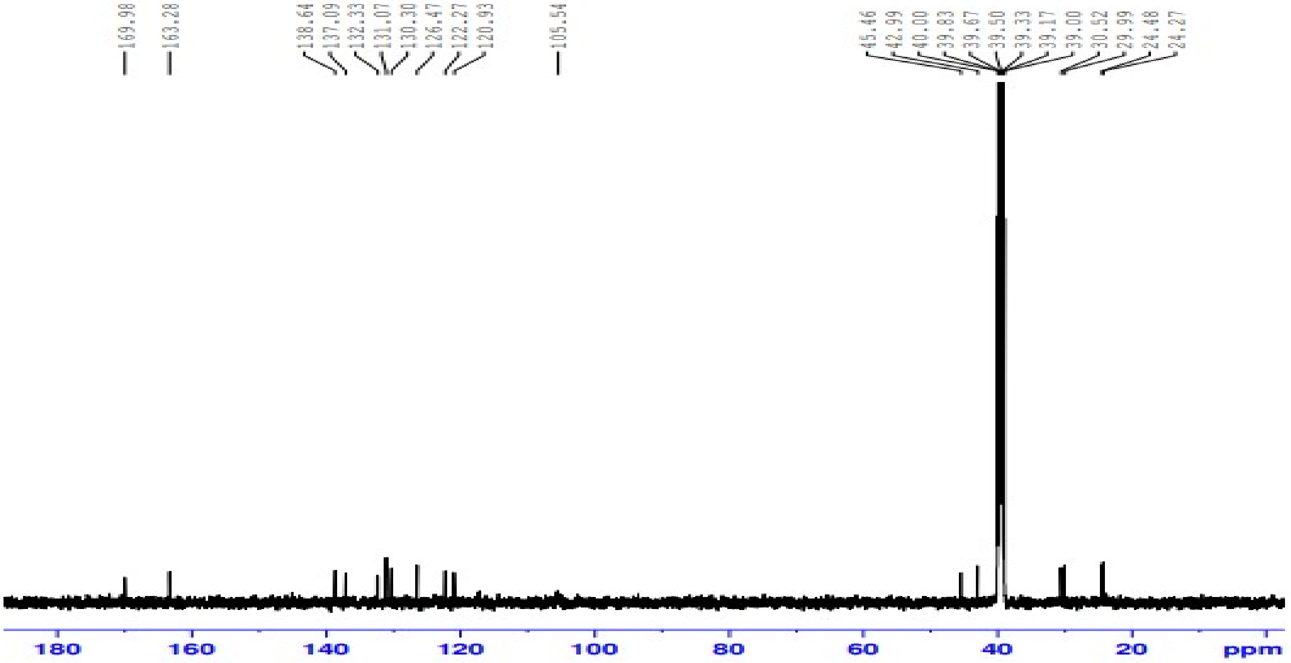

Figure S58: ^{13}C NMR spectrum (500 MHz, DMSO-d_6) spectrum of compound 7f

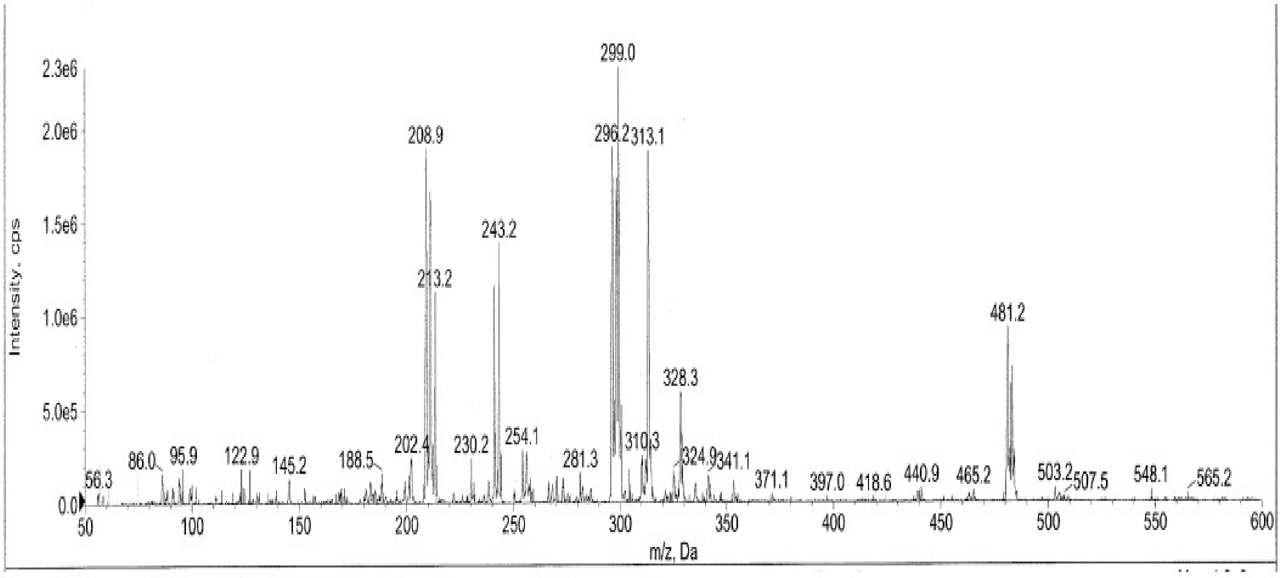

Figure S59: Mass spectrum of compound 7f

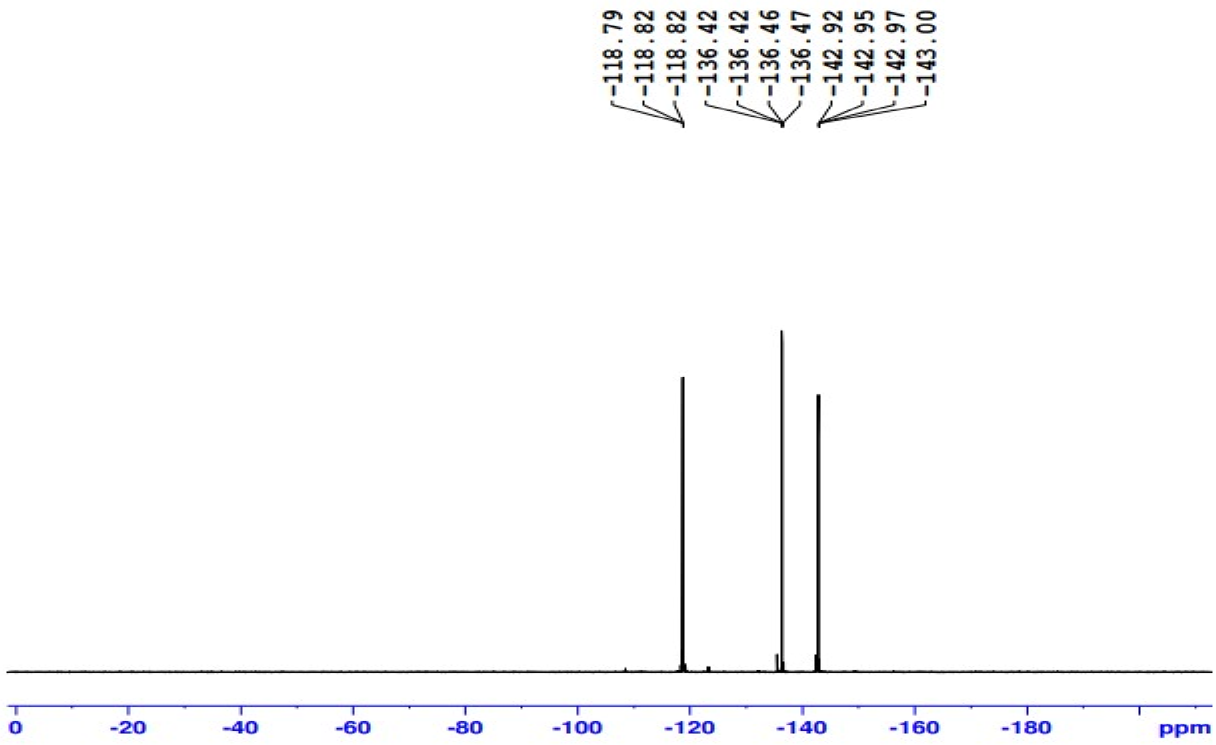

Figure S60: ^{19}F spectrum of compound 7f

***N'*-(3-(4-bromophenyl)acryloyl)-1-(p-tolyl)cyclopropane-1-carbohydrazide (7g):**

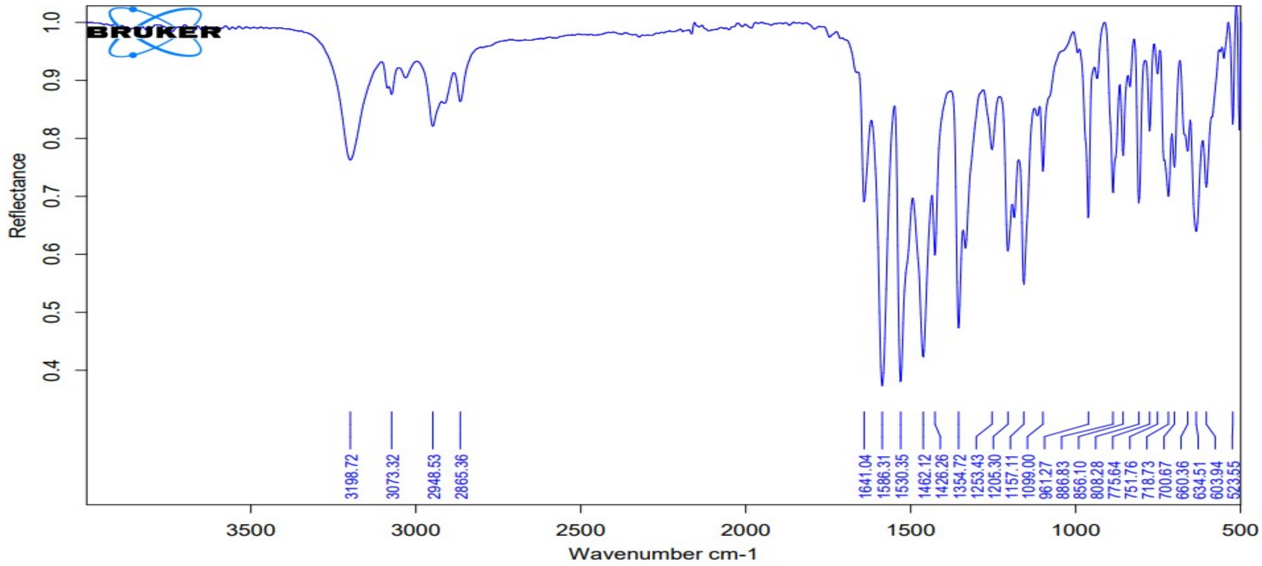

Figure S61: FT-IR spectrum of compound 7g

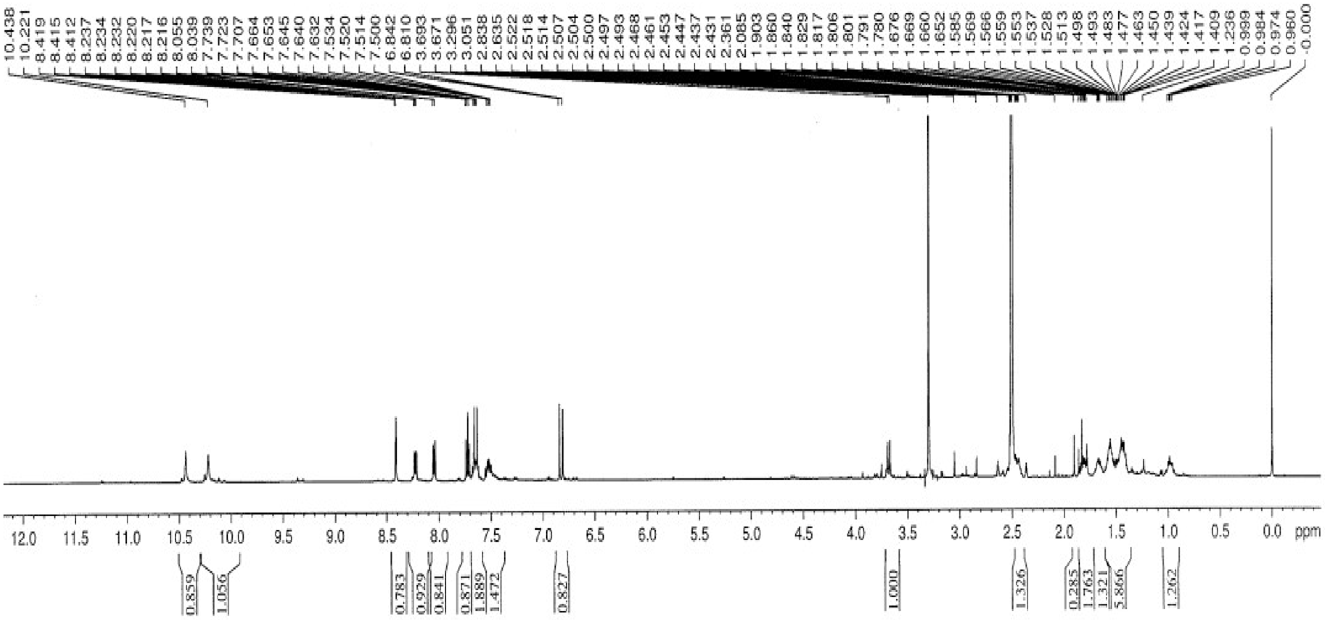

Figure S62: H[1] NMR spectrum of compound 7g

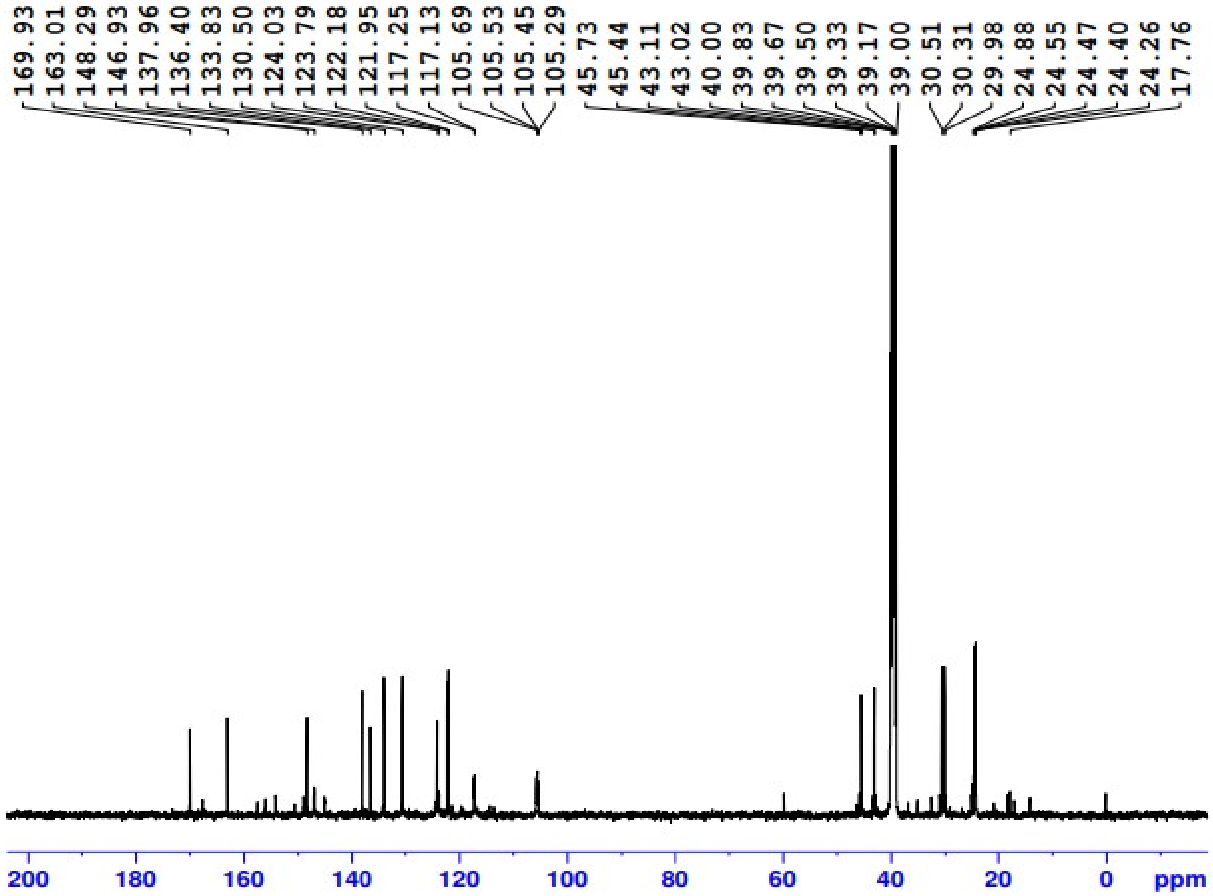

Figure S63: ^{13}C NMR spectrum (500 MHz, DMSO-d_6) spectrum of compound 7g

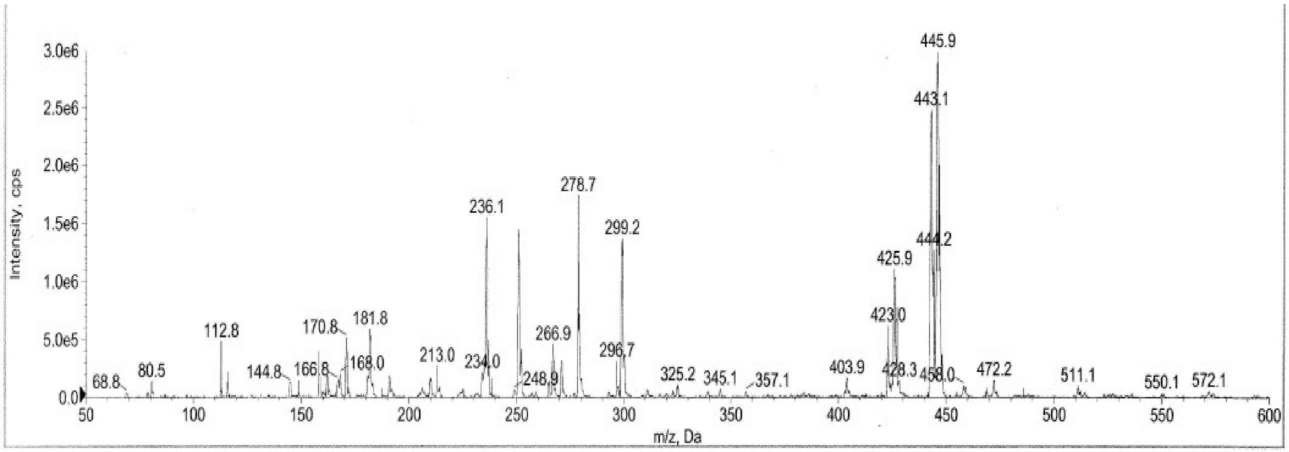

Figure S64: Mass spectrum of compound 7g

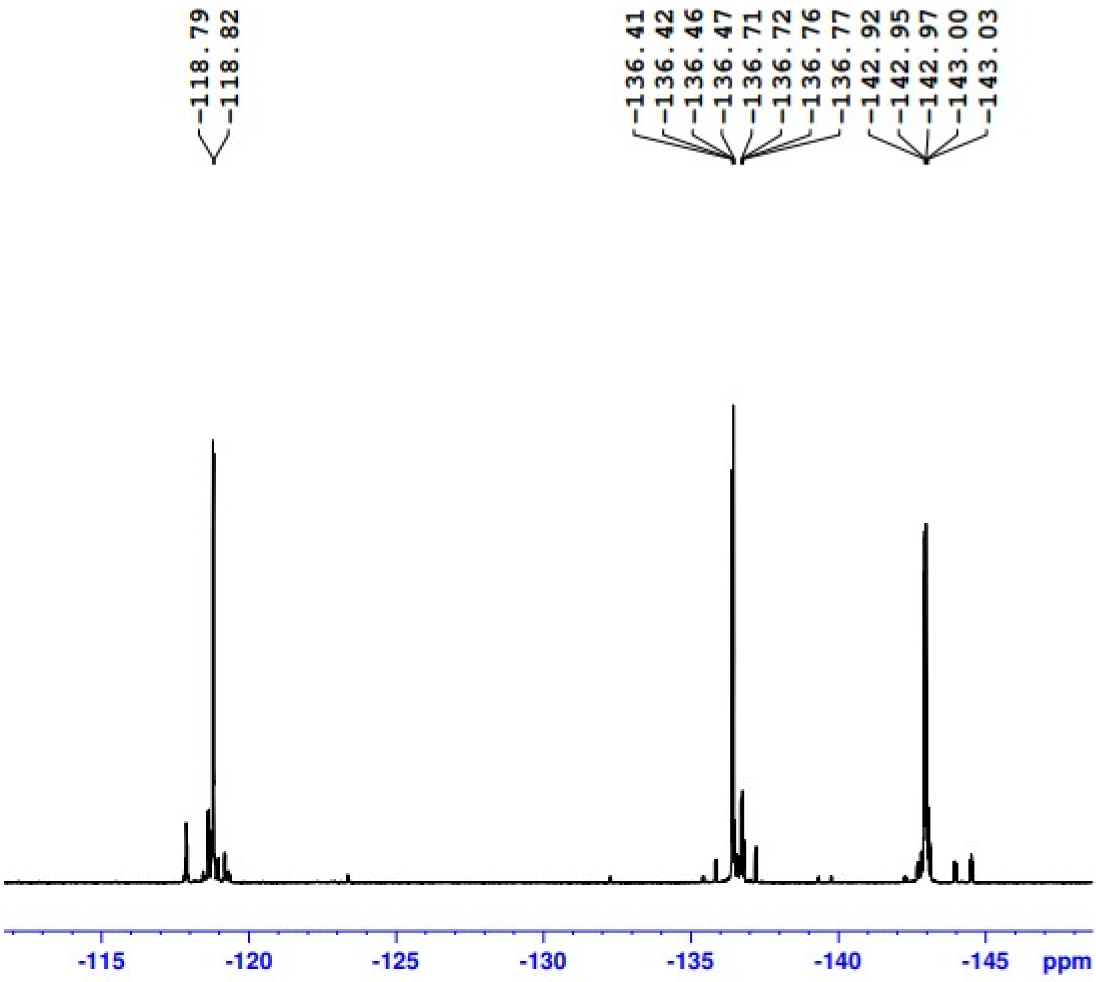

Figure S65: ^{19}F spectrum of compound 7g

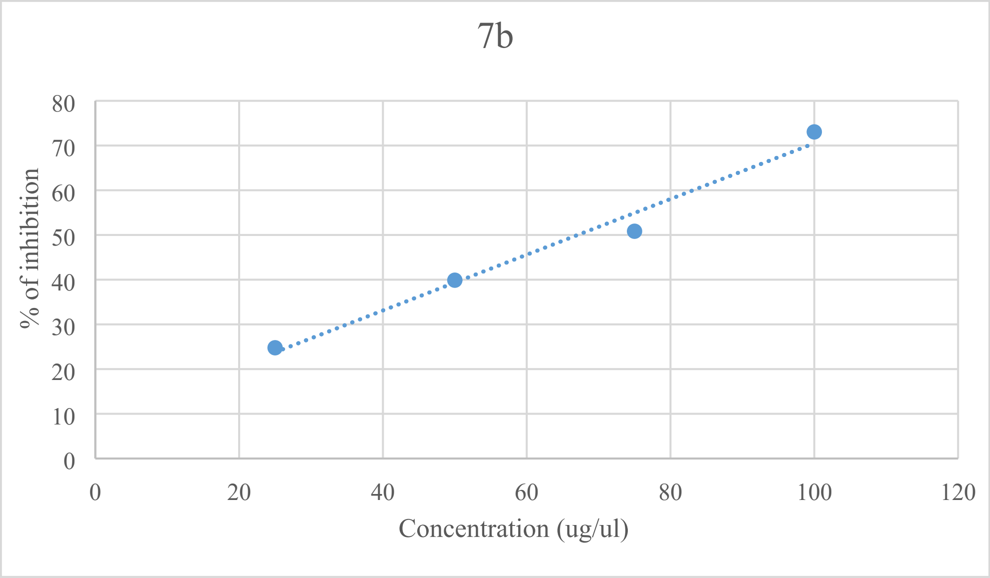

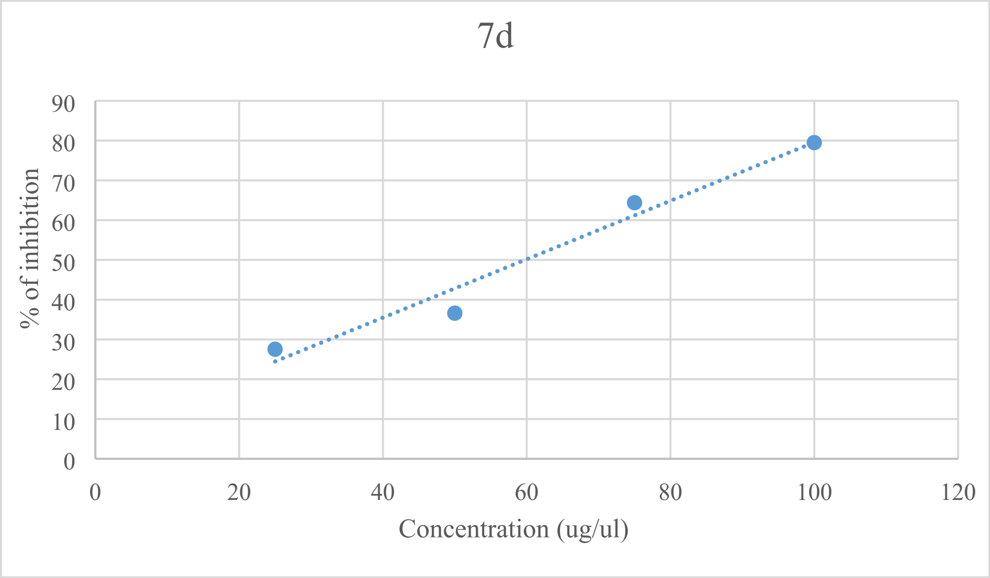

Figure S66: α-Glucosidase inhibitory effect of compounds **7b** and **7d**.
